# The mosaic oat genome gives insights into a uniquely healthy cereal crop

**DOI:** 10.1038/s41586-022-04732-y

**Published:** 2022-05-18

**Authors:** Nadia Kamal, Nikos Tsardakas Renhuldt, Johan Bentzer, Heidrun Gundlach, Georg Haberer, Angéla Juhász, Thomas Lux, Utpal Bose, Jason A. Tye-Din, Daniel Lang, Nico van Gessel, Ralf Reski, Yong-Bi Fu, Peter Spégel, Alf Ceplitis, Axel Himmelbach, Amanda J. Waters, Wubishet A. Bekele, Michelle L. Colgrave, Mats Hansson, Nils Stein, Klaus F. X. Mayer, Eric N. Jellen, Peter J. Maughan, Nicholas A. Tinker, Martin Mascher, Olof Olsson, Manuel Spannagl, Nick Sirijovski

**Affiliations:** 1 Plant Genome and Systems Biology, German Research Center for Environmental Health, Helmholtz Zentrum München, Neuherberg, Germany; 2 ScanOats Industrial Research Centre, Department of Chemistry, Division of Pure and Applied Biochemistry, Lund University, Lund, Sweden; 3 Australian Research Council Centre of Excellence for Innovations in Peptide and Protein Science, School of Science, Edith Cowan University, Joondalup, Western Australia Australia; 4 Agriculture and Food, Commonwealth Scientific and Industrial Research Organisation, St Lucia, Queensland Australia; 5 Immunology Division, Walter and Eliza Hall Institute of Medical Research, Parkville, Victoria Australia; 6 Department of Gastroenterology, Royal Melbourne Hospital, Parkville, Victoria Australia; 7 Plant Biotechnology, Faculty of Biology, University of Freiburg, Freiburg, Germany; 8 Plant Gene Resources of Canada, Agriculture and Agri-Food Canada, Saskatoon, Saskatchewan Canada; 9 Department of Chemistry, Centre for Analysis and Synthesis, Lund University, Lund, Sweden; 10 Plant Breeding, Lantmännen, Svalöv, Sweden; 11 Leibniz Institute of Plant Genetics and Crop Plant Research (IPK), Seeland, Germany; 12 Research and Development Division, PepsiCo, St Paul, MN USA; 13 Ottawa Research and Development Centre, Agriculture and Agri-Food Canada, Ottawa, Ontario Canada; 14 Molecular Cell Biology, Department of Biology, Lund University, Lund, Sweden; 15 Department of Crop Sciences, Center of Integrated Breeding Research (CiBreed), Georg-August-University, Göttingen, Germany; 16 School of Life Sciences Weihenstephan, Technical University of Munich, Freising, Germany; 17 Department of Plant and Wildlife Sciences, Brigham Young University, Provo, UT USA; 18 German Centre for Integrative Biodiversity Research (iDiv), Halle-Jena-Leipzig, Leipzig, Germany; 19 CropTailor AB, Department of Chemistry, Division of Pure and Applied Biochemistry, Lund University, Lund, Sweden; 20 Present Address: Department of Microbial Genomics and Bioforensics, Bundeswehr Institute of Microbiology, Munich, Germany; 21 Present Address: Food Science Organisation, Oatly AB, Lund, Sweden

**Keywords:** Agricultural genetics, Polyploidy in plants, Genomics, Evolutionary genetics, Plant breeding

## Abstract

Cultivated oat (*Avena sativa* L.) is an allohexaploid (AACCDD, 2*n* = 6*x* = 42) thought to have been domesticated more than 3,000 years ago while growing as a weed in wheat, emmer and barley fields in Anatolia^1,2^. Oat has a low carbon footprint, substantial health benefits and the potential to replace animal-based food products. However, the lack of a fully annotated reference genome has hampered efforts to deconvolute its complex evolutionary history and functional gene dynamics. Here we present a high-quality reference genome of *A*. *sativa* and close relatives of its diploid (*Avena longiglumis*, AA, 2*n* = 14) and tetraploid (*Avena insularis*, CCDD, 2*n* = 4*x* = 28) progenitors. We reveal the mosaic structure of the oat genome, trace large-scale genomic reorganizations in the polyploidization history of oat and illustrate a breeding barrier associated with the genome architecture of oat. We showcase detailed analyses of gene families implicated in human health and nutrition, which adds to the evidence supporting oat safety in gluten-free diets, and we perform mapping-by-sequencing of an agronomic trait related to water-use efficiency. This resource for the *Avena* genus will help to leverage knowledge from other cereal genomes, improve understanding of basic oat biology and accelerate genomics-assisted breeding and reanalysis of quantitative trait studies.

## Abstract (web summary)

Assembly of the hexaploid oat genome and its diploid and tetraploid relatives clarifies the evolutionary history of oat and allows mapping of genes for agronomic traits.

## Main

Oat is a member of Poaceae, an economically important grass family that includes wheat, rice, barley, common millet, maize, sorghum and sugarcane. *Avena* species exist in nature as diploids, tetraploids and hexaploids and exhibit the greatest genetic diversity around the Mediterranean, Middle East, Canary Islands and Himalayas. Currently, oat is a global crop with production ranking seventh among cereals (http://www.fao.org/faostat/en/, accessed May 2021). Compared with that of other cereals, oat cultivation requires fewer treatments with insecticides, fungicides or fertilizers. Whole-grain oats are a healthy source of antioxidants, polyunsaturated fatty acids, proteins and dietary fibre such as β-glucan, which is important in post-meal glycaemic responses and for preventing cardiovascular disease^3–5^. Cereals such as wheat, barley and rye store high amounts of gluten proteins in their grain; by contrast, oat and rice store globular proteins in their grain.

## Genome assembly and composition

**Fig. 1:**
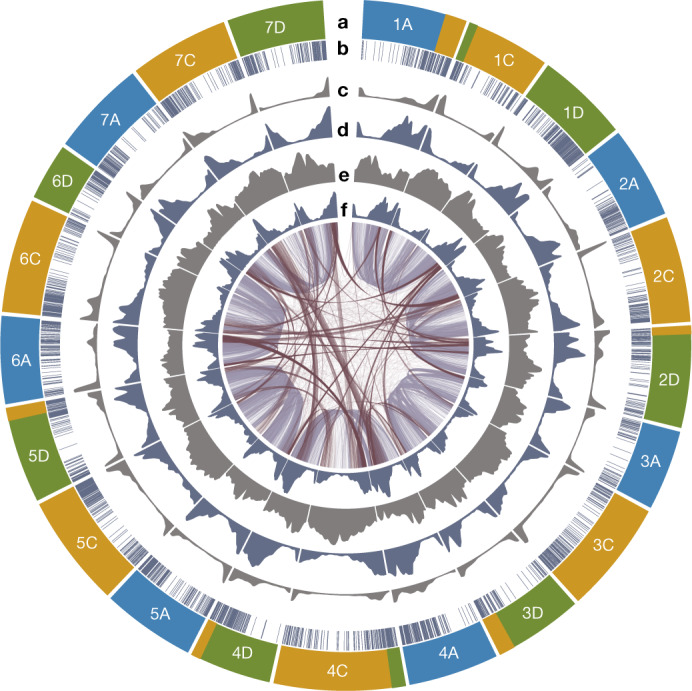
Structural and functional landscape of the 21 oat pseudochromosomes. **a**–**f**, The tracks from the outer circles towards the centre of the diagram display the chromosome name and subgenome origin (A, blue; C, gold; D, green) with major translocations (**a**); anchored oat genetic markers^51^ (**b**); distribution of recombination rates (**c**); density and genomic distribution of high-confidence genes (**d**); age distribution of long terminal repeat retrotransposons (**e**); and median gene expression in 1-Mb windows (**f**). Inner connections show the best bidirectional BLAST hits between genes on homoeologous chromosomes (grey) and between genes on non-homoeologous chromosomes (dark red). Figure generated with Circa (http://omgenomics.com/circa).

**Extended Data Fig. 1:**
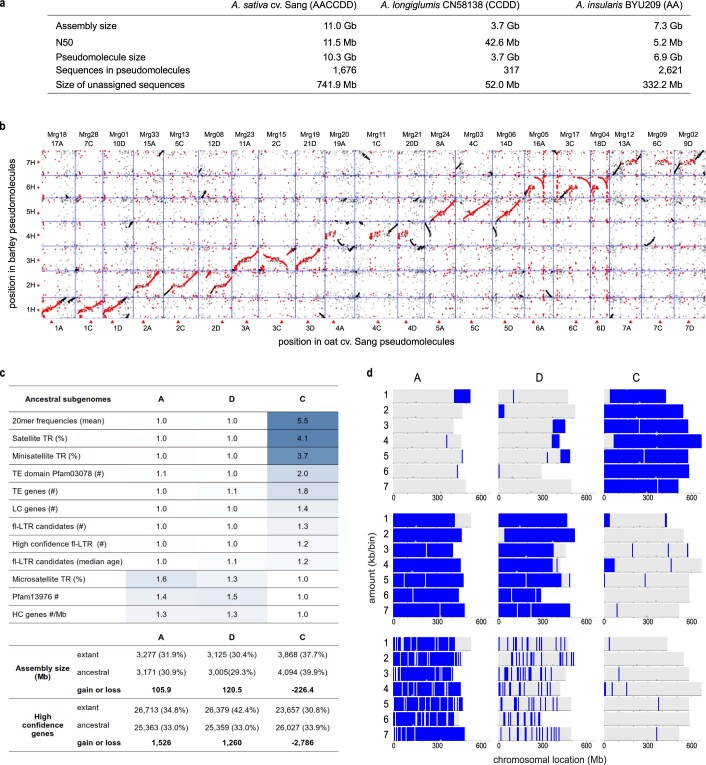
Assembly of three *Avena* species and chromosome organisation of hexaploid oat. **a**, Summary of assembly statistics of the hexaploid *A. sativa* cv. Sang, diploid *A. longiglumis* CN58138 and tetraploid *A. insularis* BYU209 genomes. **b**, Gene-based collinearity of oat (*A. sativa*) to barley (*H. vulgare*). Each data point is an aligned gene. Genes in core regions are shown in red. The phylogenetically informed nomenclature is used on the bottom x-axis. The top axis shows the chromosome names according to Bekele et al. (2018)^10^, (Mrg groups) and according to Sanz et al. (2010)^13^, respectively. Centromere positions in oat and barley are indicated by red triangles (x axis) and red diamonds (y axis). **c**, Subgenome composition of hexaploid oat cv. Sang. Subgenome-specific features related to independent transposon histories in the diploid ancestors. The upper part shows the x-fold increase compared to the lowest of the three values across subgenomes A–C. The lower part summarises differences in genome size and gene number between the extant hexaploid and the ancestral state predating the seven translocations between the tetraploid and hexaploid subgenomes. **d**, Tandem repeat families in hexaploid oat cv. Sang specific to either the C or the A/D diploid ancestor or enriched in the extant A subgenome (from top to bottom).

**Extended Data Fig. 2:**
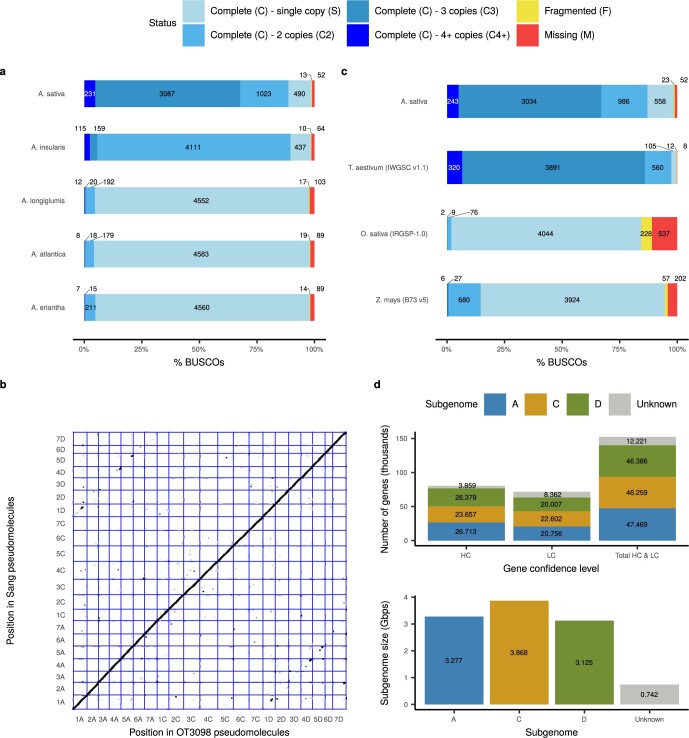
Validation and statistics of *A. sativa* cv. Sang assembly and gene annotation. **a**, BUSCO (lineage poales_odb10, created 2020-08-05) scores of the genome assemblies of *A. sativa* cv. Sang, *A. insularis* and *A. longiglumis* as well as the diploid progenitors *A. eriantha* and *A. atlantica* as a comparison. **b**, Colinearity plot of the pseudomolecules of *A. sativa* cv. Sang (short read assembly, y-axis) and OT3098 (long read assembly, x-axis). **c**, BUSCO (lineage poales_odb10, created 2020-08-05) scores for the oat reference gene annotation v1.1 in comparison to the results of recent gene predictions for maize, rice and bread wheat. **d**, *Top:* overview of the number of predicted genes (HC - high confidence; LC - low confidence) for the three oat subgenomes and unplaced/unknown scaffolds. *Bottom:* total number of basepairs assembled for the respective three subgenomes and the number of basepairs in unplaced scaffolds.

We produced a chromosome-scale reference sequence of oat cv. ‘Sang’ comprising 21 pseudochromosomes (Fig. 1, Extended Data Fig. 1a and Supplementary Table 1), with a BUSCO (v5.1.2; ref. ^6^) score of 98.7% (Extended Data Fig. 2a), following the short-read strategy used for wheat^7^, barley^8^ and rye^9^. Inspection of Hi-C contact matrices (Supplementary Fig. 1) and the consensus genetic map^10^ (Supplementary Fig. 2a) and their comparison with the independent assembly (long-read) of hexaploid oat OT3098 (ref. ^11^; version 2; Supplementary Table 2) verified the integrity of the assembly (Extended Data Fig. 2b and Supplementary Fig. 2). We also assembled pseudochromosomes of the diploid *Avena longiglumis* and tetraploid *Avena insularis*, which are presumed A and CD subgenome progenitors of *Avena sativa*^12^ (Extended Data Figs. 1a and 2a and Supplementary Figs. 3 and 4). Phylogenomic analyses (Supplementary Fig. 5) used to assign *A. sativa* chromosomes to subgenomes showed that gene order is conserved in the proximal chromosomal regions. The 21 *A. sativa* chromosomes, named 1A–7D following the subgenome assignments of ref. ^13^, were oriented to preserve the orientations of core regions across homoeologues and possibly between *Avena* and Triticeae. Alignments to barley (Extended Data Fig. 1b), *Avena eriantha*^14^ (Supplementary Fig. 6), *A*. *longiglumis* (Supplementary Fig. 7a) and *A*. *insularis* (Supplementary Fig. 7b) confirmed the validity of this revised nomenclature, which is accepted by the International Oat Nomenclature Committee^15^.

We predicted gene models in the oat genome using an automated annotation pipeline^16^, assisted by RNA-sequencing (RNA-seq) and Iso-seq transcriptome data, protein homology and ab initio prediction. This yielded 80,608 high-confidence protein-coding loci (98.5% BUSCO; Extended Data Fig. 2c and Supplementary Table 3), 83.5% of which showed evidence of transcription in at least one condition. Another 71,727 low-confidence protein-coding loci primarily represent gene fragments, pseudogenes and gene models with weak support. The overall amount and composition of transposable elements is very similar between the Sang and OT3098 assemblies (Supplementary Tables 4 and 5 and Supplementary Fig. 8). Transposable elements accounted for 64% of the oat genome sequence. The size difference of about 1 Gb observed between the C and A or D subgenome probably reflects higher transposon activity in the diploid ancestor of the C subgenome, as evidenced by a 1.3-fold increase in the number of full-length long terminal repeat retrotransposons, an enrichment in specific transposable element-related Pfam domains and Csubgenome specific transposon families, higher repetitivity, more tandem repeats and higher numbers of transposable element and ﻿low-confidence genes (Extended Data Fig. 1c). Several tandem repeat subfamilies were unequally distributed across the subgenomes, highlighting potentially rearranged genomic regions (Extended Data Fig. 1d). However, limitations of the short-read assembly arising from lower contiguity (Supplementary Table 1) were apparent in the overall reduced representation of tandem repeats and ribosomal DNA loci (Supplementary Tables 4 and 6) as well as in regions of reduced gene density mainly in centromeric and pericentromeric regions and unplaced genes (Supplementary Fig. 9, Supplementary Table 7 and Supplementary Methods).

## Mosaic chromosome architecture of oat

**Fig. 2:**
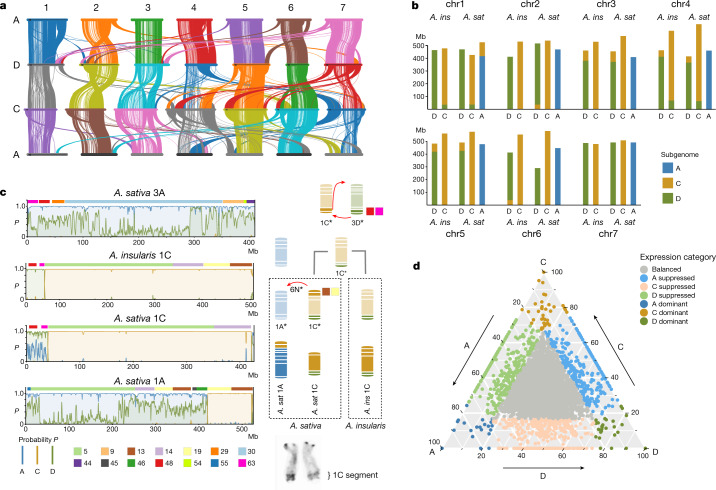
Genome organization, rearrangement and subgenome interplay in oat. **a**, Overview of syntenic blocks across the three subgenomes^52^. **b**, Predicted borders of the seven major inter-subgenomic translocations in hexaploid oat *A. sativa* (*A*. *sat*) and its closest tetraploid ancestor, *A. insularis* (*A*. *ins*). Blue, gold and green colours represent the A-, C- and D-subgenomic regions, respectively. **c**, Reconstruction of translocations in *A*. *sativa* and *A*. *insularis* using subgenome-specific *k*-mers and syntenic blocks and orthoblocks. Left side, probabilities of the A-, C- and D-subgenome classification by *k*-mers for chromosome 1C of *A*. *insularis* and chromosomes 3A, 1C and 1A of *A*. *sativa*. The boxes above each plot show the order and identity of colour-coded blocks of the respective orthologous homoeologous genes according to the colour bar at the bottom left. Right side, illustration of two translocation events deduced from the information at left: translocation of blocks 48 and 63 from chromosome 3D to 1C, which is shared by *A*. *sativa* and *A*. *insularis* and occurred in their tetraploid ancestor, and the transfer of blocks 13 and 19 from chromosome 1C to 1A in *A*. *sativa*, resulting in a duplication pattern of these blocks. The ancestral location of blocks 48 and 63 on chr3 is supported by chr3A of *A*. *sativa* (top left); chr3C of *A*. *sativa*, *A*. *insularis* and *A*. *eriantha*; and chr3A of *A*. *longiglumis*. Bottom right, the 1C segment of chr1A in *A. sativa* is cytologically highlighted. Asterisks refer to the ancestral state of chromosomes. **d**, Ternary plot of the relative expression levels of 7,726 ancestral triads (23,178 genes) in hexaploid oat in a combined analysis of all transcriptome samples. Each dot represents a gene triad with an A, C and D coordinate. Subgenome-dominant categories are defined by triads in vertices, whereas suppressed categories are associated with triads near edges and between vertices. Grey dots in the centre indicate balanced triads.

**Extended Data Fig. 3:**
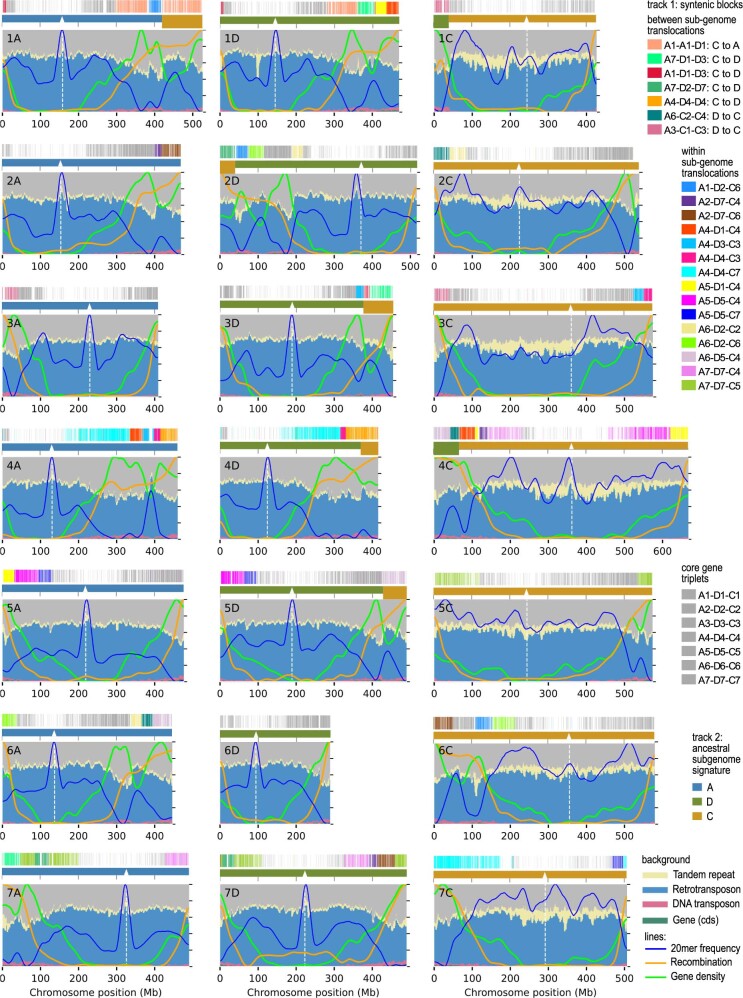
Chromosome architecture of hexaploid oat. Distribution of main features along the 21 Sang chromosomes. Track 1 displays syntenic blocks based on the chromosome pattern of gene clusters with exactly three members. Track 2 denotes the sub genome assignment. The background of the bottom part consists of a stacked bar chart for the major genome components. The lines in the front show smoothed chromosomal distributions for mean 20-mer frequency, average recombination rate (cM/Mb) and gene density (#/Mb), each scaled min to max per chromosome. The population-level analysis of crossover frequency is explored in (N.T., W.B. et al. ^21^). Therein we focus on average recombination rates that may result from global chromosome restructuring within *Avena*. Of particular interest are the long stretches of increased recombination on the long arms of chromosomes 4A and 4D. These regions coincide with stretches of high gene density and reduced transposon frequency. Other regions containing multiple ancestral telomeric regions showed corresponding multiple peaks of gene density and recombination. Also of interest is a region of suppressed recombination on chromosome 7D which is speculated to result from intraspecific rearrangements on this chromosome that impede meiotic pairing and crossovers.

**Extended Data Fig. 4:**
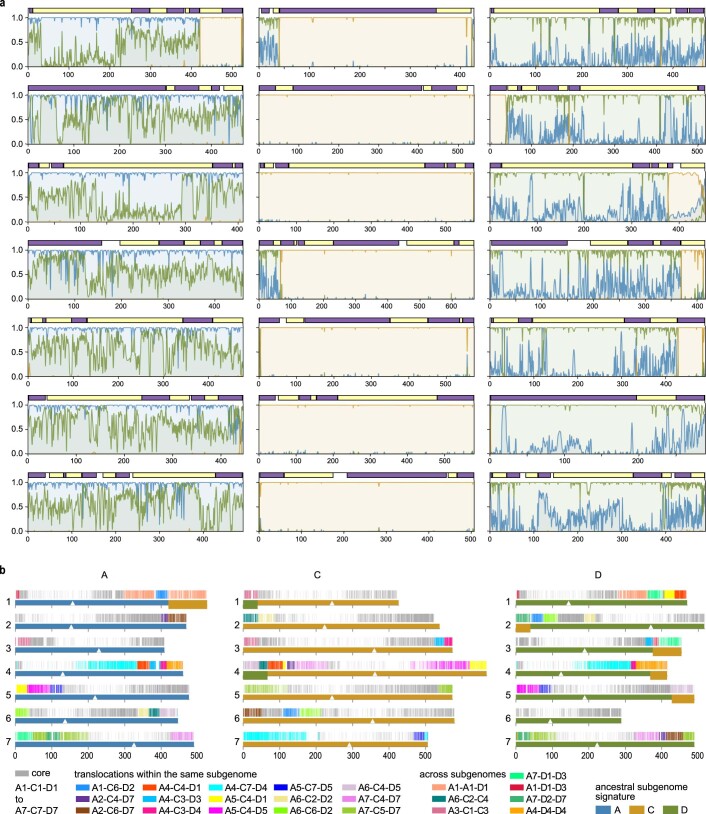
Mosaic composition of hexaploid oat chromosomes. **a**, Subgenome-specific kmers and syntenic blocks in *A. sativa*. Probabilities of A- (blue), C- (yellow) and D- (green) subgenome classification by subgenomic-kmers are shown. Top row in each subplot displays identified synteny shown in alternative colours to emphasise block borders. From top to bottom, chromosomes 1 to 7 for subgenomes A (left column), C (mid column) and D (right column), Y-axis: kmer-probability, x-axis: chromosomal position in Mb. **b**, Ancestral subgenome origin. In the extant Sang genome seven peripheral regions ranging from 40 Mb to 106 Mb differ in their subgenome signature from their current location. Five of these regions are of C genome origin: chr1A (106 Mb), chr2D (40Mb), chr3D (79 Mb), chr4D (46 Mb), chr5D (62 Mb). Two regions on the extant C subgenome are of D genome origin: chr1C (40 Mb) and chr4C (67 Mb). All in all, the transfers between subgenomes add up to 441 Mb, with a net loss in the C subgenome of 226 Mb. The upper tracks display homoeologous syntenic blocks within the Sang genome based on the chromosome pattern of gene clusters with exactly three members. The pattern A1-A1-D1 corresponds for example to a cluster with two members from chr1A and one member from chr1D. The core denotes all seven symmetric cluster patterns from A1-D1-C1 up to A7_D7_C7 with one member on each homoeolog chromosome (equivalent to the red dots in Extended Data Fig. 1b).

**Extended Data Fig. 5:**
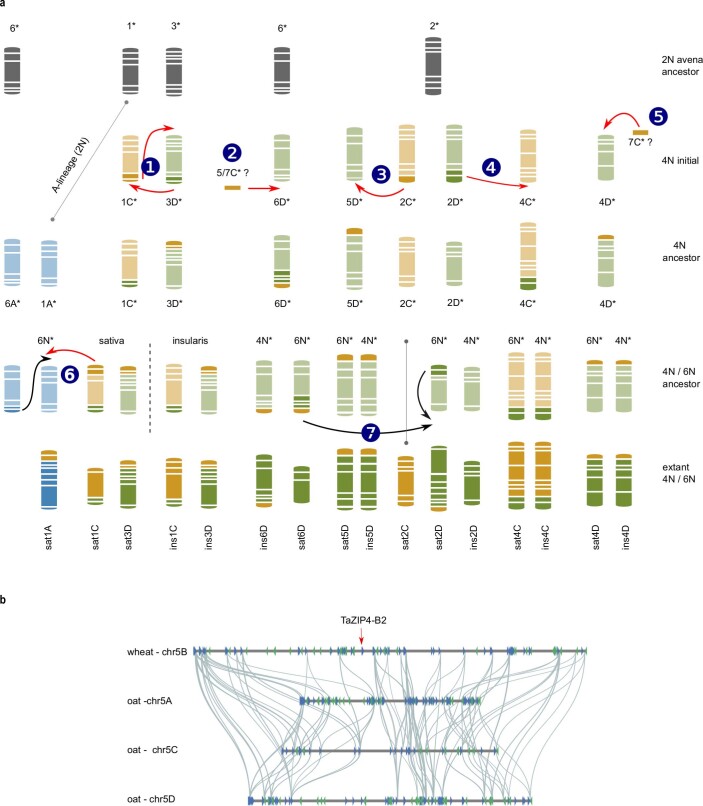
Genome rearrangements in oat. **a**, Translocation history in *A. insularis* and *A. sativa* cv*. Sang*. Top panel displays likely ancestral syntenic blocks for *Avena* chromosomes 1, 2, 3 and 6, for chromosomes 4, 5 and 7 we only provide ancestral states in the A-, D- and C-lineages separately. From top to bottom, subsequent panels show chromosomes and translocation operations for the initial tetraploid (directly after the hybridization of the diploid ancestors), the ancestral tetraploid after translocations, translocation events occurring after the split of *A. insularis* and oat, and the extant states. Genomic positions for each inter-subgenomic translocation in the extant tetra- and hexaploid are provided in Supplementary Table 8. A-, C- and D-subgenomic blocks are coloured in blue, yellow and green, respectively. Blocks contributing to translocations are accented in the mid panels by darker colours. Circled numbers represent translocation events: (1) represents two non-reciprocal translocations between the ancestral 1C and 3D chromosomes, (2) to (5) are additional translocations shared by *A. sativa* and *A. insularis*, while (6) and (7) are translocation differentiating extant oat and *A. insularis*. Asterisks refer to the ancestral state of chromosomes. **b**, Schematic representation of syntenic genomic regions between bread wheat (chromosome 5B harbouring *TaZIP4-B2*) and oat (chromosomes 5A, 5C, 5D). At the syntenic position, a *TaZIP4-B2* ortholog is absent in the oat genome.

The overall structure of the oat genome is similar to that of Triticeae genomes, although frequent genomic rearrangements in oat have resulted in a mosaic-like genome architecture. In many oat chromosomes, gene and recombination density is not a monotonic function of distance from the centromere (Extended Data Fig. 3), as is mostly observed in the Triticeae^17^. Examination of whole-genome alignments, subgenome-specific *k*-mers and orthologous and homoeologous genes clustering as syntenic blocks in genomic neighbourhoods in four *Avena* species (Extended Data Figs. 1d and 4) revealed numerous large-scale genomic rearrangements affecting the order of these blocks within and between subgenomes (Fig. 2a). We detected seven large-scale genomic rearrangements in *A*. *sativa* and traced them back to eight translocation events between the A, C and D subgenomes (Fig. 2b, c, Extended Data Figs. 4a and 5a, Supplementary Fig. 10 and Supplementary Table 8), spanning 4.3% of the genome and approximately 7.9% of the high-confidence genes. Two of the translocation events were specific to *A*. *sativa*. Unlike those in wheat^7^, the oat subgenomes exhibit unbalanced gene counts; specifically, the C subgenome appears to have 12% fewer genes than the A or D subgenome (Extended Data Fig. 2d and Supplementary Table 9). Analysis of orthologous gene groups (Supplementary Table 10 and Supplementary Figs. 11 and 12) showed that unbalanced gene families were associated with significant spatial clustering (Supplementary Fig. 13) in genomic rearrangements. Ancestral state reconstruction of the oat chromosomes revealed a loss of at least 226 Mb of gene-rich regions from the C subgenome to the A and D subgenomes (Supplementary Table 9). This implies that the translocations fully account for the lower gene count in the C subgenome and not gene loss or subfractionation after formation of the hexaploid.

**Extended Data Fig. 6:**
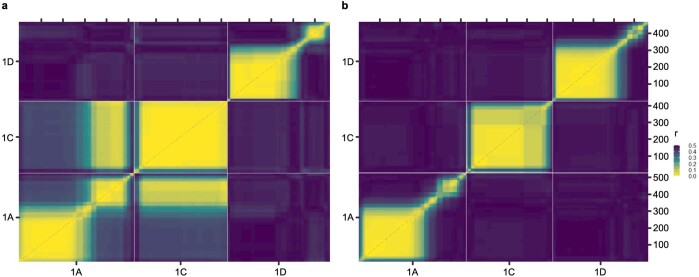
An example of a hidden breeding barrier in *Avena sativa* revealed by recombination matrices in two oat populations. Average pairwise recombination frequencies (r) among chromosomes 1A, 1C and 1D were computed in 16 Mbp windows at 1 Mbp increments for two RIL populations and are visualised as blended colours of yellow (r = 0) to cyan (r = 0.25) to burgundy (r = 0.5). Blocks of yellow along the diagonal dashed lines indicate recombination suppression within a chromosome. Blocks of yellow off the diagonal represent pseudo-linkage between chromosomes. **a**, recombination in the population ‘Goslin’ x ‘HiFi’, where Goslin carries a 1C-A translocation (like Sang) and HiFi is a non-carrier. The result is that a large proportion of 1C shows pseudo linkage to the part of 1A where the translocation is present (large yellow rectangle in the intersection of 1A and 1C). **b**, both parents of the population (‘TX07CS‐1948’ x ‘Hidalgo’) carry the same translocation as Sang thus no pseudo linkage is observed. The 1C-1A translocation is associated with adaptation-related traits^20^, with pseudo linkage limiting the possibility of recombining different traits affected by genes in the translocated region in crosses between translocated and non-translocated germplasm.

Previous molecular marker studies using oat mapping and breeding populations have provided independent evidence for frequent translocations among oat subgenomes^18,19^. Using the oat genome to reanalyse the data (Extended Data Fig. 6), we observed inter-chromosomal pseudo-linkage in a population that segregates for the 1C translocation on 1A. Such pseudo-linkage has been implicated in the propensity for cold hardiness to remain associated with non-carriers of this translocation^20^. An accompanying study^21^ details similar associated opportunities and barriers in genomic breeding strategies. The mosaic nature of the oat genome may be associated with the apparent lack of an orthologue of *TaZIP4-B2* (located within the *Ph1* locus), which in bread wheat stabilizes the genome structure during meiosis and suppresses crossovers between homoeologues^22–24^ (Extended Data Fig. 5b and Supplementary Figs. 14 and 15). In contrast to wheat^25^, interploidy crosses and alien introgressions have been extremely challenging in *Avena*^26^, suggesting that incompatible genome architecture is an additional barrier preventing genetic gains in oat.

## Oat subgenome expression is balanced

**Extended Data Fig. 7:**
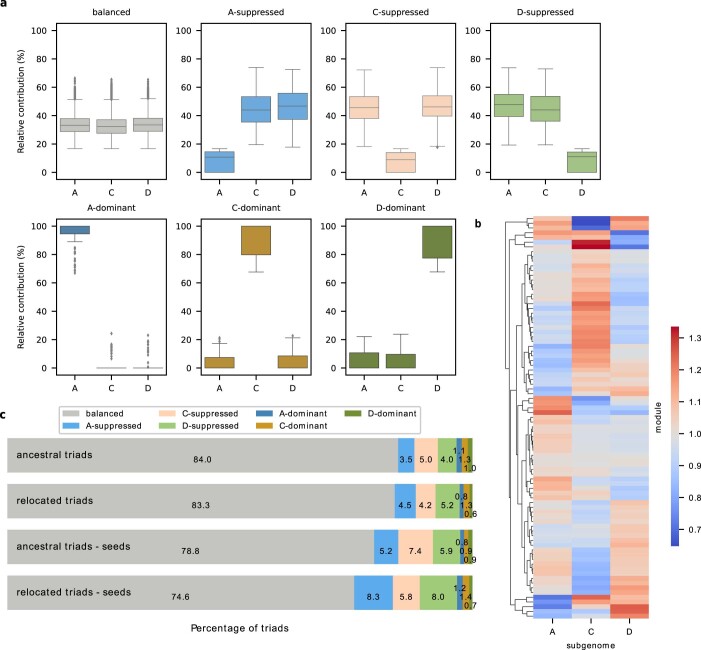
Gene expression analysis in the hexaploid oat genome. **a**, Box plots representation of the relative contribution of each subgenome based on triad assignment to the seven categories defined, balanced n = 6,308, A-suppressed n = 263, C-suppressed n = 378, D-suppressed n = 303, A-dominant n = 83, C-dominant n = 98, D-dominant n = 74, n=number of triads. Values from the first to third quartiles are shown within the boxplots boxes (inter-quartile range) with the median represented by the middle line. The upper and lower whiskers extend from the edge to the largest and smallest value of the edge but no further than 1.5 × the  inter-quartile range, the data beyond the end of the whiskers are outliers plotted individually. **b**, Heatmap representation of WGCNA modules showing the relation between the expected representation of each subgenome in the module based on the overall number of genes per subgenome and the observed one. > 1: higher than expected, < 1 lower than expected. 1 = as expected. **c**, Gene expression patterns in ancestral triads versus triads with genes positioned in translocated regions across all samples (upper two panels) and in seed tissue (lower two panels).

After polyploidization, sub- and neofunctionalization and gene loss modify the gene content in the new species^27,28^. Systematic differences in subgenome/homoeologue gene expression (homoeologue expression bias^29^) may also be prevalent. In fact, quantitative variation for many agronomic traits may reflect genetic interactions between homoeologues such as functional redundancy (buffering) or dominant phenotypes attributed to one homoeologue^30^. To investigate homoeologue expression bias in hexaploid oat, we defined 7,726 homoeologous gene triads with a 1:1:1 correspondence across the three oat subgenomes (Supplementary Table 11), referred to as ancestral triads. Average expression values across transcriptome samples from six tissues showed that C-subgenome genes were slightly less expressed (32.32%) than those in the D (33.53%) and A (33.76%) subgenomes (Kruskal–Wallis, *P* = 0.054). We considered six homoeologous expression categories^31^ and found that most ancestral triads (84.1%) showed balanced expression, 3.4% showed single-homoeologue dominance and 12.6% showed single-homoeologue suppression. The relative contributions of the different categories (Extended Data Fig. 7a) indicated no major overall bias for one of the subgenomes (Fig. 2d). A co-expression network approach revealed that genes from the C subgenome were found in divergent expression modules more frequently than their A- and D-subgenome homoeologues (*χ*^2^ test, *P* = 2.085 × 10^−6^; Extended Data Fig. 7b and Supplementary Table 12).

In another 1,508 triad gene clusters containing at least one member positioned in a translocated region (relocated triads; Supplementary Table 13), the overall expression patterns were similar to those of the ancestral triads (Extended Data Fig. 7c). The C-suppressed category was slightly larger (5.1%) in the ancestral triads compared with the A-suppressed (3.5%) or D-suppressed (4.1%) triads, but the subgenome suppression patterns were reversed (4.5% A, 4.2% C and 5.2% D) in the relocated triads (*χ*^2^ test, *P* = 0.019; Extended Data Fig. 7c). Our results indicate that translocations and rearrangements in the oat genome may affect global and homoeologous gene expression patterns. Understanding how homoeologues interact to influence gene expression and identifying functional single-copy genes showing non-balanced expression will inform crop improvement in oats.

## Soluble fibre-related gene families

**Fig. 3:**
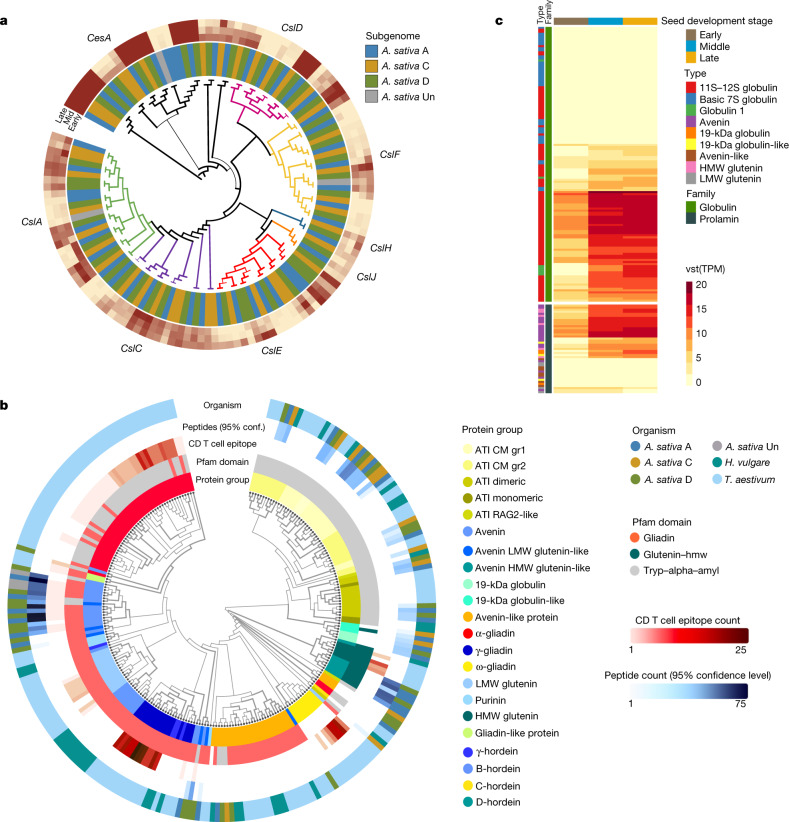
Analysis of cellulose synthase and seed storage protein gene families in *A*. *sativa*. **a**, Phylogeny of the cellulose synthase protein superfamily in *A*. *sativa* highlighting the eight subfamilies. Outer tracks represent the variance stabilizing-transformed transcripts per million (TPM) values determined for early, middle and late seed-development stages. The TPM level correlates with the intensity of burgundy colouring; the branch thickness corresponds to the bootstrap values and increases with higher bootstrap. **b**, Schematic representation and phylogeny of cereal storage proteins. The protein types used for the analysis were: wheat gliadins, glutenins, avenin-like proteins and ATIs^7^; barley hordeins, avenin-like proteins and ATIs^53^; and oat avenins, HMW glutenins, avenin-like proteins and ATIs identified in this study. Pfam domains and the identified protein groups are highlighted in separate layers. Epitopes used for the analysis included coeliac disease (CD)-associated T cell epitopes^47^. The numbers of T cell epitopes are labelled in the red colour scale. The number of peptides identified at the 95% confidence level are labelled in the blue colour scale; the branch thickness corresponds to bootstrap values and increases with higher bootstrap. LMW, low molecular weight. **c**, Expression of oat prolamin and globulin genes in three stages of seed development. The variance stabilizing transformed (vst) TPM levels correlate with the intensity of yellow to red colouring.

**Extended Data Fig. 8:**
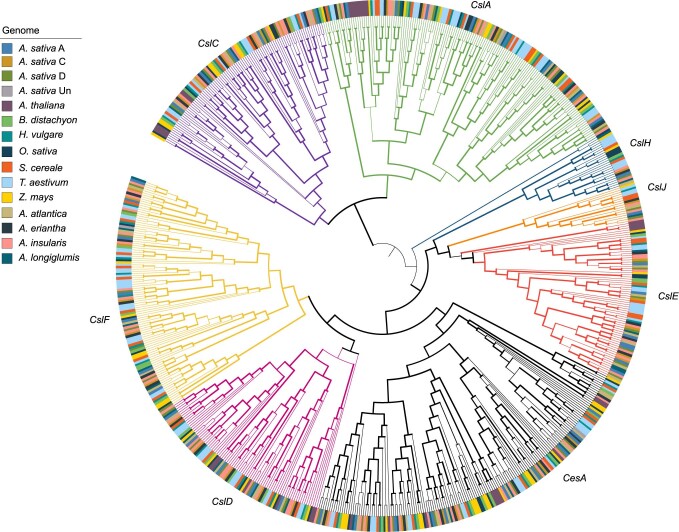
Phylogeny of the CesA-subfamily of cellulose synthase genes and the Cellulose synthase-like subfamilies. *CslA, C, D, E, F, H*, and *J* in 11 different species including oat. Tree branches for different subfamilies are shown in different colours. Branch thickness corresponds to bootstrap values and increases with higher bootstrap.

Mixed-linkage β-glucans are soluble fibres present at high levels in oat endosperm cell walls (3.8–6.1 g per 100 g dry weight) that reduce blood cholesterol and post-meal glycaemic responses^3,4^. The cellulose synthase-like gene *CslF6* is central for β-glucan biosynthesis in cereals^32,33^. We catalogued the cellulose synthase (*GT2*) and callose synthase (*GT48*) families of glycosyltransferases to identify the genetic foundation underlying oat β-glucan biosynthesis. The hexaploid oat genome encodes 134 members of the cellulose synthase gene superfamily (Fig. 3a), representing the cellulose synthase (*CesA*) subfamily (Supplementary Fig. 16) and seven cellulose synthase-like subfamilies, including *CslA*, *CslC*, *CslD*, *CslE*, *CslF*, *CslH* and *CslJ*. The *GT48* family comprised 28 members (Supplementary Fig. 17). Genes within the *CesA* and *CslF* subfamilies were most highly expressed over multiple stages of seed development (Fig. 3a and Supplementary Fig. 18). Investigation of differentially expressed genes between stages indicated specific roles for particular subfamilies such as *CslE* and *CslF* (including the C-subgenome copy of *CslF6*), which were upregulated in late stages of seed development (Supplementary Fig. 19), as shown in barley^34^. Compared with other grasses, the oat cellulose synthase superfamily showed no significant expansions apart from duplication events in the *CesA*, *CslC*, *CslE* and *CslJ* subfamilies (Supplementary Fig. 20 and Extended Data Fig. 8). These findings suggest that the high content and quality of β-glucan in oat are not driven by major differences in the copy number of cellulose synthase superfamily genes relative to other grasses but rather by allelic variation and transcription factors, as previously reported^34^.

## Oat storage proteins and human health

Oat globulins constitute 75–80% of grain protein content, with prolamins (avenins) accounting for approximately 10–15%. Prolamin superfamily members trigger coeliac disease, food allergies and baker’s asthma^35^. We identified genes encoding 25 avenins, 6 high-molecular-weight glutenins (HMW-GS) and 61 genes representing α-amylase/trypsin inhibitors (ATIs) and other prolamin superfamily members related to protein accumulation and immunogenicity (Supplementary Table 14). Hexaploid oat has avenin loci on chromosomes 1D, 3D and 7A; seed storage globulin loci (135 genes) on chromosomes 1A, 1D, 3D, 7A, 4A and 4D; and no storage protein loci mapping to the C subgenome (Fig. 3b and Supplementary Table 14).

**Extended Data Fig. 9:**
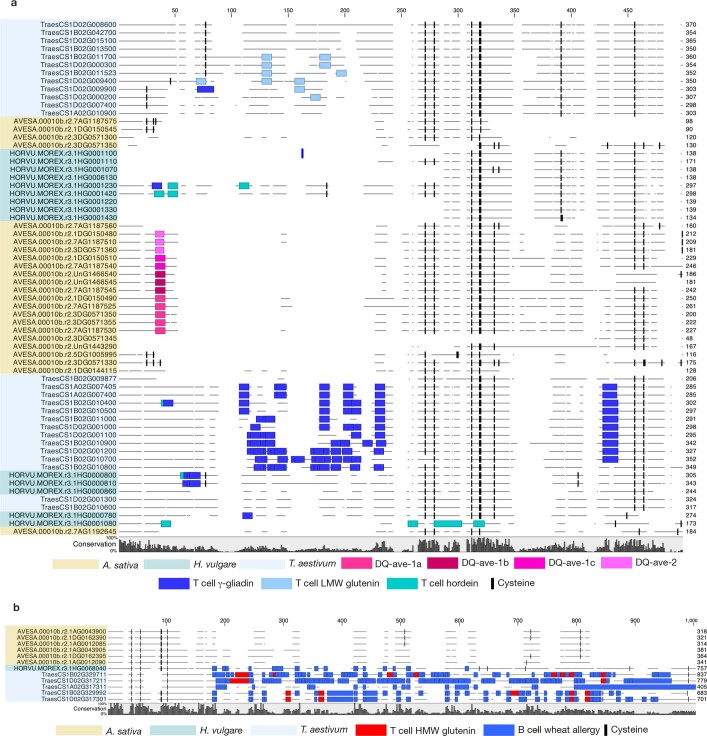
Comparative analysis of coeliac disease and allergy related epitopes in oat, wheat and barley. **a**, Protein sequence alignment of avenins with γ-gliadins, B-hordeins, and LMW glutenins shows the conserved position of cysteine residues (black bars) and differences in the T cell epitope prevalence and variability. Blue bars represent T cell epitopes characteristic at γ-gliadins, green bars indicate epitopes characteristic at LMW glutenins, pink bars show positions of avenin-specific epitopes. Colour code on the left side indicates the species: oat (highlighted in pale yellow); wheat (highlighted in pale blue); and barley (highlighted in jagged ice). **b**, Sequence alignment of HMW glutenins show the conserved position of cysteine residues in the N- and C-terminal regions in oat and wheat HMW glutenins and barley D-hordein and shows the complete absence of coeliac disease (highlighted in red) and wheat allergy-related epitopes (shown as blue bars) in the sequences.

Unlike that of wheat, the oat genome harboured no α*-* or ω*-*gliadin genes, and the identified avenins co-clustered with γ-gliadins, low-molecular-weight glutenins and B-hordeins (Fig. 3b). We detected four complete, highly conserved oat HMW-GS gene models as two distinct loci on 1A and one locus pair on 1D, with no HMW-GS genes mapping to 1C. We identified a prolamin type, the 19-kDa globulin-like proteins, with an unknown function that is distinct from the avenins yet shares sequence similarity with HMW-GS and 19-kDa globulins (Fig. 3b). The predicted oat HMW-GS and avenins were highly conserved in their Pfam domains (Fig. 3b) and cysteine patterns (Extended Data Fig. 9). Glutamine- and proline-rich repetitive peptides were fewer in these oat proteins, making them shorter than those in wheat or barley (Extended Data Fig. 9).

We detected transcripts for most of the avenin genes, which showed gene expression patterns that aligned with their wheat orthologues, with increased transcript levels from the middle phase of seed development^36^ (Fig. 3c), and protein levels by using liquid chromatography with tandem mass spectrometry (Fig. 3b). We identified inactive genes and pseudogenes among avenin-encoding genes (Fig. 3b, c and Supplementary Table 14) in a similar proportion as in wheat γ-gliadins^37,38^. This indicates a lower level of gene expansion and pseudogenization compared with the highly immunogenic wheat ɑ-gliadin genes^39^. Moreover, the expression of 11S globulin genes initiated early in seed development and was higher than that of the avenin genes (Fig. 3c). Discovery proteomics detected thirty-six distinct 11S globulins, five globulin-1 proteins and two 7S globulins, with an average of 83% protein sequence coverage at a 1% false discovery rate.

**Extended Data Fig. 10:**
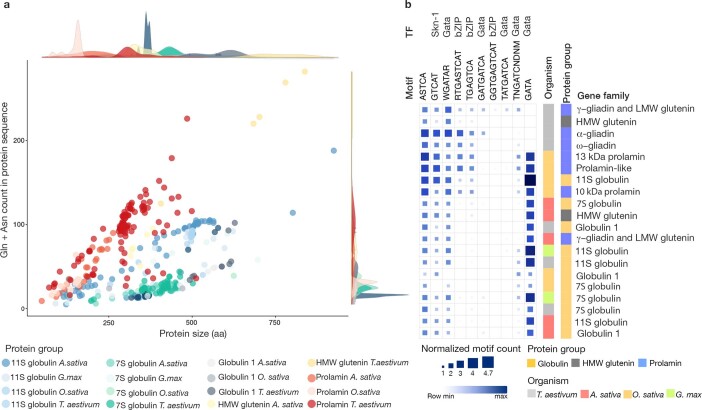
Comparative analysis of nitrogen storing capacity and transcription factor binding site motifs. **a**, Size distribution of prolamin and globulin protein sequences in amino acid residues (aa) identified in the genomes of hexaploid oat cultivar Sang, the IWGSC v1 wheat reference genome, *Oryza sativa* Japonica IRGSP-1.0 rice genome and soybean reference genome, *Glycine max* v2.1 in relation to their nitrogen storing capacity, measured as a sum of asparagine and glutamine content in their sequence. **b**, Comparison of nitrogen-responsive transcription factor binding site patterns in oat, wheat and rice prolamins and oat, wheat, rice and soybean globulins. Consensus motifs identified in the 0 to −500 region and the related transcription factor families are labelled in each column; total numbers of detected motifs normalised by the number of gene models are indicated in each row.

The oat avenins and globulins showed opposite trends compared with their wheat orthologues in gene copy number, protein length and enrichment in glutamine and asparagine residues that serve as a nitrogen storage sink (Extended Data Fig. 10a). Together with pronounced differences in transcription factor-binding sites specific to the nitrate response (Extended Data Fig. 10b and Supplementary Table 15), this may contribute to the primary role of oat globulins in nitrogen storage. These results confirm that the genomic organization, sequence characteristics and expression patterns of oat storage proteins share more similarities with rice and dicotyledonous plants than with wheat and other gluten-rich cereals^40,41^.

We mapped previously reported coeliac disease-associated T cell epitopes to the predicted oat avenin proteins and compared them with the T cell epitope patterns of wheat and barley prolamins^42^. The results showed that only a subset of encoded avenin proteins contain coeliac disease-associated immune-reactive regions compared with the high prevalence found in wheat or barley (Fig. 3b). Taken together, the low copy number of genes encoding coeliac disease epitopes, low frequency of detected T cell epitopes in the protein sequence, low occurrence of other highly immunogenic proteins, proportion of avenins within total oat protein and relative immunogenicity of avenin epitopes^43^ all support the inclusion of oats in gluten-free diets^35^.

## Single-gene mapping of a wax mutant

**Fig. 4:**
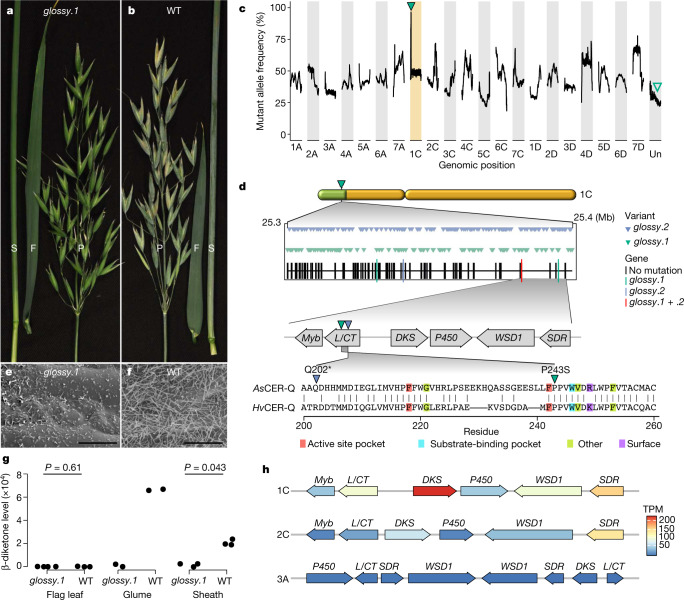
Single-gene mapping of an epicuticular wax mutant. **a**, **b**, Epicuticular wax phenotypes of the *glossy*.*1* mutant (**a**) and the glaucous parental cultivar (**b**) at the early grain filling stage. S, sheath; F, flag leaf; P, panicle. **c**, Sliding window of allele frequency for variants unique to *glossy*.*1*. A window of 100 variants (total allelic depth ≥ 30) was used. Green triangle, chromosomal region shared by the *glossy.1* pool; empty triangle, location of the contig with the candidate gene in the assembly. Hi-C data anchor the contig to the 1C peak. **d**, Mapping of the *glossy*.*1* locus. Top, genes and variants (total allelic depth ≥ 30) at the 1C peak. Middle, the candidate gene (Gene-ID: *AVESA*.*00010b*.*r2*.*UnG1403470*) encoding a lipase/carboxyltransferase (L/CT), indicated by the red vertical bar, is located in the putative biosynthetic gene cluster orthologous to the barley *Cer*-*cqu* cluster. The genes encoding diketone synthase (DKS) and L/CT are orthologous to the barley *Cer*-*c* and *Cer*-*q* genes, respectively (Supplementary Figs. 22 and 28). Green triangle, *glossy*.*1* mutation; blue triangle, *glossy.2* mutation. Bottom, alignment of *Hv*CER-Q and *As*CER-Q. Known deleterious single-amino acid substitutions from barley^49^ are indicated. **e**, **f**, Scanning electron micrographs of the glume cuticle surface in *glossy*.*1* (**e**) and the glaucous parental cultivar (**f**) at ×4,000 magnification; scale bars, 10 µm. **g**, Hentriacontane-14,16-dione is the major metabolite not detected in *glossy*.*1* (two-sided Welch *t*-test, *P* values adjusted using Benjamini–Hochberg procedure; glaucous flag leaf, *n* = 4; *glossy* flag leaf, *n* = 3; sheath, *n* = 3; glume, *n* = 2). **h**, Homoeologous gene clusters on chromosomes 1C, 3A and 2C. Genes are coloured according to the mean TPM value (four biological replicates) in glaucous glumes. *Myb*, Myb factor; *P450*, cytochrome P450; *WSD1*, wax ester synthase/diacylglycerol acyltransferase 1; *SDR*, short-chain dehydrogenase/reductase.

To demonstrate how an annotated reference genome enables greater use of resources such as TILLING populations^44,45^, we mapped the causal mutation in the epicuticular wax mutant *glossy*.*1* (Fig. 4a, b). Epicuticular waxes have a role in biotic and abiotic stress resistance^46,47^ and provide an important target for oat breeding. We identified homozygous polymorphisms unique to the mutant, which mapped to chromosome 1C (Fig. 4c and Supplementary Fig. 21), and identified a single gene annotated as an α/β-hydrolase (*AVESA*.*00010b*.*r2*.*UnG1403470*) as a likely candidate that is orthologous to barley *Cer-q* (*HORVU*.*MOREX*.*r3*.*2HG0097460*) (Supplementary Fig. 22). An independent mutant line (*glossy*.*2*) exhibited the same glossy phenotype (Fig. 4d, Supplementary Fig. 23 and Supplementary Table 16). Barley *Cer-q* mutants^48^ are deficient in the same β-diketone (hentriacontane-14,16-dione) and wax tubules that are absent in the *glossy* mutants (Fig. 4e–g and Supplementary Figs. 24–26). The scaffold containing the candidate gene was localized to the region of chromosome 1C (Fig. 4c and Supplementary Table 17). The presumed *glossy*.*1* mutation introduced a P243S substitution in the encoded protein adjacent to a deleterious F219L substitution known to inactivate barley CER-Q^49^ (Fig. 4d and Supplementary Fig. 27). We identified gene clusters on oat chromosomes 1C, 2C and 3A and in wild *Avena* species (Supplementary Figs. 22 and 28–33) that are homologous to the barley *Cer*-*cqu* cluster^49,50^. We also noted genes encoding proteins with similarity to *Arabidopsis* wax ester synthase/diacylglycerol acyltransferase 1 (*WSD1*), a Myb-domain transcription factor and a short-chain dehydrogenase/reductase (*SDR*) protein near the *Cer-cqu* homologues in the *Avena* genomes. All genes from the 1C cluster except *SDR* were expressed at levels 3–6 times higher than those of the 3A cluster, with very low expression from 2C cluster genes and with no differential expression between the *glossy* and glaucous glume tissue (two-sided Wald test, null hypothesis logarithmic fold change = 0, adjusted *P* < 0.01; Fig. 4h and Supplementary Fig. 34). Together, these results suggest that *AVESA*.*00010b*.*r2*.*UnG1403470* is the oat *Cer-q* gene. The reference genome thus facilitated a major advance in understanding β-diketone biosynthesis in oat and can help breeders manipulate tissue-specific epicuticular wax composition in future oat cultivars adapted for hotter climates.

## Discussion

In summary, this fully annotated hexaploid oat reference genome lays the foundation for advances in oat breeding and basic oat biology and for the ongoing pan-genome project. With the chromosome rearrangements in a typical spring oat cultivar now delineated, breeders and researchers will have access to a resource equal in calibre to Triticeae genomes, which may help them to overcome the breeding barriers and segregation anomalies described in numerous mapping studies. Using the reference genome to map genes associated with agronomic and human nutrition-related traits is a viable approach for precisely adapting oat varieties. Known quantitative trait loci can be anchored to the Sang reference, and the transcriptome atlas co-expression networks can be leveraged to identify candidate genes in the vicinity of specific quantitative trait loci. Modern breeding strategies such as genome editing and gene pyramiding can now more easily be applied in oat to develop varieties that meet the increasing global demand for oat-derived products. Our proteogenomic investigation of oat storage proteins confirms qualitative and quantitative differences in the expression of proteins compared with the more abundant and immunogenic sequences in wheat, barley and rye, which supports the safety of oats in gluten-free diets. The detailed genome annotation and case studies presented here provide examples of the myriad possibilities for the discovery and exploitation of functional genetic mechanisms in oat.

### Reporting summary

Further information on research design is available in the Nature Research Reporting Summary linked to this paper.

## Online content

Any methods, additional references, Nature Research reporting summaries, source data, extended data, supplementary information, acknowledgements, peer review information; details of author contributions and competing interests; and statements of data and code availability are available at 10.1038/s41586-022-04732-y.

## Supplementary information

Supplementary Information This file contains Supplementary Figs. 1–36, Supplementary Tables 1, 4–6, 8–10 and 21, Supplementary Methods and Supplementary References. The display items include 36 figures and 8 tables that support the validity of the genome assemblies, genome organization, chromosome rearrangement, gene families and expression analyses and single-gene mapping of the oat mutant.

Reporting Summary

Supplementary Tables This file contains Supplementary Tables 2, 3, 7, 11–20 and 22, which support the genome annotation, ancestral and relocated triads, asymmetric modules, storage proteins and associated transcription factor-binding sites, single-gene mapping, assembly statistics of ancestral *Avena* genomes, metadata for datasets and samples and accession IDs used in the gene family analyses.

## Data Availability

The raw sequence data used for de novo whole-genome assembly are available from the European Nucleotide Archive (ENA) under accession number PRJEB44810 (*A*. *sativa* cv. Sang) and from the Sequence Read Archive under accession numbers PRJNA727490 (*A*. *insularis* BYU209) and PRJNA726919 (*A*. *longiglumis* CN58138). Chromosome conformation capture (Hi-C) sequencing data are available from ENA under accession numbers PRJEB43668 (*A*. *sativa* cv. Sang), PRJEB43670 (*A*. *insularis* BYU209) and PRJEB43669 (*A*. *longiglumis* CN58138). Chromosome-scale sequence assemblies (pseudomolecules) are available from ENA under accession numbers PRJEB44810 (*A*. *sativa* cv. Sang), PRJEB45088 (*A*. *insularis* BYU209) and PRJEB45087 (*A*. *longiglumis* CN58138). The raw RNA-seq and genome-sequencing data generated in this study are available under ENA accession number PRJEB46365. Pseudomolecules, annotation data and analysis results are available at the Plant Genomics and Phenomics Research Data Repository at 10.5447/ipk/2022/2. The DOI was registered using e!DAL (https://edal.ipk-gatersleben.de/). Pseudomolecules, annotation data and associated analyses for *A. sativa* cv. Sang, *A*. *longiglumis* and *A. insularis* are also available from GrainGenes^54^: Sang genome browser, https://wheat.pw.usda.gov/jb/?data=/ggds/oat-sang; Sang data download, https://wheat.pw.usda.gov/GG3/content/avena-sang-download; *A*. *longiglumis* genome browser, https://wheat.pw.usda.gov/jb/?data=/ggds/oat-longiglumis; *A*. *longiglumis* data download, https://wheat.pw.usda.gov/GG3/content/avena-longiglumis-download; *A*. *insularis* genome browser, https://wheat.pw.usda.gov/jb/?data=/ggds/oat-insularis; *A*. *insularis* data download, https://wheat.pw.usda.gov/GG3/content/avena-insularis-download. The mass spectrometry proteomics data and ProteinPilot search result files have been deposited to MassIVE (https://massive.ucsd.edu) under accession number MSV000088727. The publicly available OT3098 oat genome data were generated by PepsiCo and Corteva Agriscience. This dataset (annotation version 2) has been obtained and is available from GrainGenes at https://wheat.pw.usda.gov/GG3/content/pepsico-ot3098-hexaploid-oat-version-2-genome-assembly-release-collaboration-graingenes. Databases used in this study included PTREP release 19, Uniref download 2019-09-03, Pfam download 2019-09-03, Swiss-Prot, TAIR, TrEMBL, REdat_9.9_Poaceae section of the PGSB transposon library, Immune Epitope Database and Analysis Resource (https://www.iedb.org), PLACE and PlantCare promoter motif databases and pfam2GO.

## Code availability

All relevant code developed in this study was deposited in the GitHub node at https://github.com/PGSB-HMGU/oatkmers.
