# Supplementary material for: The mosaic oat genome gives insights into a uniquely healthy cereal crop
Source: Nature. 2022 May 18;606(7912):113–9. doi: 10.1038/s41586-022-04732-y (PMC9159951; doi:10.1038/s41586-022-04732-y)
Supplement: Supplementary file 1 — This file contains Supplementary Figs. 1–36, Supplementary Tables 1, 4–6, 8–10 and 21, Supplementary Methods and Supplementary References. The display items include 36 figures and 8 tables that support the validity of the genome assemblies, genome organization, chromosome rearrangement, gene families and expression analyses and single-gene mapping of the oat mutant. [file 41586_2022_4732_MOESM1_ESM.pdf]

nature portfolio

https://doi.org/10.1038/s41586-022-04732-y

Supplementary information

The mosaic oat genome gives insights into a uniquely healthy cereal crop

In the format provided by the authors and unedited

Supplementary Figures

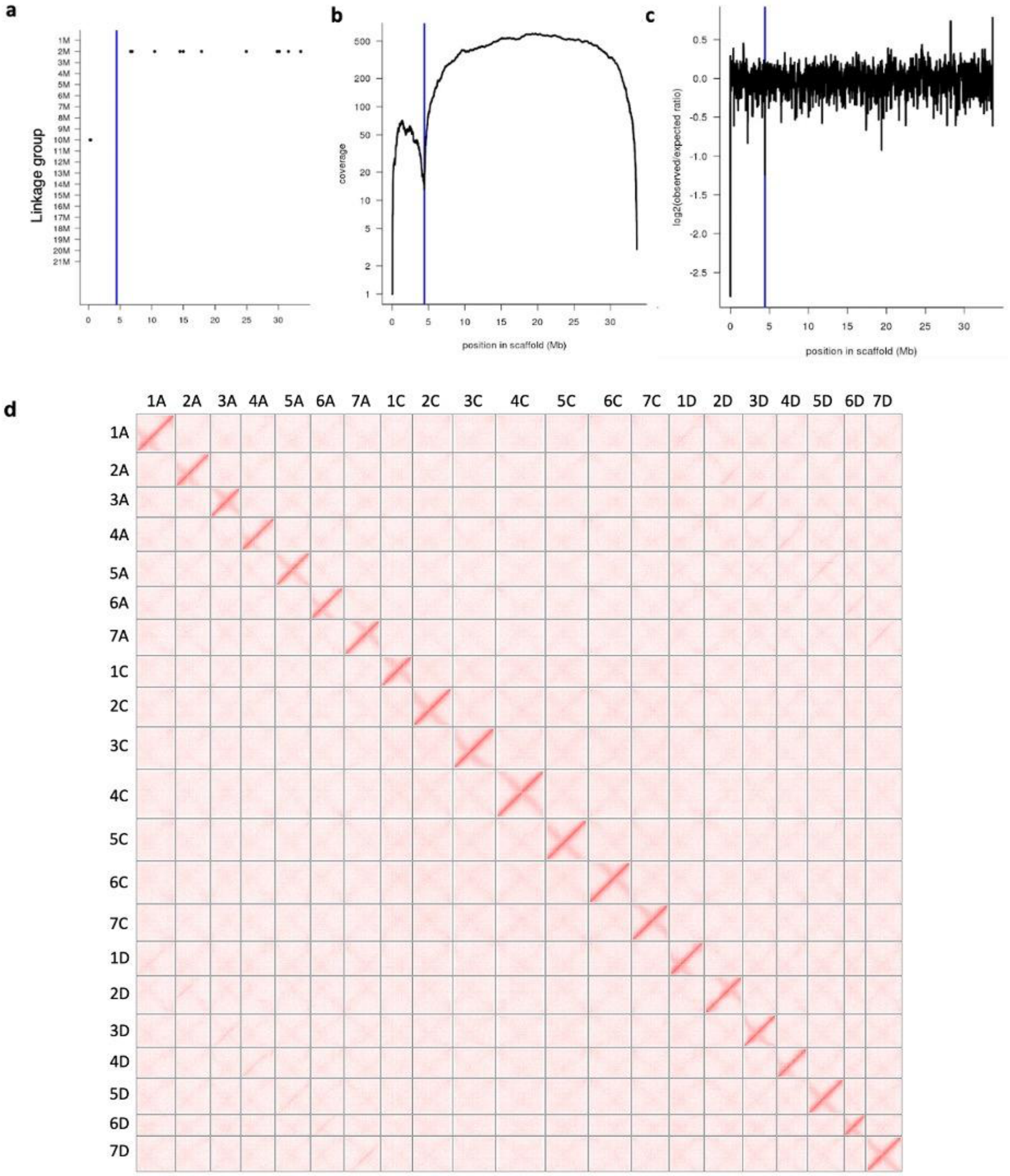

Supplementary Figure 1. Validation of hexaploid oat pseudomolecule assembly. Multiple types of evidence were used to detect and correct mis-joins in sequence scaffolds: concordance with a consensus genetic map **a**, physical coverage with HiC-data **b,** and physical coverage with 10X Genomics linked reads **c,** Breakpoints are often co-located with drops in 10X and/or Hi-C coverage. **d**, Hi-C contact matrix of *A. sativa* cv. Sang.

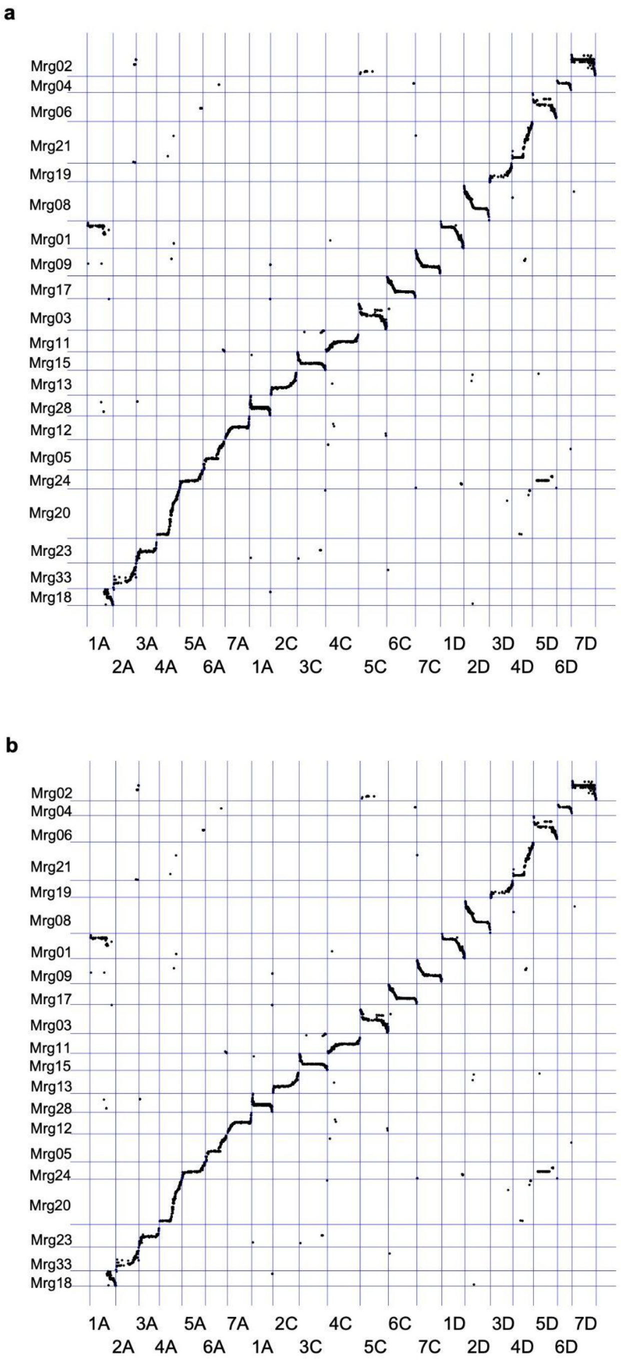

Supplementary Figure 2. Colinearity of the pseudomolecules *A. sativa* cv Sang. **a**, and OT3098 **b**, with the consensus genetic map of Bekele et al. (2018)[10].

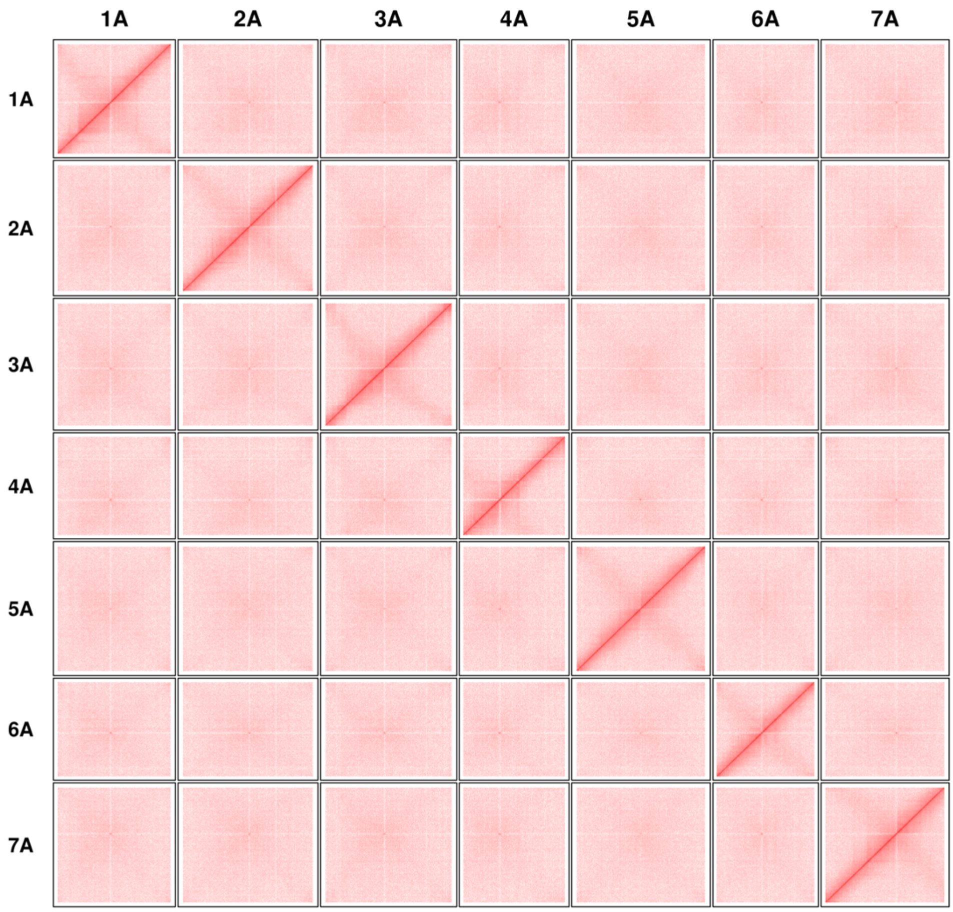

Supplementary Figure 3. Hi-C contact matrix of *A. longiglumis* CN58138.

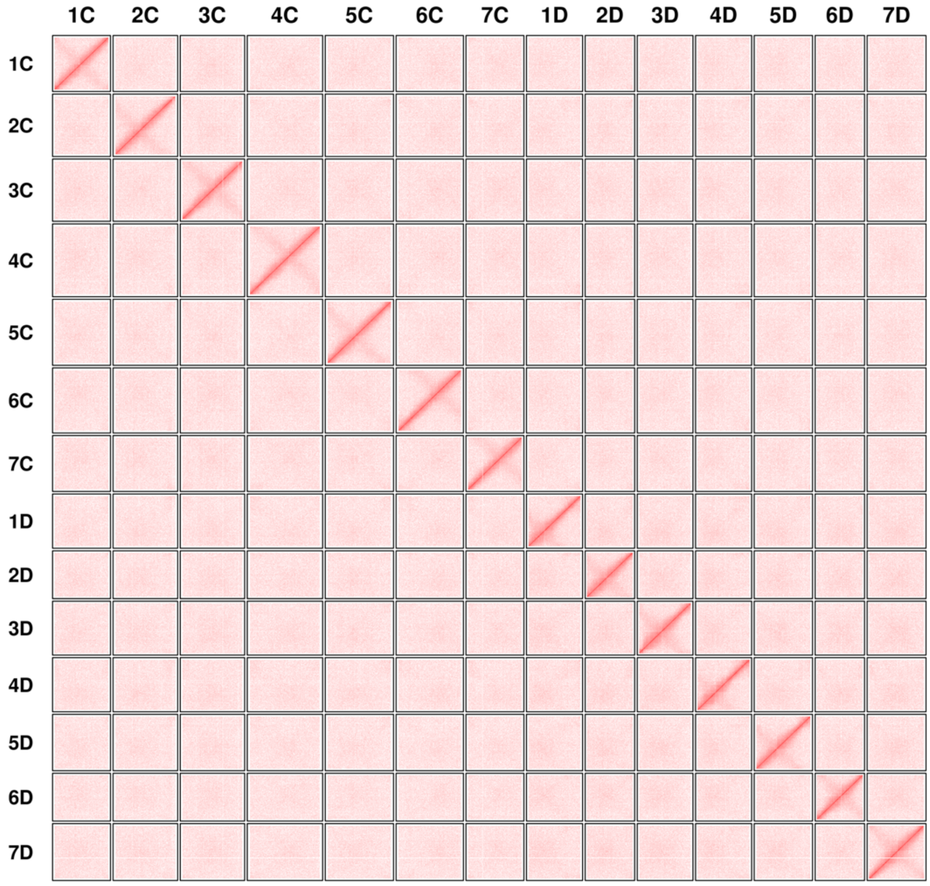

Supplementary Figure 4. Hi-C contact matrix of *A. insularis* BYU209.

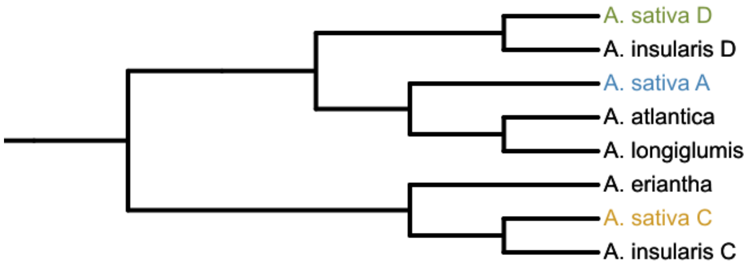

Supplementary Figure 5. Illustration of the genetic relationships of *Avena sativa* subgenomes (A, C, D) with its presumptive diploid genome (A) and tetraploid ancestor subgenomes (C, D). Dendrogram constructed from orthologous gene clustering.

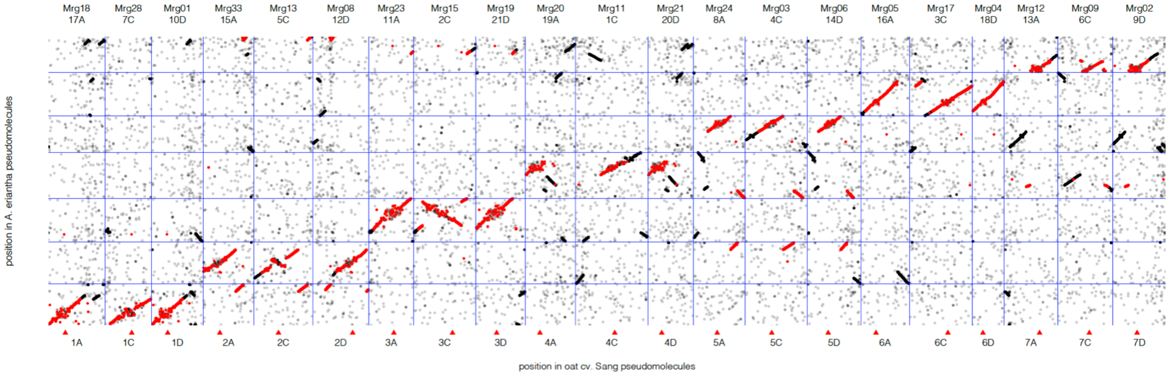

Supplementary Figure 6. Gene-based collinearity of *A. sativa* to *A. eriantha*. Each data point is an aligned gene. Genes in core regions are shown in red. The phylogenetically informed nomenclature is used on the bottom x-axis. The top axis shows the chromosome names according to Bekele et al. (2018)[10] (Mrg groups) and according to Sanz et al. (2010)[13]. Centromere positions in oat are indicated by red triangles (x axis) and red diamonds (y axis), respectively. Note that *A. sativa* genes (query) were aligned to *A. eriantha* (reference). Hence, genes with A and D origins map to their orthologues in the C genome.

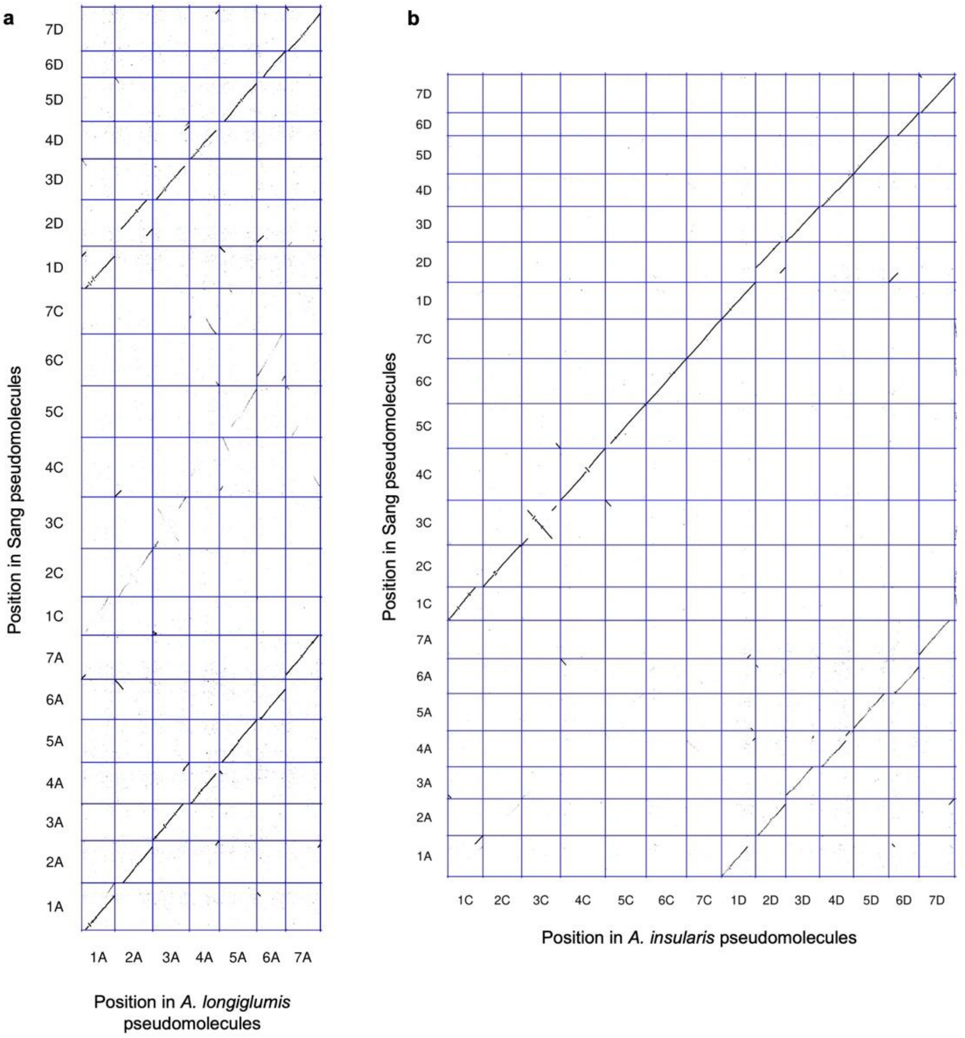

Supplementary Figure 7. **Validation of the revised oat chromosome nomenclature. a,** Alignments between *A. sativa* cultivar Sang and *A. longiglumis.* **b,** Alignments between *A. sativa* cultivar Sang and *A. insularis*.

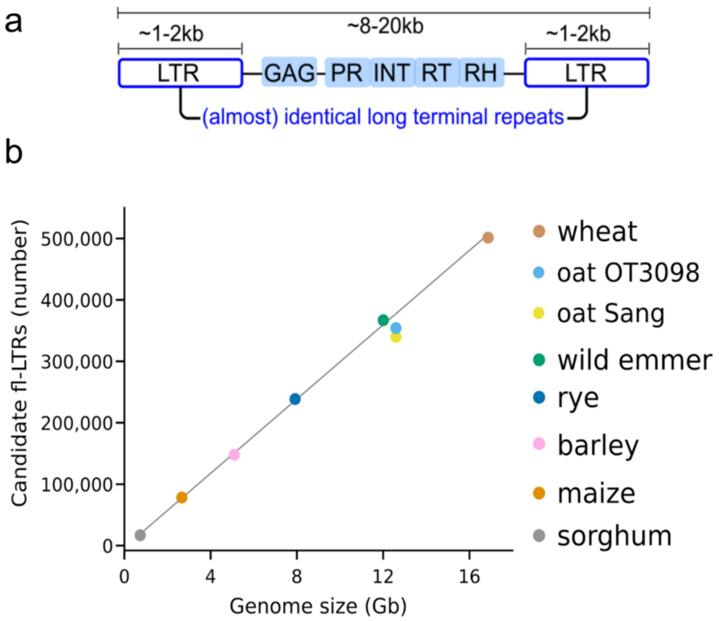

Supplementary Figure 8. Repeat element score (REsco): full length LTR-retrotransposons as proxy for assembly quality. **a,** Due to their structural properties of two highly similar or even identical long terminal repeats, full length copies of LTR-retrotransposons (fl-LTR) are difficult to assemble and can thus be used as a metric for the correct reconstruction of the repetitive space in an assembly. **b,** Calibrated by six reference genome assemblies[7,9,16,55–57], among them two long read assemblies (maize and barley) the oat cv. Sang assembly reaches a REsco score of 90%, the OT3098 assembly 94%.

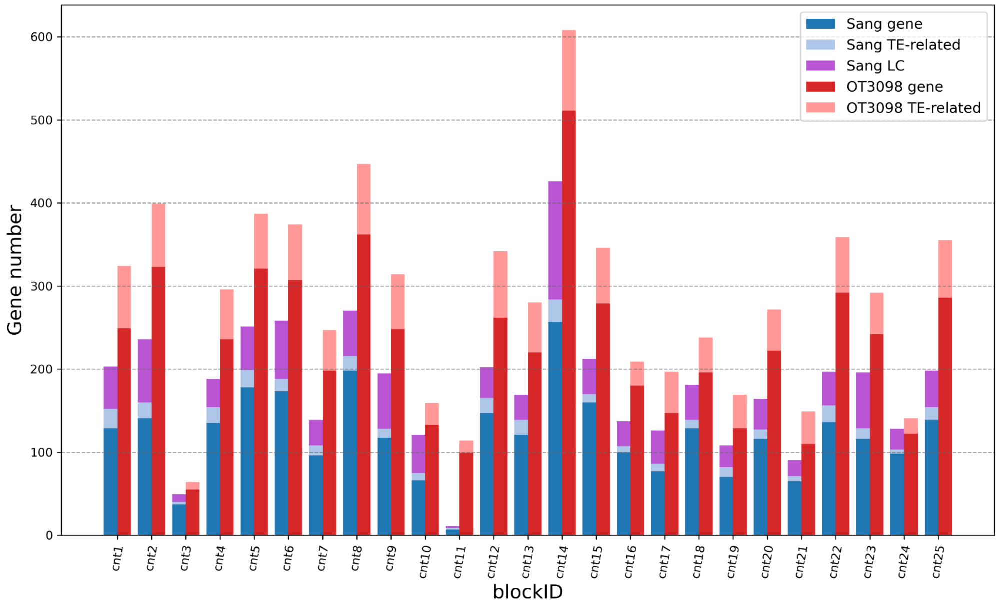

Supplementary Figure 9. Estimated level of gene and TE-related gene count differences between the Sang and OT3098 assemblies in regions of reduced/differing gene density (X-axis shows the blockID; defined in Supplementary Table 7). Genes observed in these regions were dissected for high-confidence (HC) genes (all published OT3098 gene predictions belong in this category), low-confidence (LC) genes (Sang only) and TE-related gene models (both genome assemblies). As expected for a long-read assembly, an overall higher amount of gene predictions in OT3098 was found in these mainly low-complexity regions. In comparison to Sang, OT3098 comprises proportionally more gene predictions with PFAM domains that are characteristic for transposable elements in these regions. The elevated number of transposon-derived genes in OT3098, as well as the orthologous Sang low-confidence (LC) genes identified in addition, only partially account for the observed lower number of gene predictions in the short-read assembly of Sang in these regions. We therefore report the borders of the Sang genomic regions with reduced gene density in Supplementary Table 7.

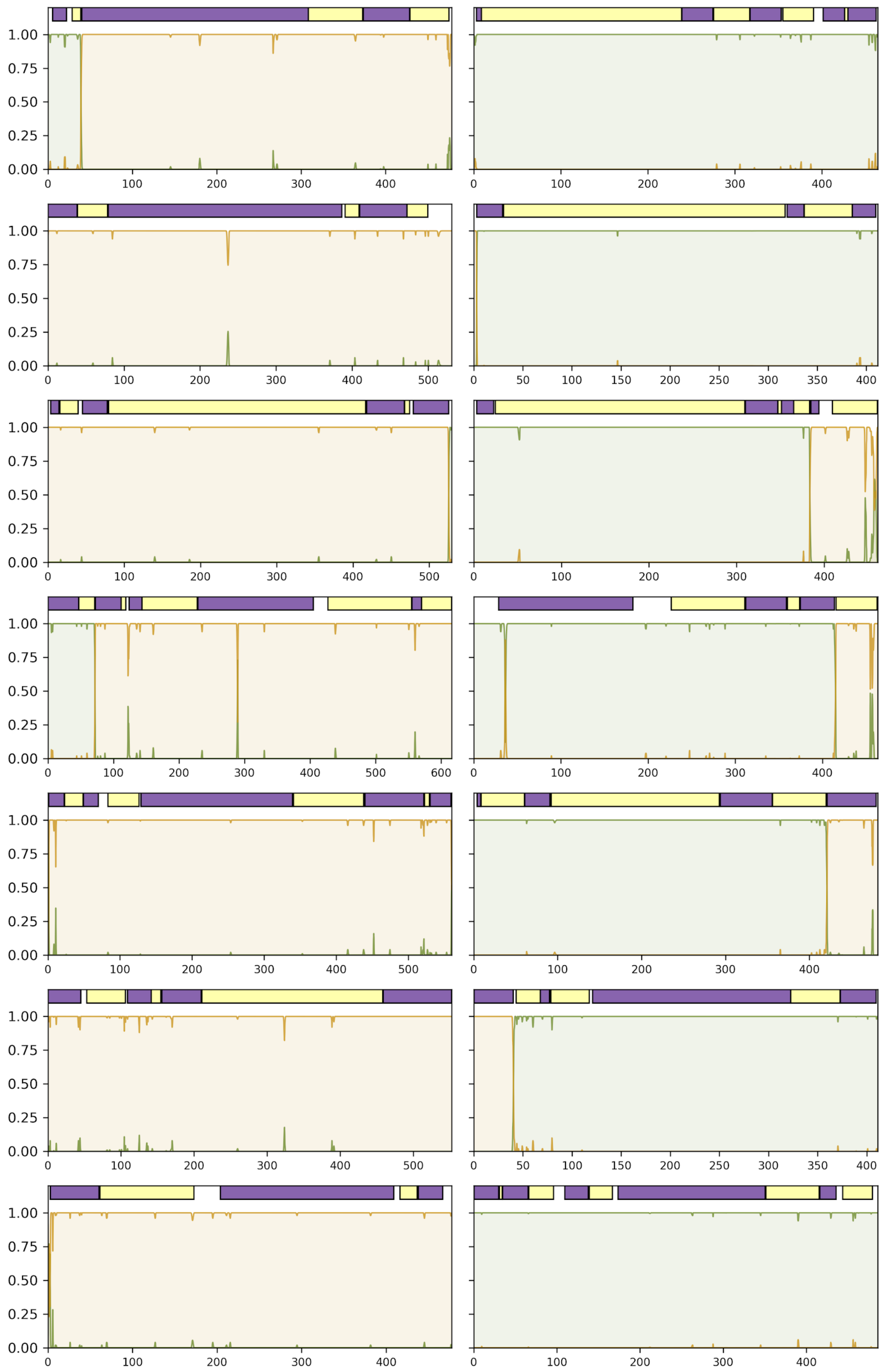

Supplementary Figure 10. Subgenome-specific kmers and syntenic blocks in *A. insularis*. Probabilities of C- and D-subgenome classification by subgenomic-kmers are shown. Y-axis: kmer-probability, x-axis: chromosomal position. Top row in each subplot displays identified syntenic blocks shown in alternative colours to emphasise block borders (green: D, gold: C). From top to bottom, chromosomes 1 to 7 for subgenomes C (left) and D (right).

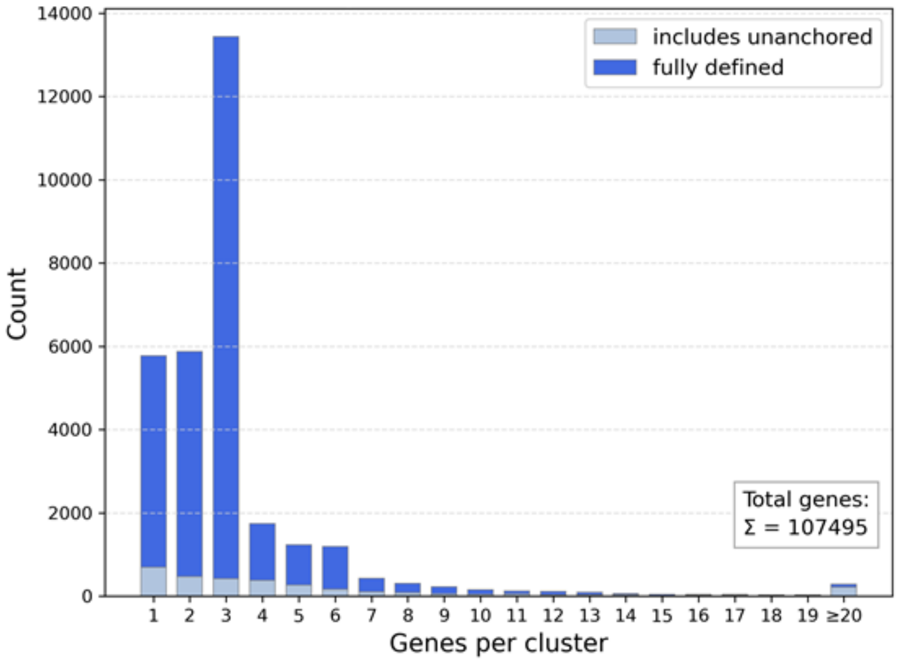

Supplementary Figure 11. Gene family sizes in *A. sativa*. Histogram shows the number of (homoeologous) gene families by the number of oat genes clustering to each family. A total of 107,495 oat genes were grouped into 31,219 families. Dark blue bars indicate the frequency of clusters for which all members are located in one of the 21 pseudo-chromosomes, while light blue families have at least one gene on unanchored scaffolds (‘chr Unknown’).

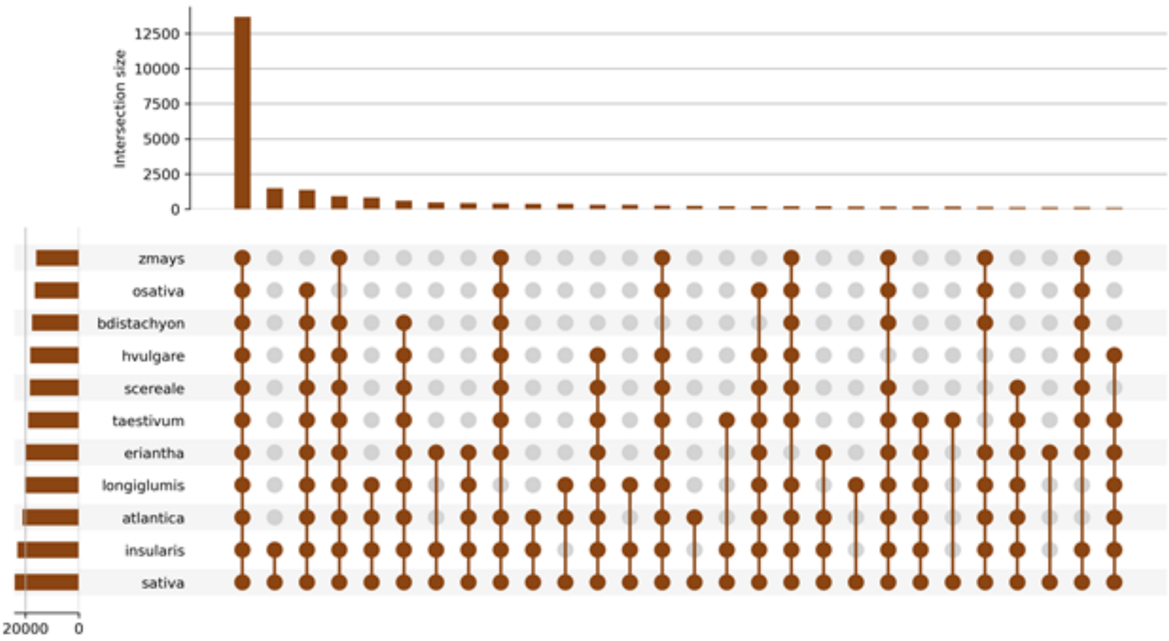

Supplementary Figure 12. Homology support of oat gene families. Homeologous gene families were classified by *Poaceae* species contributing genes to the respective gene family. Lower panel of the upset plot shows all species combinations with ≥ 50 clusters (‘intersection count’).

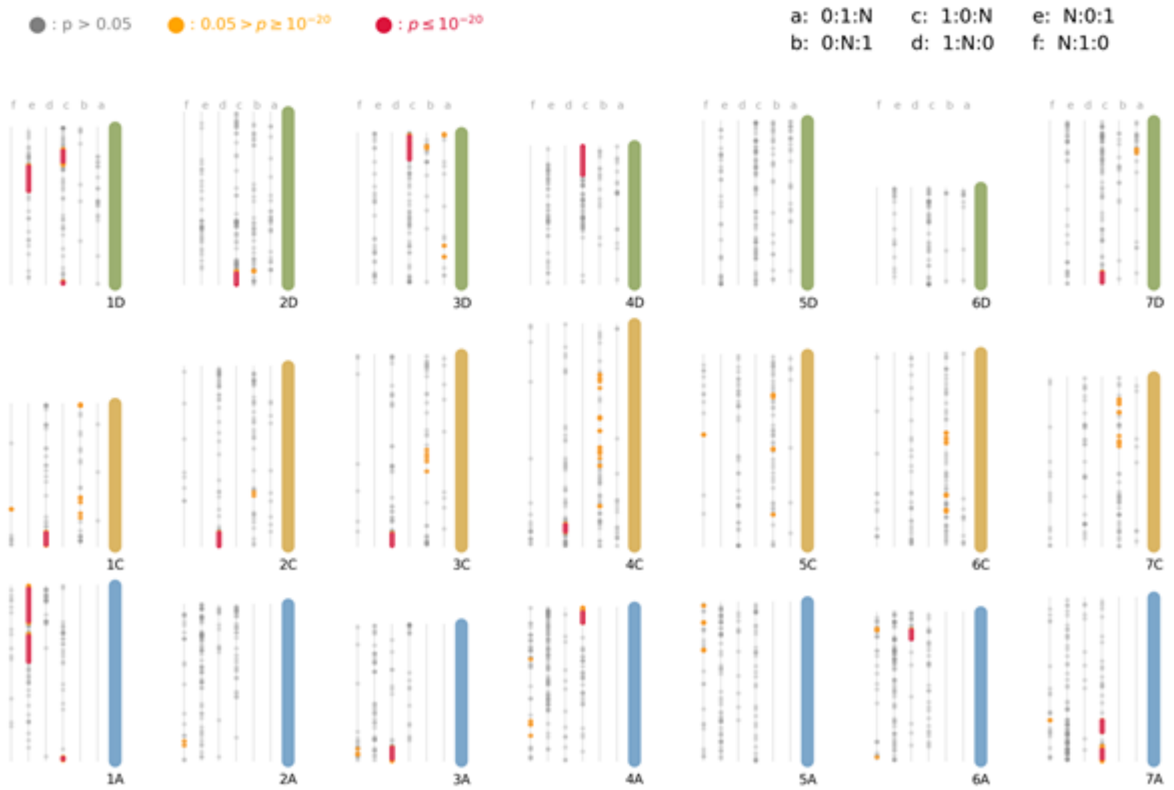

Supplementary Figure 13. Spatial clustering of gene families with a {0,1,N}-multiplicity. Each point along the 21 oat chromosomes represents the centred genomic position of bins of 100 adjacent gene families. Overrepresentation of gene families which contain exactly no, one and more than one gene copies per subgenome, is assessed by a hypergeometric test. The six possible multiplicities are shown at the top right corner and along each chromosome (a-f). Highly significant enrichments are shown in red (panel at top).

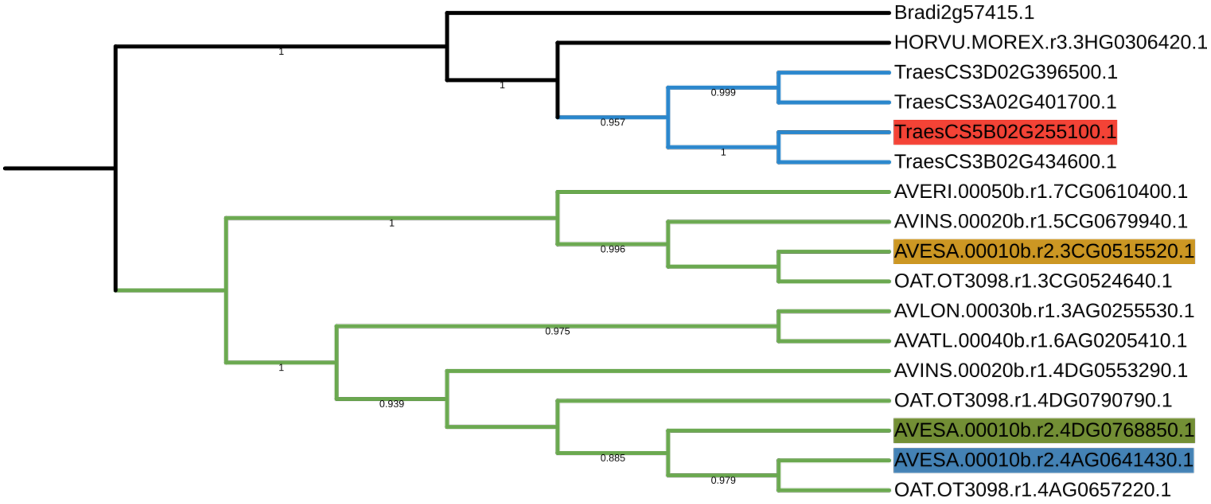

Supplementary Figure 14. Phylogenetic analysis of *TaZIP4-B2* orthologous genes in wheat and oat (including *A. sativa* OT3098), as well as *B. dystachion* and *H. vulgare* as outgroups. blue coloured branches: wheat; green coloured branches: oat clade. *TaZIP4-B2* is shown in red colour and the A, C, and D-oat subgenomes in blue, gold and green respectively. The phylogeny supports the trans-duplicated origin of *TaZIP4-B2* in wheat and the absence of an ortholog in oat.

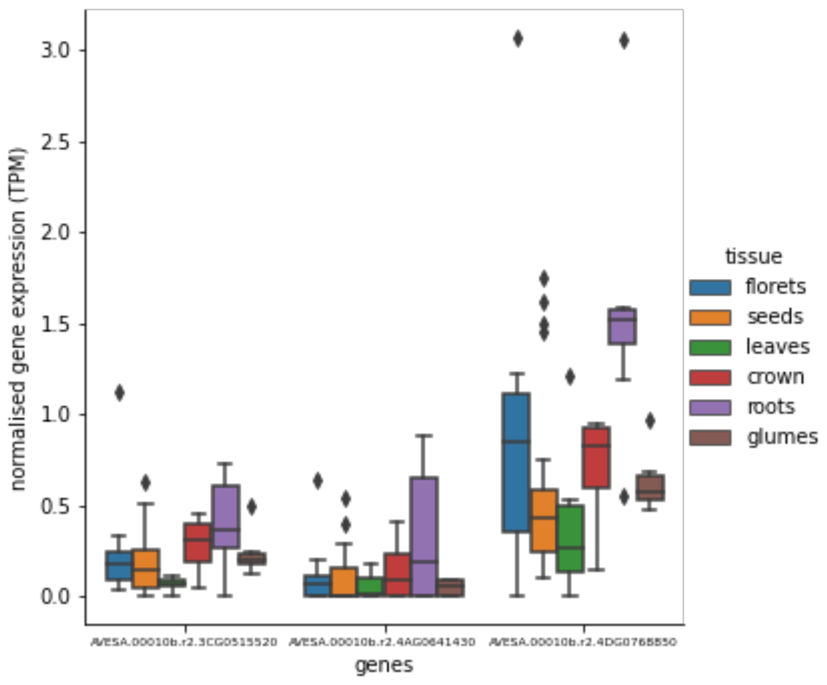

Supplementary Figure 15. Expression of the *A. sativa* Sang orthologs to the wheat *TaZIP4* genes. Expression levels of the oat orthologs (*AVESA.00010b.r2.3CG0515520, AVESA.00010b.r2.4AG0641430,* and *AVESA.00010b.r2.4DG0768850*) to the *TaZIP4* copies on wheat group 3-chromosomes. Expression levels are overall low and comparable to the wheat chromosome group 3 genes and far below the expression of *TaZIP4-B2*[24]. N (number of RNA-seq samples) = 8, 24, 12, 4, 8, 6 for florets, seeds, leaves, crown, roots, and glumes respectively. Values from the first to third quartiles are shown within the boxplots boxes (inter-quartile range) with the median represented by the middle line, the upper and lower whiskers extend from the edge to the largest and smallest value of the edge but no further than 1.5 × the inter-quartile range, the data beyond the end of the whiskers are outlier plotted individually.

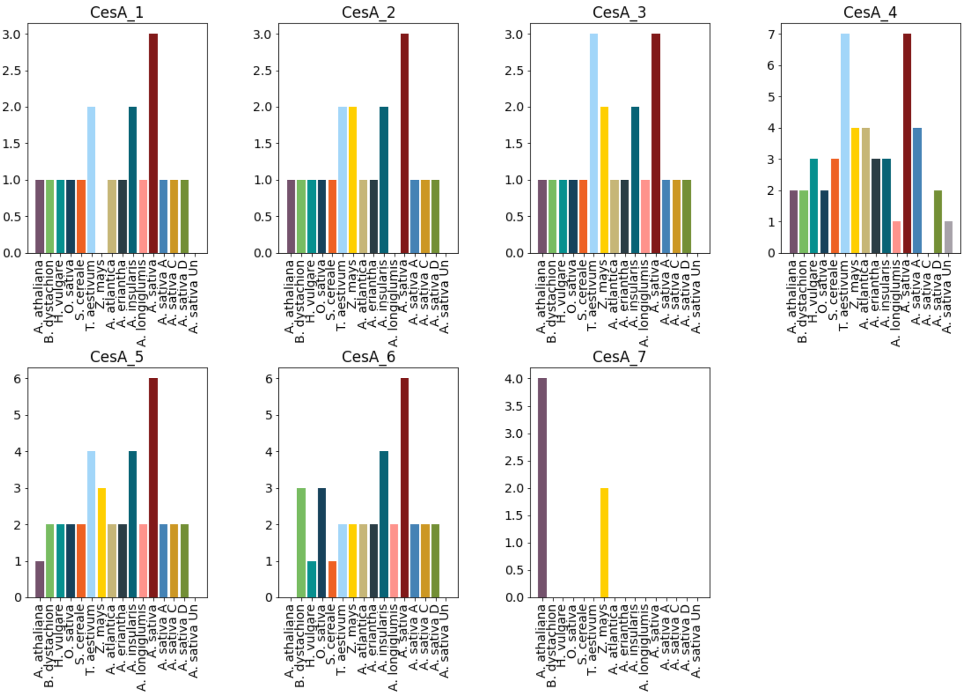

Supplementary Figure 16. Copy number of cellulose synthase (*CesA*) subfamilies in different plant species.

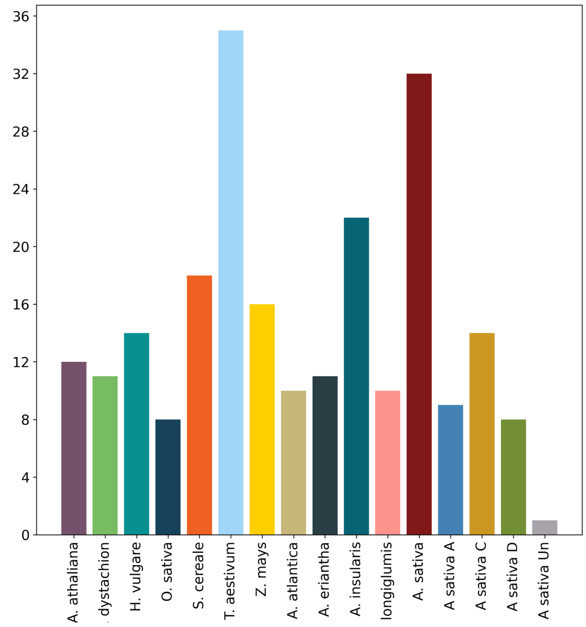

Supplementary Figure 17. Copy number of *GT48* glycosyl transferase (callose synthase) genes in different plant species.

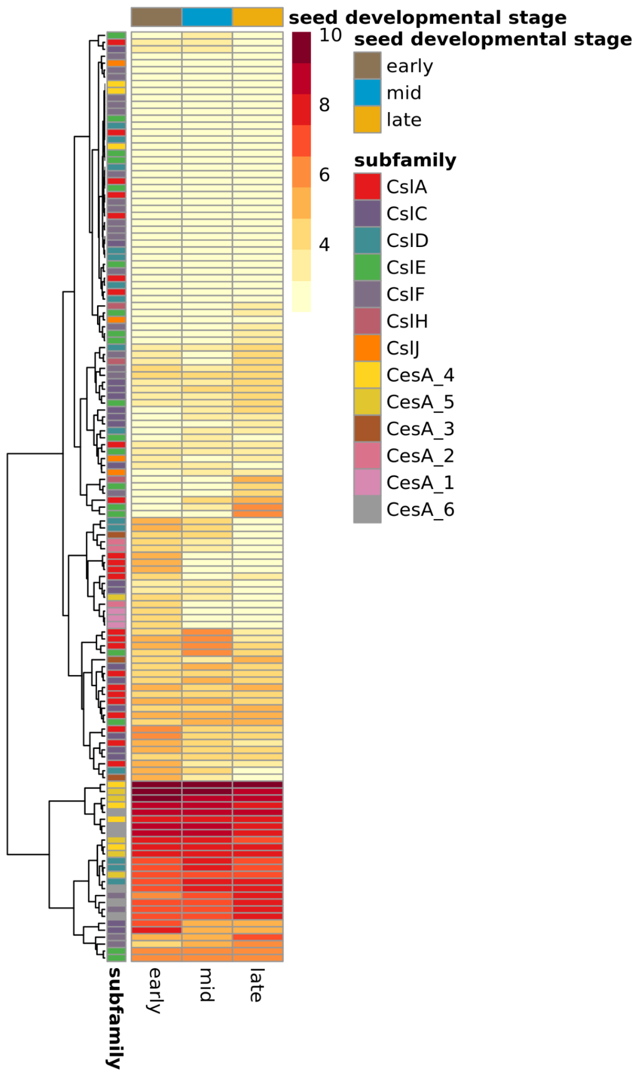

Supplementary Figure 18. Expression of genes of the cellulose synthase gene superfamilies in early, middle, and late stages of seed development. TPM values were variance stabilising transformed and are displayed as colours ranging from yellow to dark red as shown in the colour scale

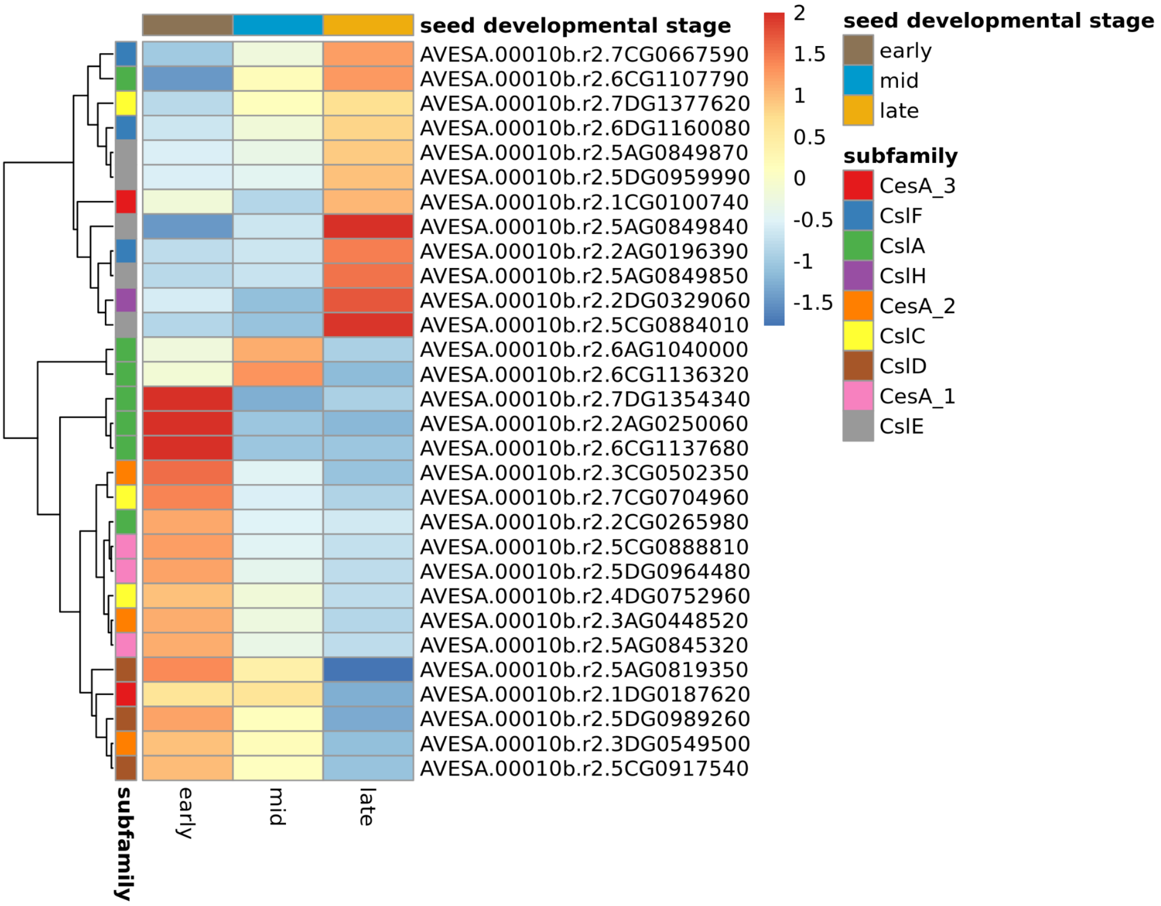

Supplementary Figure 19. Differentially expressed genes of the cellulose synthase gene superfamily between stages of seed development (variance stabilising transformed TPM values). TPM values were variance stabilising transformed and are displayed as colours ranging from red to blue as shown in the colour scale.

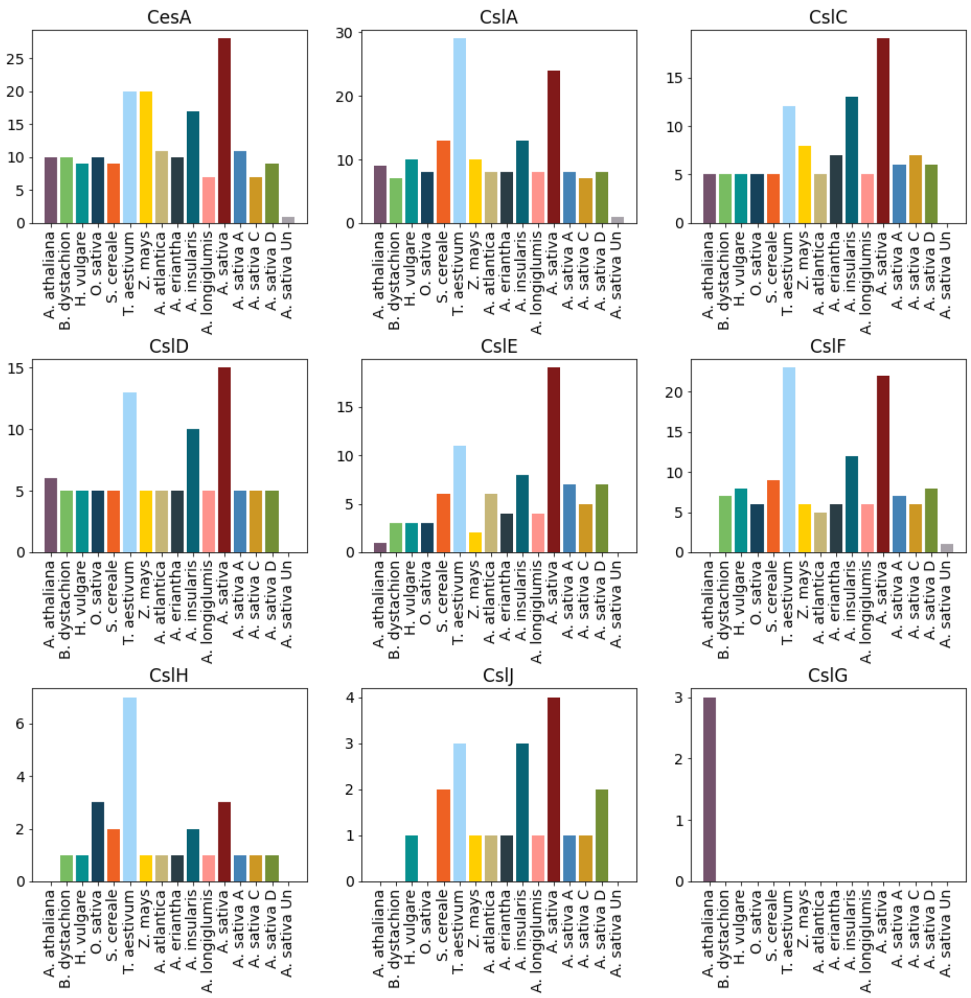

Supplementary Figure 20. Copy number of the members of the cellulose synthase subfamily genes in different plant species.

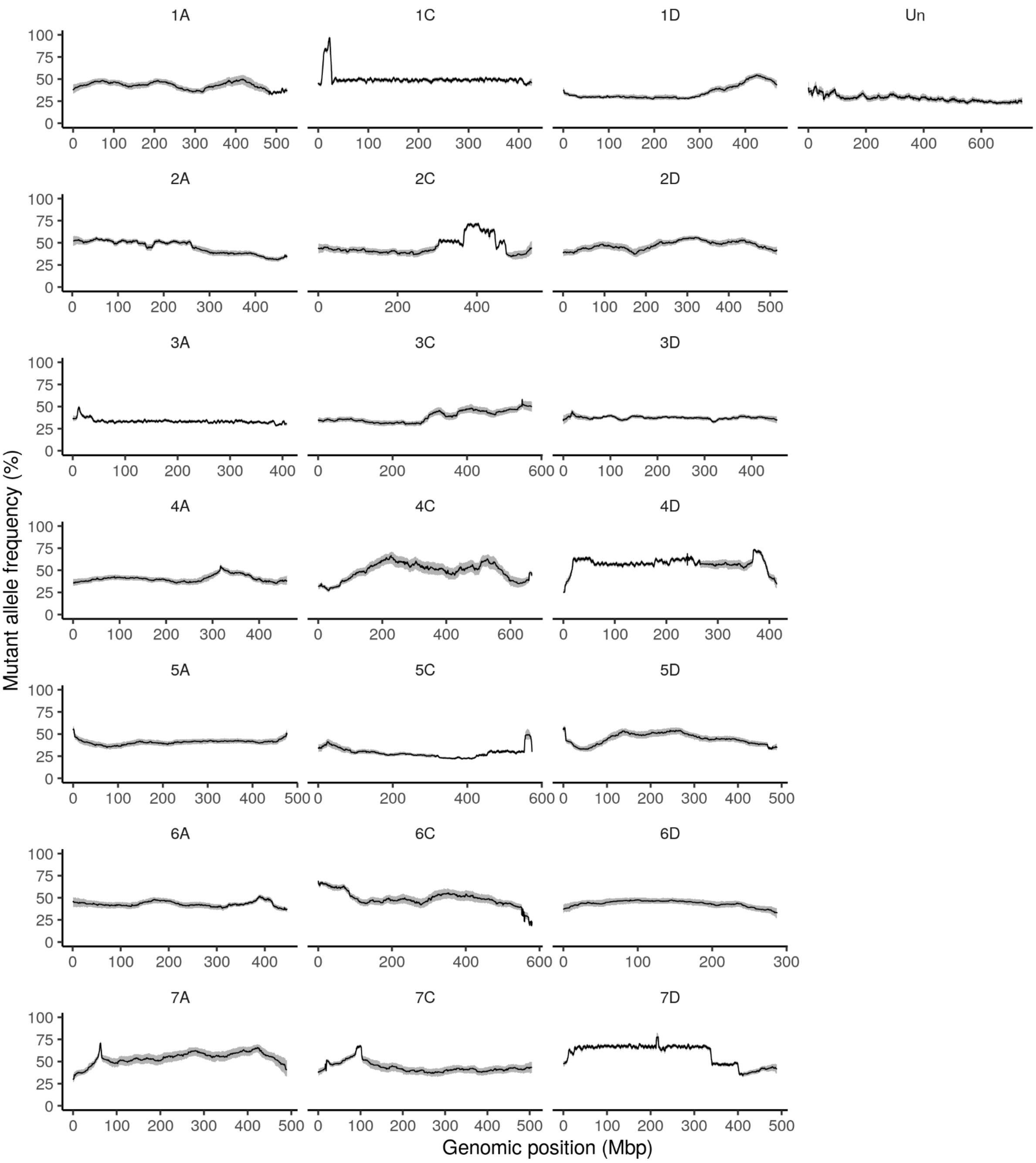

Supplementary Figure 21. Sliding window of allele frequency for variants unique to *glossy.1*. A sliding window of 100 variants (total allelic depth ≥ 30) was used. Grey area indicates 95% bootstrap confidence interval calculated using 1,000 bootstrap samples per chromosome.

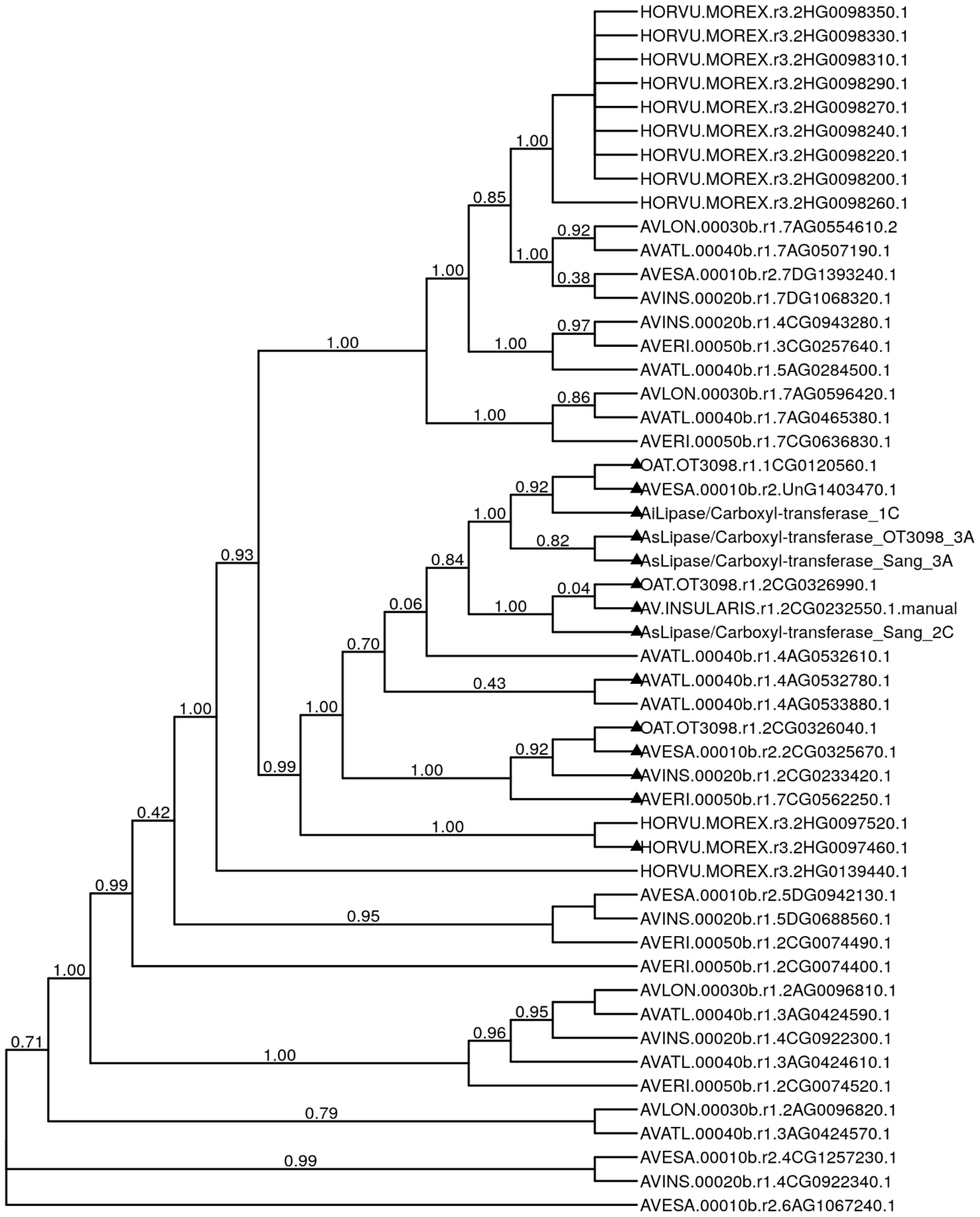

Supplementary Figure 22. Phylogenetic tree of oat and barley protein sequences from orthogroups OG0001044 and OG0028142, as well as genes identified manually in *A. sativa* OT3098 and *A. insularis*. *HORVU.MOREX.r3.2HG0097460.1* is the barley *cer-q* gene. Manually annotated genes starting with 'As' and 'Ai' are found in *A. sativa* and *A. insularis* respectively. Numbers correspond to fasttree local support values. Filled triangles: genes included as a part of the gene clusters.

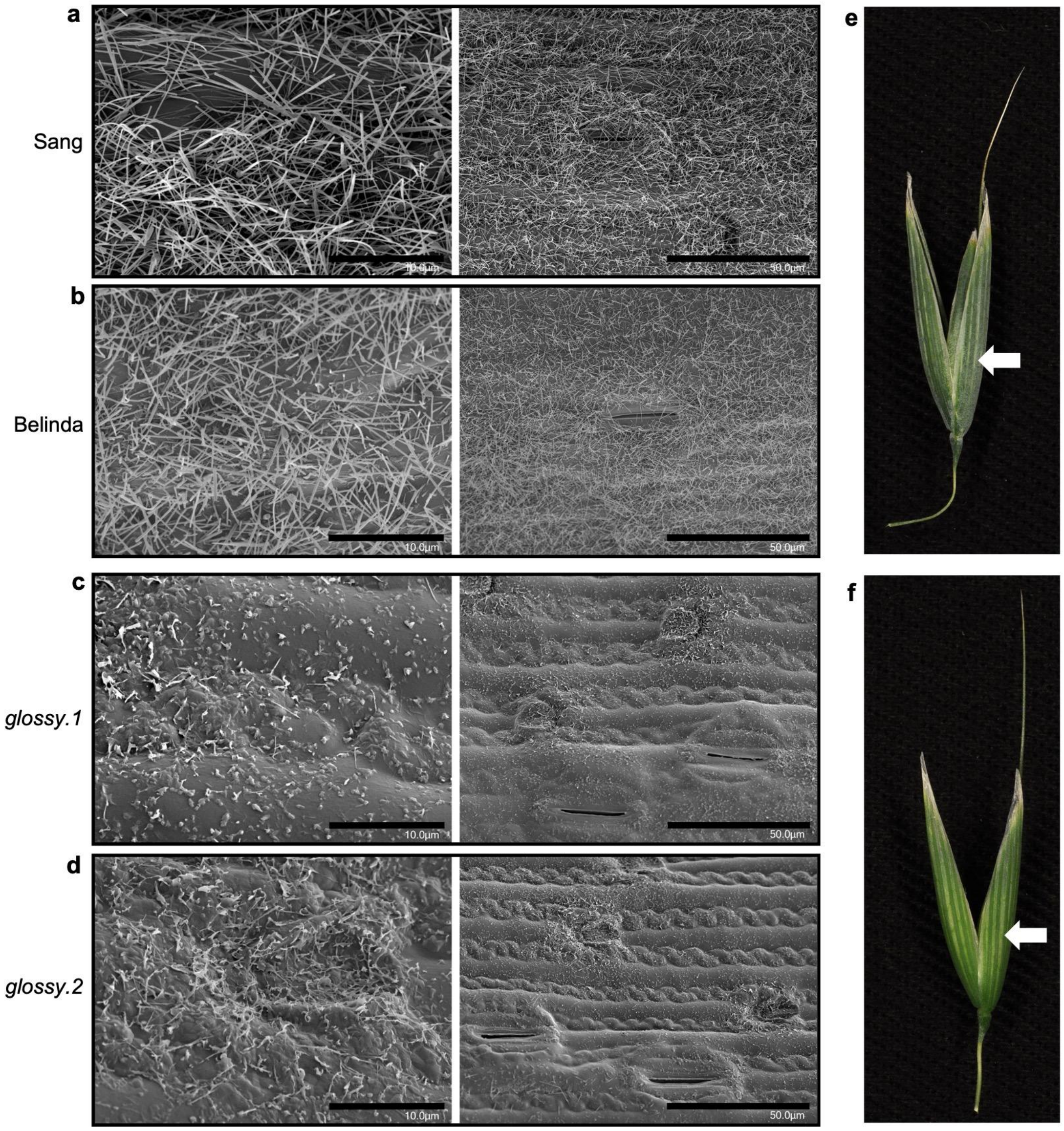

Supplementary Figure 23. Comparison of cuticle surface structures on glume tissue. Electron micrographs were collected at x1000 (**a**-**d**, right, scale bar 50 µm) and x4000 (**a**-**d**, left, scale bar 10 µm) magnification at the glume surface in cv. Sang **a**, cv. Belinda **b**, *glossy.1* **c**, and *glossy.2* **d**, Electron micrographs collected at x1000 (**a**-**d**, right) and x4000 (**a**-**d**, left) magnification. The fields of view shown in **a**-**d** were selected as representative regions based on lower magnification micrographs collected at x10, x50 and x200 magnification (data not shown) from a single representative glume sample from each genotype. These results were confirmed in two biological replicates. Representative whole florets from glaucous **e**, and glossy **f**, genotypes, with the glume indicated by an *arrow*.

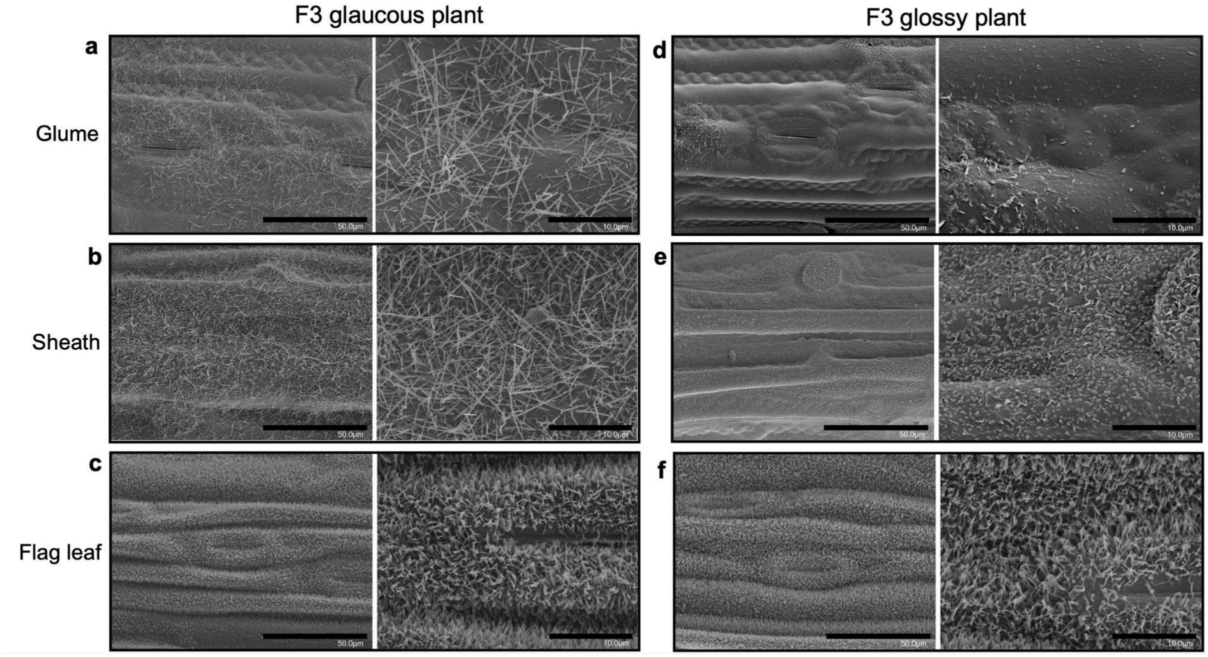

Supplementary Figure 24. Summary of surface wax structures. Comparative SEM investigation of glume, sheath and flag leaf from representative F_3 glaucous and F_3 glossy siblings from the Belinda x *glossy.1* backcross at the early grain filling stage. **a**, **b** and **c** panels are micrographs collected at x1000 (left, scale bar 50 µm) and x4000 (right, scale bar 10 µm) magnification from glume, sheath and flag leaf tissues of the glaucous genotype, respectively. **d**, **e** and **f** panels are comparable micrographs from the glossy genotype. A dense coating of long tubules (0.1-0.2 µm in diameter, up to 25 µm long) cover the sheath and glume surfaces (**a**, **b**) in the glaucous genotype. These structures are absent in the corresponding *glossy.1* tissues (**d**, **e**), instead we observe granules, irregular plates and short thin threads. A dense coating of interconnecting lobed plates (0.1 µm thick, up to 3 µm long) were observed on the flag leaf of both glaucous (**c**) and glossy (**f**) genotypes. The fields of view shown in **a**-**f** were selected as representative regions based on lower magnification micrographs collected at x10, x50 and x200 magnification (data not shown) from a single representative glume, sheath and flag leaf sample from each genotype. These results were confirmed in two biological replicates.

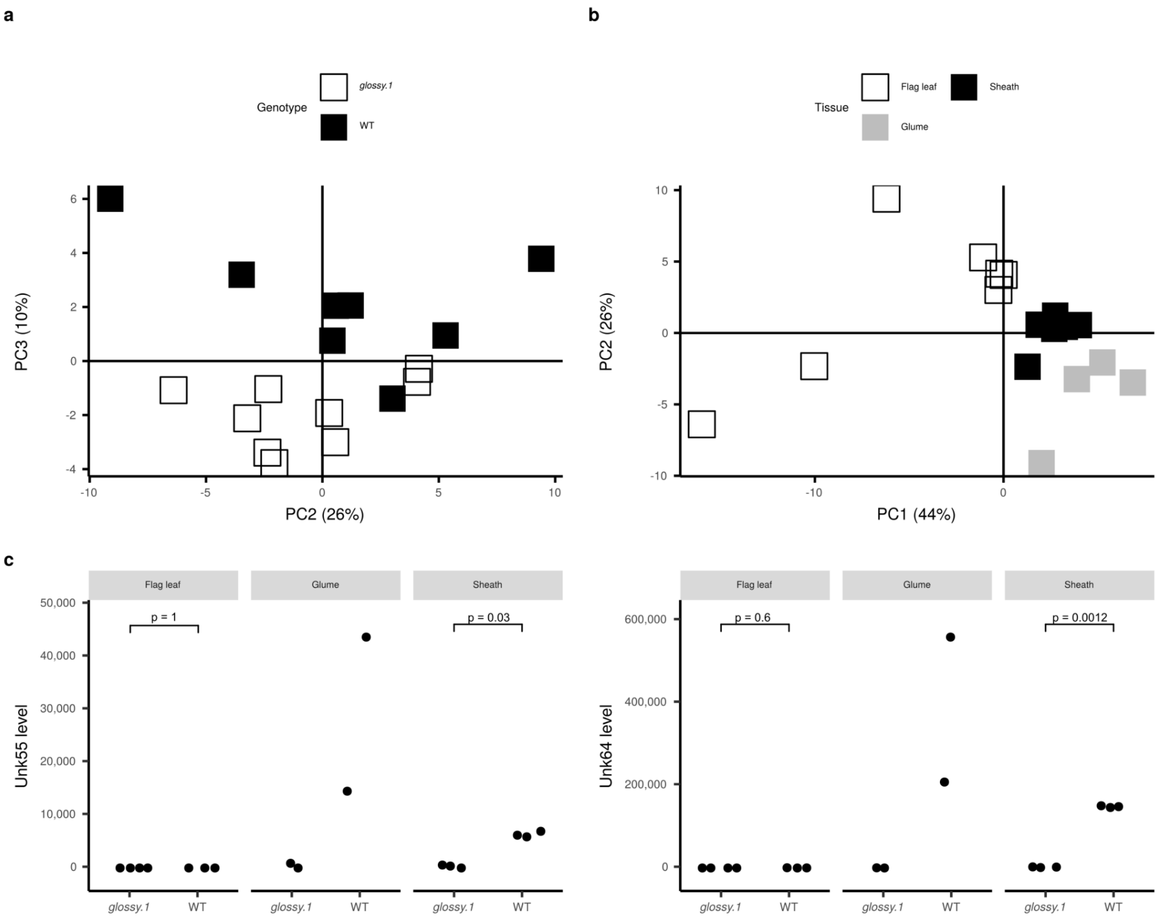

Supplementary Figure 25. Profiling of metabolites in various tissues of the glossy and glaucous genotypes. **a,** Principal component analysis (PCA) score plot revealing a systematic difference in wax profiles in the glossy and glaucous (WT) oat genotypes. Clustering of genotypes is observed in principal components (PCs) 2 and 3. **b,** Clustering of the different tissues are observed in PC1 and PC2. **c,** Three metabolites (including hentriacontane-14,16-dione shown in Fig. 4g) differed significantly between genotypes in sheath. Two-sided Welch t-tests were used to determine if metabolite levels were equal in glossy and glaucous samples with p-values adjusted using Benjamini-Hochberg procedure (glaucous flag leaf *n*=4; glossy flag leaf *n*=3; sheath *n*=3; glume *n*=2).

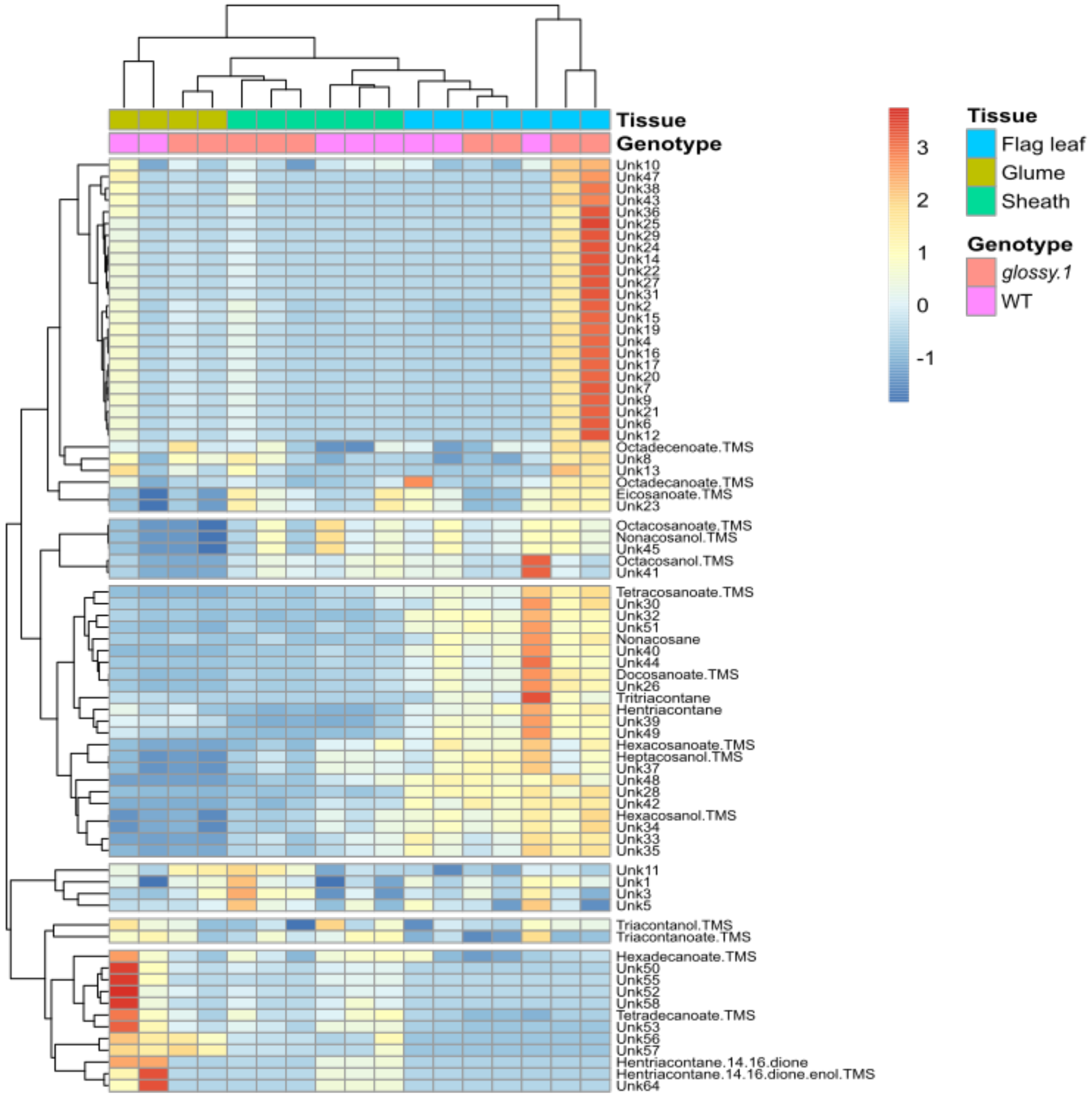

Supplementary Figure 26. Heatmap of detected metabolite levels across tissues and genotypes. Detected levels are scaled and centred feature-wise. Out of the 76 features detected, none differed between genotypes for flag leaves, hentriacontane-14,16-dione and two unknown features differed between genotypes for sheath (two-sided Welch's t-test, p-value adjusted using Benjamini-Hochberg, adjusted $p < 0.05$; glaucous flag leaf n=4; glossy flag leaf n=3; sheath n=3; glume n=2). As only two replicates were used for glume, no statistical analysis was done, but a reduction in hentriacontane-14,16-dione in glossy relative to glaucous glume is shown in Fig. 4g. Hentriacontane-14,16-dione is a beta-diketone, which can self-assemble into tubule structures at the plant cuticle. These tubules are absent in *glossy.1* cuticles (Supplementary Fig. 23). These results indicate that the bright green visual appearance of the *glossy.1* mutant panicle and sheath may be caused by a lack of tubule wax crystals primarily composed of hentriacontane-14,16-dione.

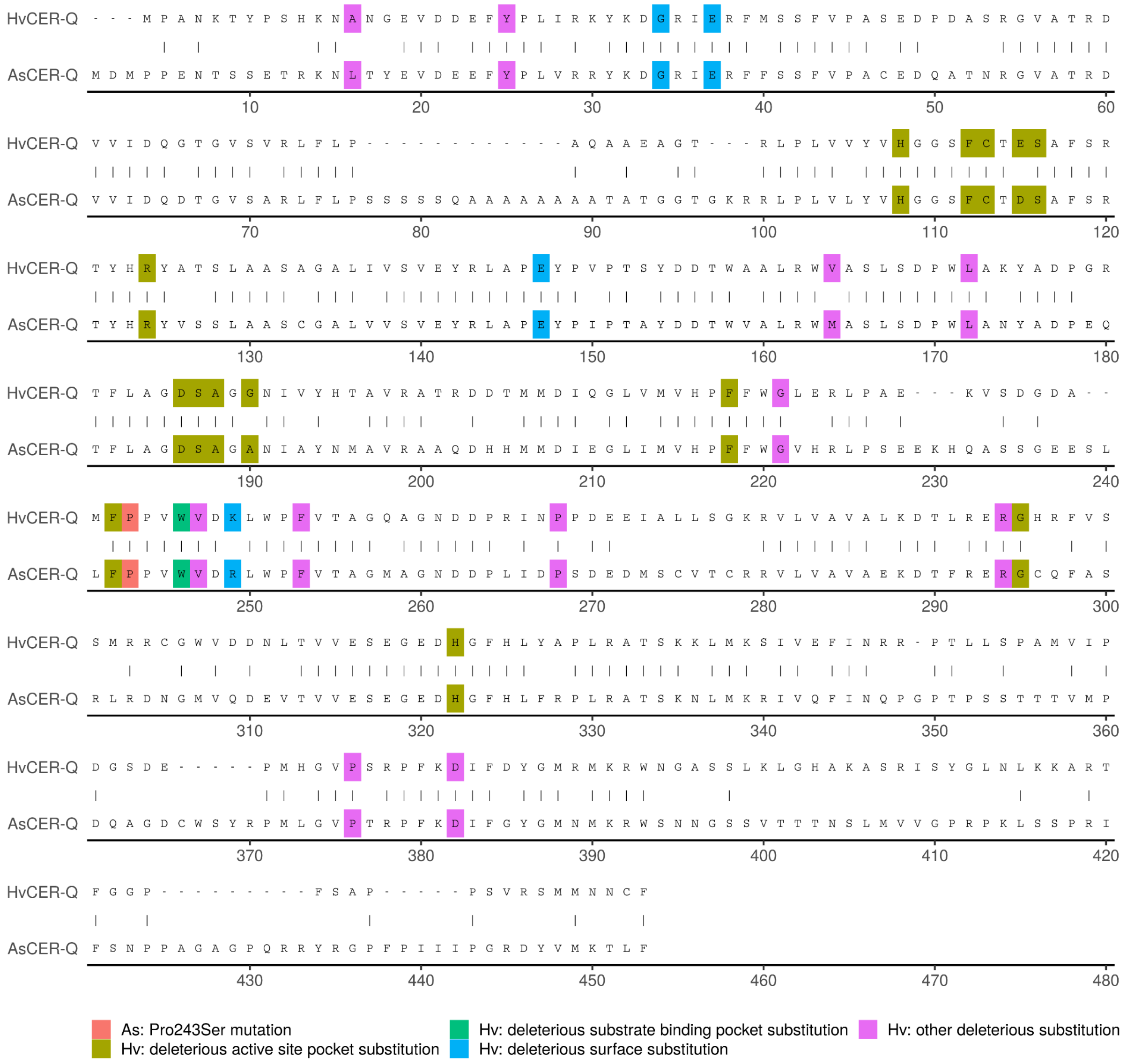

Supplementary Figure 27. Alignment of HvCER-Q and AsCER-Q with highlighted deleterious mutations. HvCER-Q (MLOC_13397, Uniprot ID: M0URA9) and AsCER-Q (*AVESA.00010b.r2.UnG1403470.1*). Vertical bars indicate identical residues. Highlighted: Hv: known deleterious single amino acid substitutions from barley and the part of the protein they are located in[49]. As: the Pro243Ser mutation identified in *AVESA.00010b.r2.UnG1403470.1*.

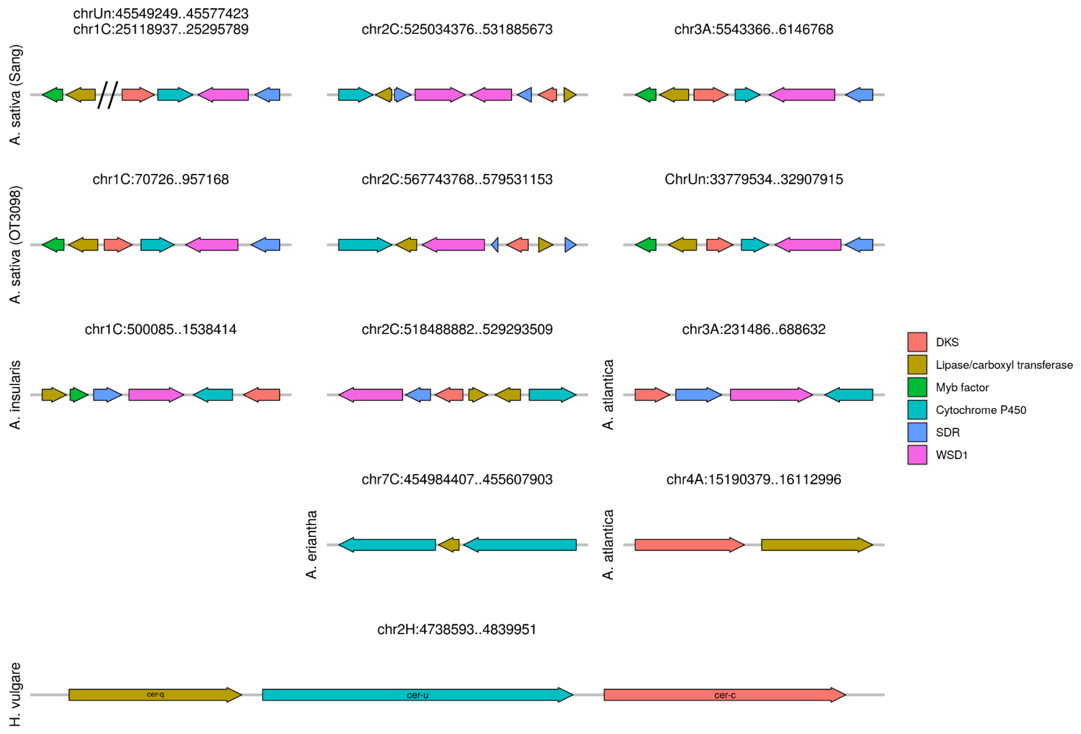

Supplementary Figure 28. Gene clusters identified in *A. sativa* Sang and OT3098, *A. insularis*, *A. atlantica*, and *A. eriantha*, as well as the *cer-cqu* cluster known from barley. The beta-diketone synthases (DKSs, *cer-c* in barley) and lipases (*cer-q* in barley) belong to the same clade of the phylogenetic trees in all clusters (Supplementary Figs. 22 and 29). The cytochrome P450s are orthologous in the *Avena* species, but not to *cer-u* (Supplementary Fig. 30). The Myb factor, Wax ester synthase/diacylglycerol acyltransferase 1 (WSD1), and the short-chain dehydrogenase/reductase (SDR) are not found in the barley cluster but are present close to the *cer-cq* orthologs in several of the *Avena*s (Supplementary Figs. 31-33). The orientation of the Myb factor and the lipase on Sang chromosome 1C is inferred based on the orientation in OT3098. Gene lengths are scaled relative to other genes within the same chromosome, and include introns. The cluster on ChrUn in OT3098 has been flipped to align with the cluster on 3A in Sang. Relative to barley, the cluster in wheat is known to have undergone duplications[48] - in oat we see both reordering of genes relative to barley, but also that the clusters are not only located on chromosome 2. The clusters on 1C and 2C are present in both *A. sativa* and in the CD tetraploid *A. insularis*. The cluster on 1C is located in a region identified as translocated from the D subgenome, in between two blocks syntenic to chromosomes 3A and 3C. The syntenic block closest to the 2C cluster is syntenic to chromosomes 2A and 2D. The 3A cluster is located in a block syntenic with 1C and 3C. These syntenic blocks are shown in Extended Data Fig. 3. The A genome diploid *A. atlantica* has genes homologous to the cluster genes on both chromosomes 3 and 4. On chromosome 7 the C genome diploid *A. eriantha* has a lipase/carboxyl transferase and two cytochrome P450s homologous to cluster genes.

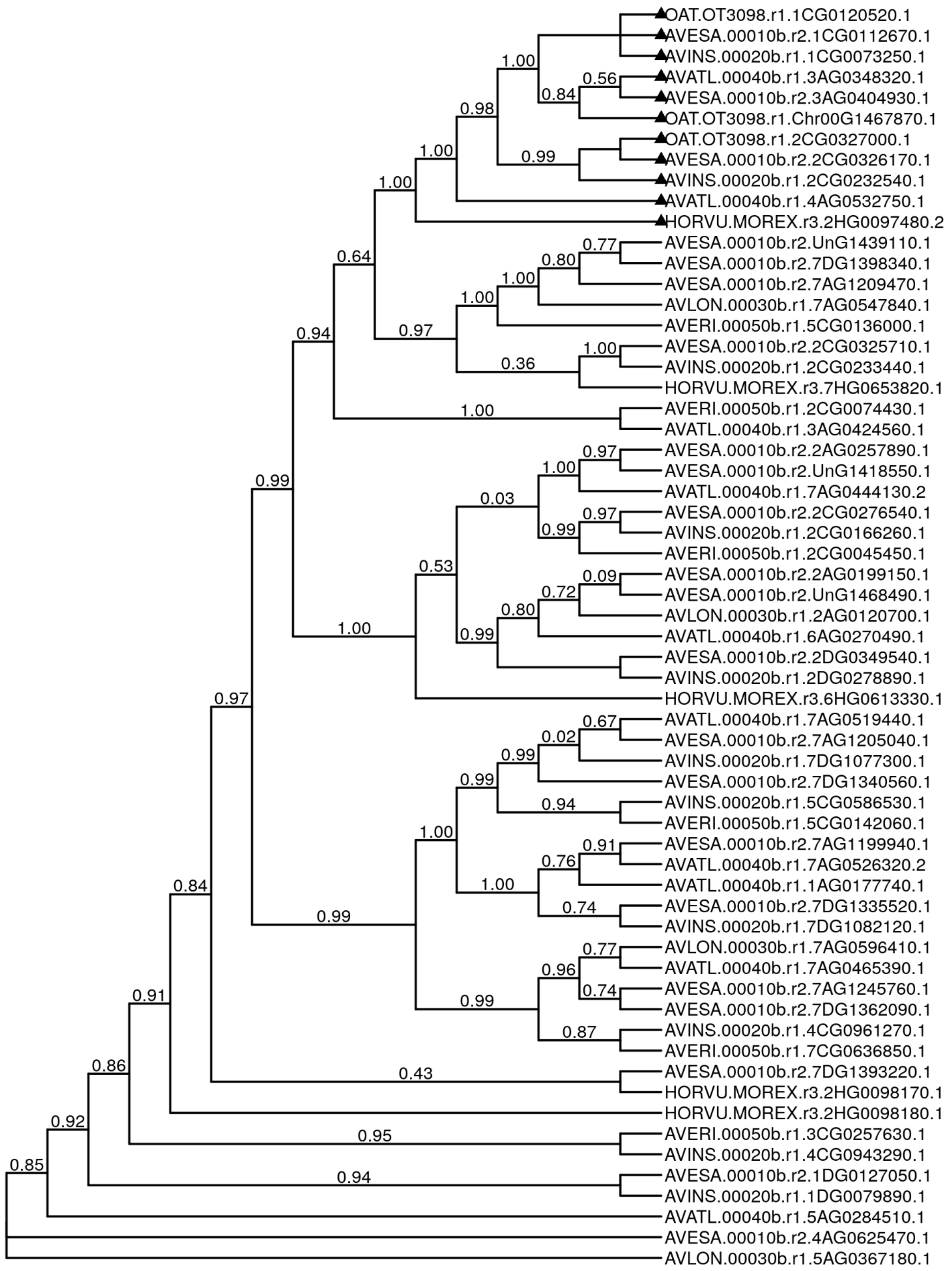

Supplementary Figure 29. Phylogenetic tree of oat and barley protein sequences from orthogroup OG0000616, as well as genes identified manually in *A. sativa* OT3098. *HORVU.MOREX.r3.2HG0097480.2* is the barley *cer-c* gene. Numbers correspond to fasttree local support values. Filled triangles: genes included as a part of the gene clusters.

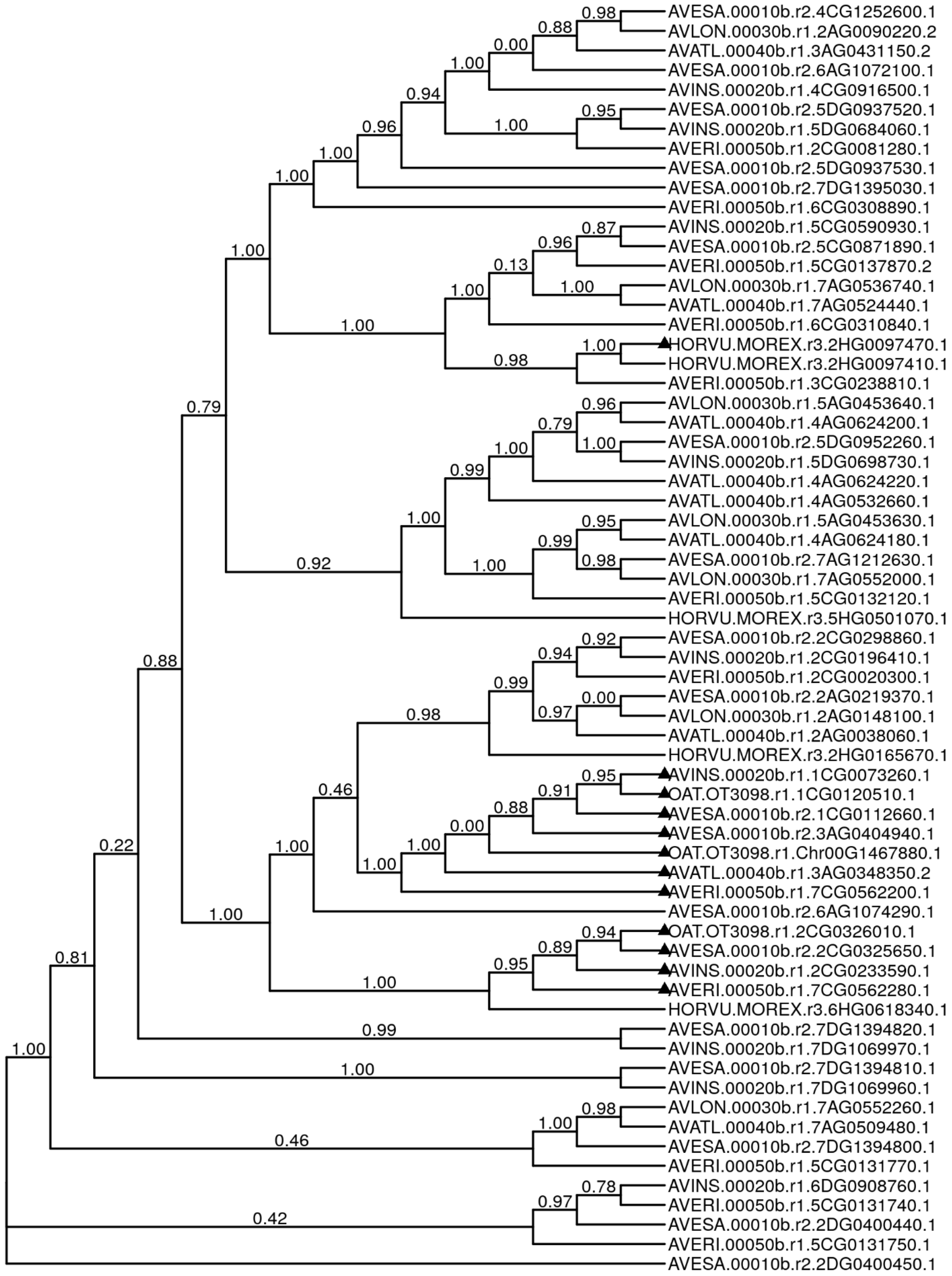

Supplementary Figure 30. Phylogenetic tree of oat and barley protein sequences from orthogroups OG0001150, OG0006251, OG0015717 as well as genes identified manually in *A. sativa* OT3098. *HORVU.MOREX.r3.2HG0097470.1* is the barley *cer-u* gene. Numbers correspond to fasttree local support values. Filled triangles: genes included as a part of the gene clusters.

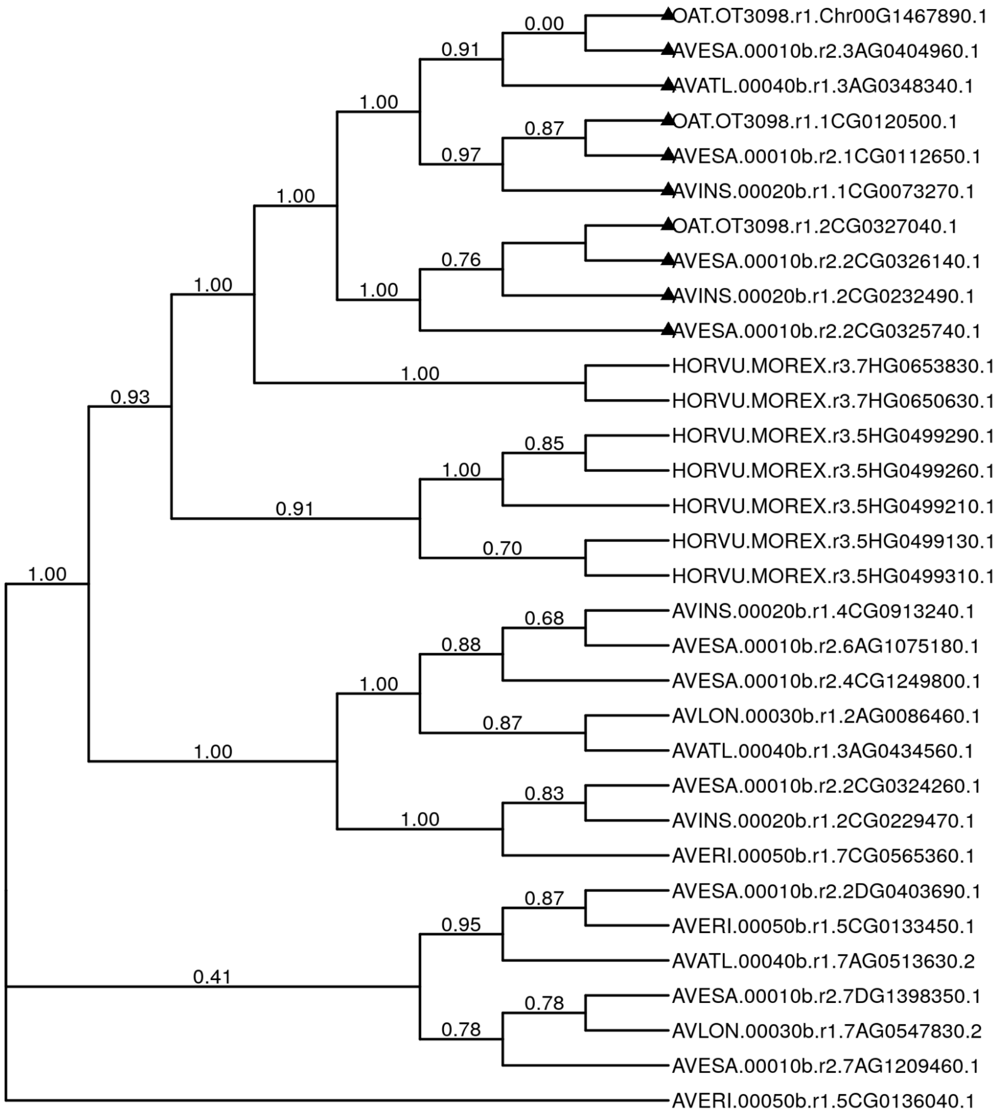

Supplementary Figure 31. Phylogenetic tree of oat and barley protein sequences from orthogroup OG0001279 as well as genes identified manually in *A. sativa* OT3098. These genes have a functional annotation identifying them as similar to Wax ester synthase/diacylglycerol acyltransferase 1 (WSD1). Numbers correspond to fasttree local support values. Filled triangles: genes included as a part of the gene clusters.

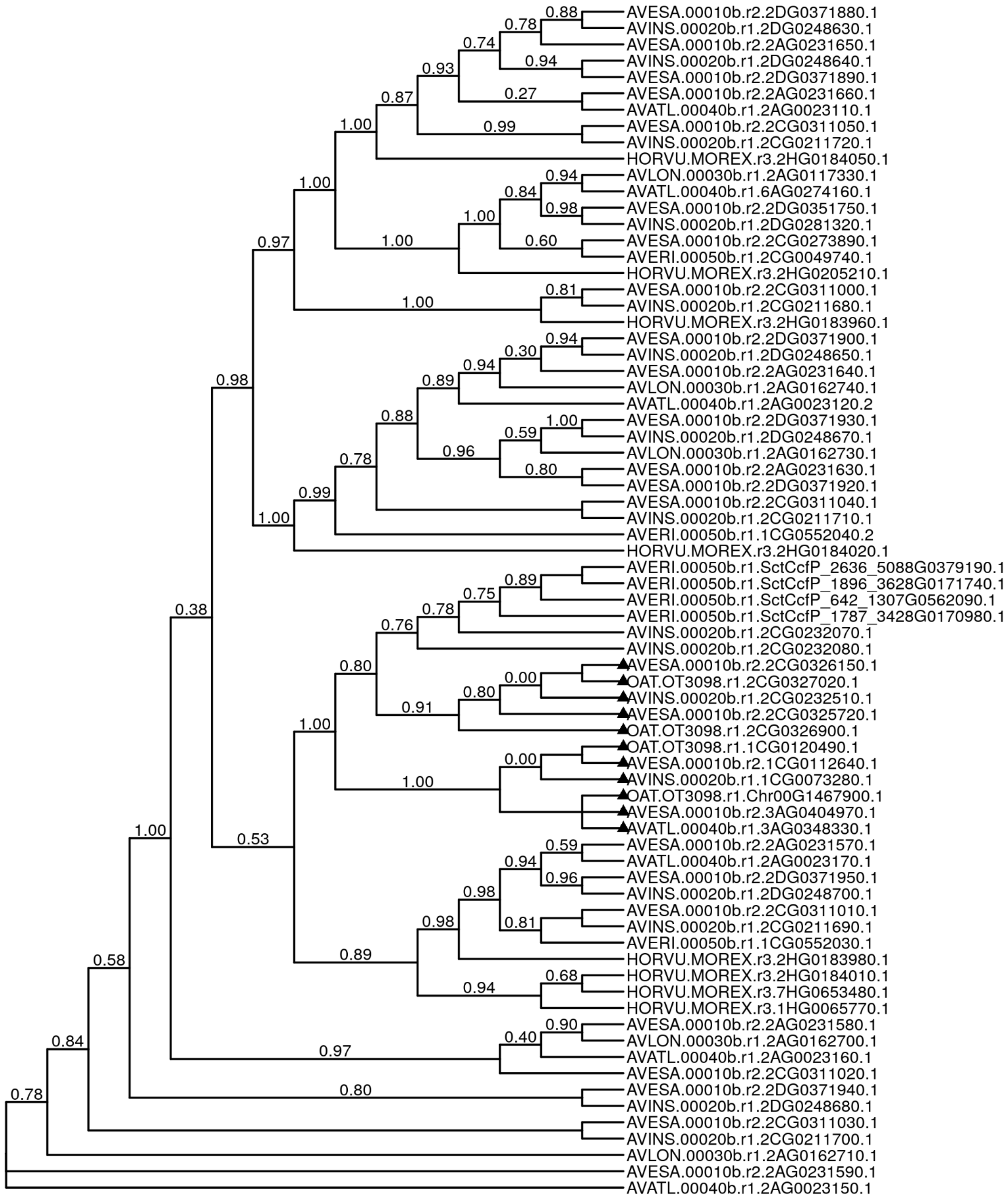

Supplementary Figure 32. Phylogenetic tree of oat and barley protein sequences from orthogroup OG0000526 as well as genes identified manually in *A. sativa* OT3098. These genes have a functional annotation identifying them as short-chain dehydrogenase/reductases (SDR). Numbers correspond to fasttree local support values. Filled triangles: genes included as a part of the gene clusters.

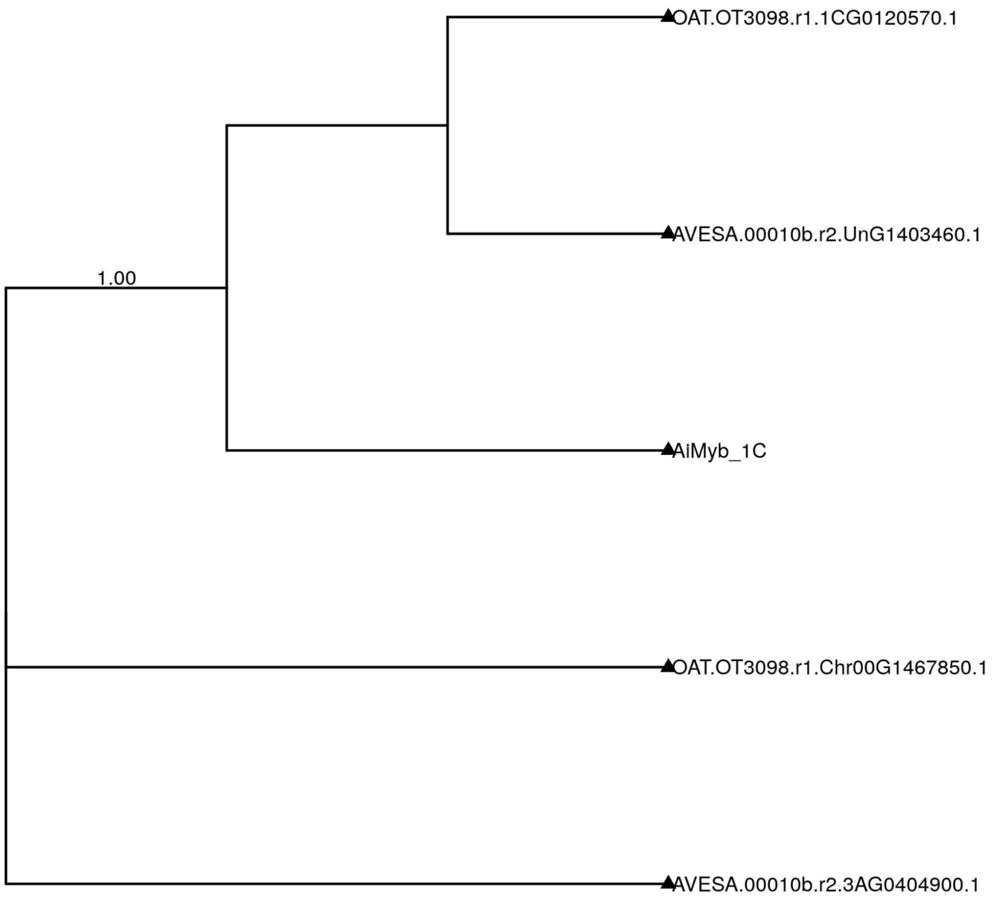

Supplementary Figure 33. Phylogenetic tree of protein sequences from orthogroup OG0073676 as well as genes identified manually in *A. sativa* OT3098 and *A. insularis*. These genes have a functional annotation identifying them as Myb factors. The manually annotated gene starting with 'Ai' is found in *A. insularis*. Filled triangles: genes included as a part of the gene clusters.

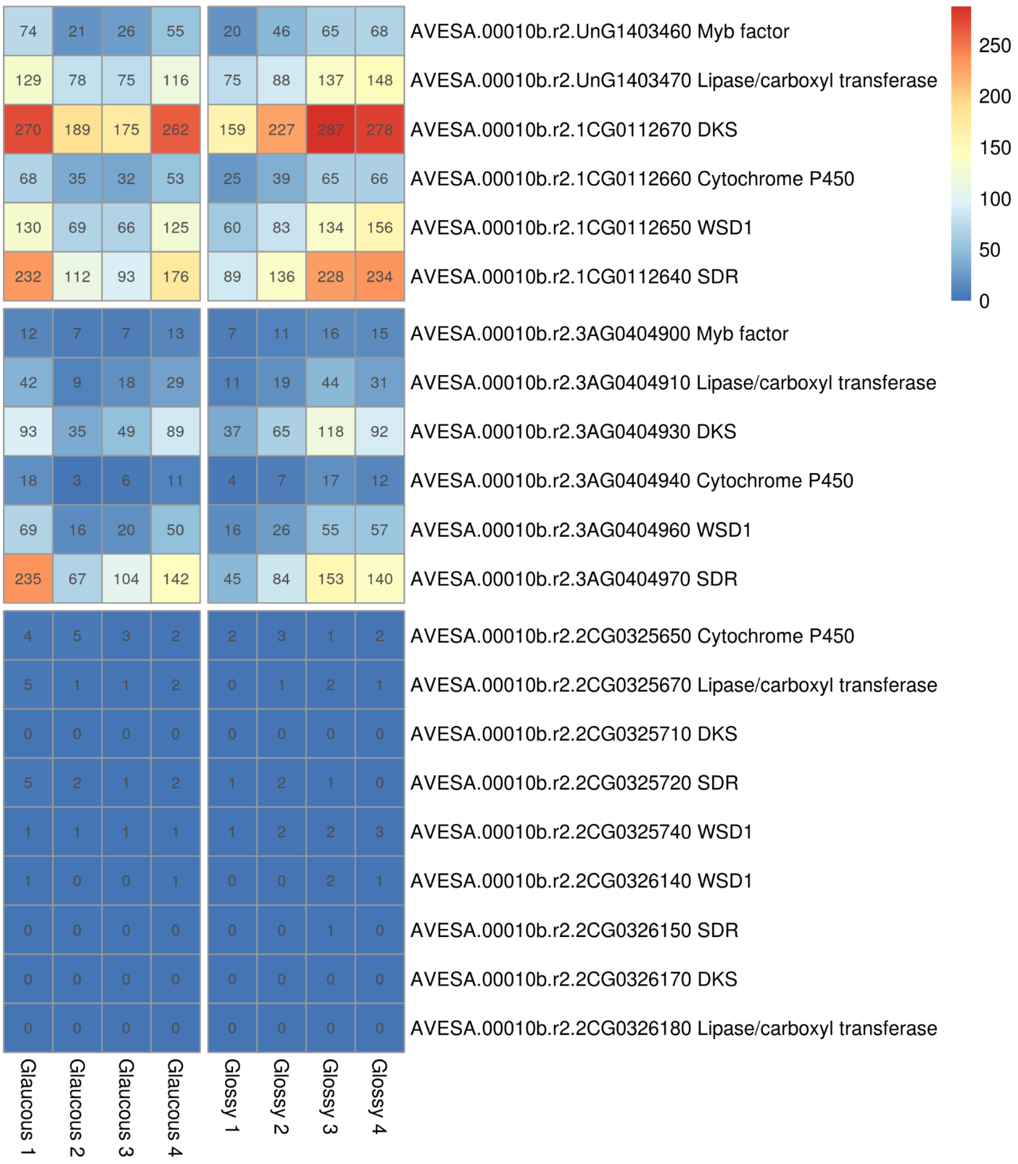

Supplementary Figure 34. Gene-level abundances (TPM) for within-sample expression comparisons of *A. sativa* Sang genes found in the identified clusters. Genes: Myb factor, Lipase/carboxyl transferase (*cer-q* in barley), beta-diketone synthase (DKS, *cer-c* in barley), Cytochrome P450, Wax ester synthase/diacylglycerol acyltransferase 1 (WSD1) and short-chain dehydrogenase/reductase (SDR).

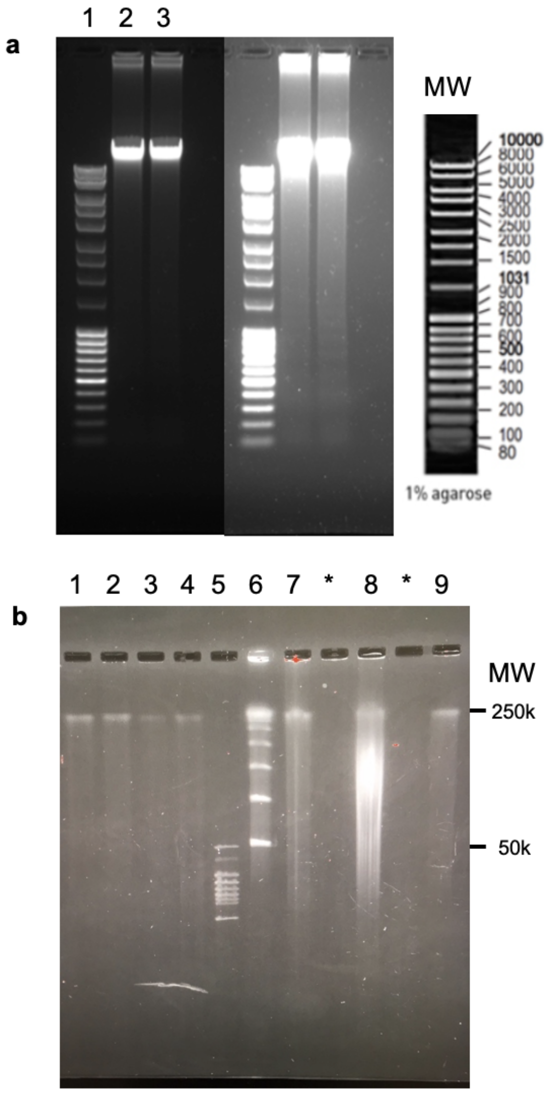

Supplementary Figure 35. a, 1% agarose gel electrophoresis image of cv. Sang hexaploid oat genomic DNA used for the construction of the 470 bp -10 kb sequencing libraries. Sufficient DNA for these sequencing libraries was extracted from one bulk sample of etiolated leaves at the one leaf stage. 500 ng gDNA is loaded in lanes 2 and 3 from two independent isolations from etiolated leaves, shown with optimal exposure (left) and over-exposure (right). The illustrated molecular weight (MW) marker is loaded in lane 1. **b**, Pulse-field agarose gel electrophoresis image of ultra-high molecular weight (uHMW) DNA isolated from etiolated hexaploid oat nuclei. The High Range MW marker in lane 5 has a max MW band of 50kb, and the lambda ladder in lane 6 begins at 50kb and increases with 50 kb increments (maximum of 250 kb). Lanes 1-4 contain the isolated uHMW oat genomic DNA used for 10X Genomics Chromium genome libraries. In total, seven uHMW DNA samples were extracted independently from 5 g of etiolated leaf samples (lanes 1-4, 7, 8, 9). *Asterisk* denotes empty lanes.

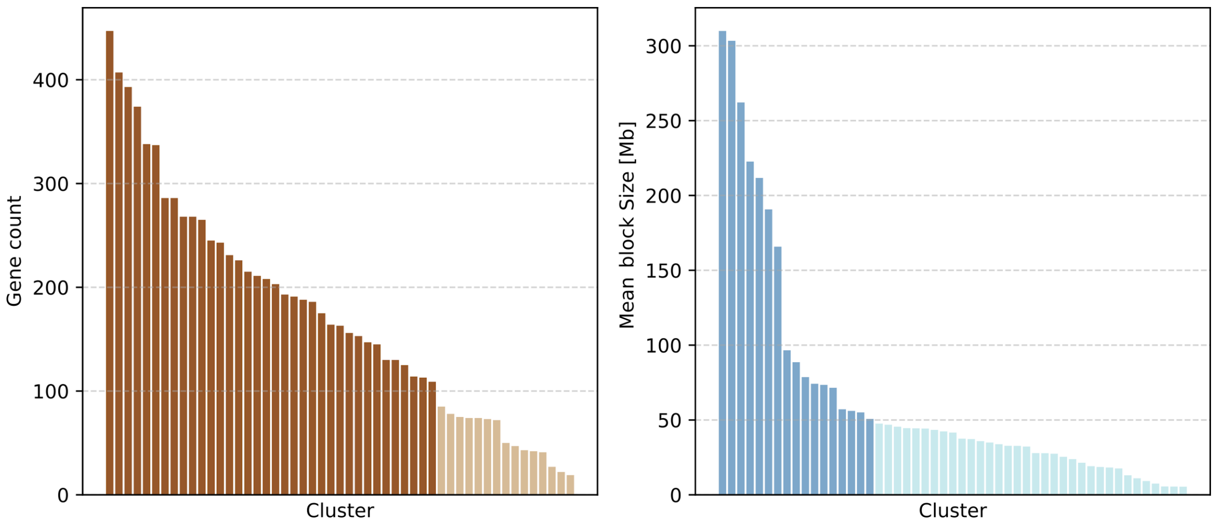

Supplementary Figure 36. Number of genes (right) and mean genomic spans (left) of syntenic blocks. Right panel shows the number of genes per block, dark brown columns indicate blocks with ≥ 100 orthologs or homeologs per species or subgenome, respectively. Left panel shows the mean block size within the four *Avena* species. Blocks larger or equal than 50 Mb are highlighted in darker accents.

Supplementary Tables 1, 4-6, 8-10, 21

Supplementary Tables 2, 3, 7, 11-20, 22 are provided in a separate excel document.

Supplementary Table 1: Hexaploid oat cv. Sang assembly statistics.

	Contigs	Scaffolds
Total sequences	1,818,443	523,398
Assembly size	10,240,882,062	10,959,045,696
Gaps %	0	6.1491
N50	21,026	17,728,407
N50 #sequences	132,376	150
N90	2,020	2,801,443
N90 #sequences	704,007	693
MAX	342,072	113,812,991

Supplementary Table 4: Comparison of repetitive element types between the short read Sang assembly and the long read OT3098 assembly. The first two columns show the annotated amounts in megabases for the respective repeat type, the third column contains the OT3098/Sang ratio.

**repeat type (Mb)**	**Sang**	**OT3098**	**OT3098 versus Sang**
Transposons	6529.5	6700.6	1.03
Tandem repeats	633.2	901.2	1.42
rDNA	4.0	17.5	4.39

Supplementary Table 5: Transposon composition of the Sang and OT3098 genome assemblies. The third column shows the OT3098/Sang ratio.

	Sang	OT3098	OT3098 versus Sang
	% of assembly		
Mobile Element (TXX)	63,76	61,90	0,97
**Class I: Retroelement (RXX)**	**61,68**	**59,89**	**0,97**
LTR Retrotransposon (RLX)	61,62	59,83	0,97
Ty1/copia (RLC)	13,35	14,03	1,05
Ty3/gypsy (RLG)	30,54	29,00	0,95
unclassified LTR (RLX)	17,73	16,80	0,95
non-LTR Retrotransposon (RXX)	0,06	0,06	0,95
LINE (RIX)	0,06	0,06	0,95
SINE (RSX)	0,01	0,01	0,93
**Class II: DNA Transposon (DXX)**	**1,83**	**1,84**	**1,00**
DNA Transposon Superfamily (DTX)	1,65	1,67	1,01
CACTA superfamily (DTC)	1,43	1,46	1,02
hAT superfamily (DTA)	0,02	0,02	1,12
Mutator superfamily (DTM)	0,08	0,08	1,00
Tc1/Mariner superfamily (DTT)	0,04	0,03	0,94
PIF/Harbinger (DTH)	0,07	0,07	0,94
other transposon family (DTX)	0,00	0,00	0,78
unclassified (DTX)	0,01	0,01	0,96
DNA Transposon Derivative (DXX)	0,16	0,16	0,95
MITE (DXX)	0,16	0,16	0,95
Helitron (DHH)	0,01	0,01	0,94
unclassified DNA transposon (DXX)	0,01	0,00	0,78
Unclassified Element (TXX)	0,25	0,17	0,68
Retro-TE/DNA-TE ratio	33,7	32,6	0,97
Gypsy/Copia ratio	2,3	2,1	0,91

Supplementary Table 6: Tandem repeat composition of the Sang and OT3098 genome assemblies. The third column shows the OT3098/Sang ratio.

	**Sang**		**OT3098**		**OT3098 versus Sang**
	**Mb**	**% of assembly**	**Mb**	**% of assembly**	**% of assembly**
Tandem repeats	633.2	5.75	901.2	8.31	1.4
Microsatellite (2-9)	5.3	0.05	22.8	0.21	4.4
Minisatellite (10-99)	193.8	1.76	469.3	4.33	2.5
Satellite (>=100)	434.1	3.94	409.2	3.77	1.0

Supplementary Table 8: Genomic position and sizes of inter-subgenomic translocations. Positions are estimated from the subgenomic kmer boundaries, labels of the 8 translocation events correspond to numbers in Extended Data Fig. 5. Column 'type' shows the subgenomic source and target of the respective translocation. (*) indicates that translocation T2 has occurred in the tetraploid ancestor of *A.insularis* and *A.sativa*, but has undergone a secondary translocation event T7 specific to hexaploid oat. (**) highlights translocation events (T6, T7) specific to *A.sativa sang* and missing in *A.insularis*. The other translocations occurred in the tetraploid ancestor of both species, an illustration of the events is provided in Extended Data Fig. 5a.

Label	Type	*A.insularis* [Mb]	*A.sativa sang* [Mb]
T1a	3D → 1C	0 – 39.22	0 – 40.05
T1b	1C → 3D	383.65 – 461.08	375.42 – 454.43
T2	C → 6D	0 – 40.44	(*)
T3	2C → 5D	421.05 – 481.35	427.65 – 489.89
T4	2D → 4C	0 – 71.83	0 – 67.01
T5	C → 4D	415.12 – 463.36	368.70 – 415.13
T6	1C → 1A		420.09 – 523.00 (**)
T7	T2(6D) → 2D		0 – 39.90 (**)

Supplementary Table 9: Differences in genome size and gene number between the extant hexaploid and the ancestral state predating the seven translocations between the tetraploid and hexaploid subgenomes.

		A	D	C
assembly size (Mb)	extant	3,277	3,125	3,868
	ancestral	3,171	3,005	4,094
	difference	106	121	-226
high confidence genes	extant	26,713	26,379	23,657
	ancestral	25,363	25,359	26,027
	difference	1,350	1,020	-2,370

Supplementary Table 10. **Multiplicity of oat gene families.** Number of orthologous gene families and genes as a function of their multiplicity (first column) on the A-, C-, and D-subgenomes (0: absent, 1: exactly one member and N: more than one member per subgenome). Multiplicities are not ordered for the three subgenomes. Columns 2 to 6 list the number of gene families and the number of genes mapping to the A-, C-, and D-subgenomes. The proportion of tandems in each group is shown in the seventh column, with groups significantly enriched for tandemly repeated genes in red. The last column counts the number of genomic bins (bin size 100 genes) with significant spatial clustering. 'total' summarises totals for the subgenome assignments of genes and gene families in extant oat (i.e., after occurrence of translocations), while 'ancestral' numbers are based on phylogenetic origin and ancestral subgenomic states (i.e., before occurrences of translocations in the tetraploid and hexaploid ancestors). (*) Note that gene families are based on orthologous inference while tandem genes are solely defined by their genomic neighbourhood and sequence similarity and hence also include distantly related paralogous copies. Further details are provided in Supplementary Methods.

Group	Families	A-genes	C-genes	D-genes	Total	Tandems*	Spatial
{1,1,1}	10,331	10,331	10,331	10,331	30,993	2.6%	0
{1,1,0}	4,513	3,549	2,221	3,256	9,026	5.6%	14
{0,1,N}	2,880	3,952	1,678	3,517	9,147	9.6%	200
{1,1,N}	1,079	1,498	1,556	1,498	4,552	16.4%	0
{1,N,N}	1,152	2,623	1,947	2,448	7,018	27.4%	0
{N,N,0}	585	1,547	544	1,394	3,485	41.3%	17
{N,N,N}	1,436	4,547	4,374	4,412	13,333	39.8%	0
{N,0,0}	1,090	708	1,109	649	2,466	19.4%	4
{1,0,0}	5,079	1,533	1,804	1,742	5,079	15.8%	0
**total**	**28,145**	**30,288**	**25,564**	**29,247**	**85,099**	**15.1%**	
**ancestral**	**28,145**	**28,802**	**28,016**	**28,281**	**85,099**	**15.1%**	

Supplementary Table 21: Hexaploid oat cv. Sang raw sequencing data.

Library type	Reads Length	Insert Size	Genomic Coverage
PCR-free	2x250bp	470	69
PCR-free	2x150bp	800	32
Mate-Pair	2x150bp	3000	41
Mate-Pair	2x150bp	6000	42
Mate-Pair	2x150bp	9000	42
Chromium	2x150bp	-	47

Supplementary Methods

Genome assembly of *A. sativa* cv. Sang

Hexaploid oat genomic DNA extraction, library preparation and sequencing

The cultivar 'Sang' was developed in Sweden by the Swedish Seed Association (Sveriges utsädesförening), and registered in 1974. Sang is a Spring oat with early maturation and excellent milling properties. Pedigree: (Sv 01771 x Sv 56697) x Condor.

High quality 25-50kb genomic DNA was isolated from etiolated leaves of the cv. Sang using the Qiagen DNeasy Plant Maxi kit, and five pairs of size-selected genomic DNA libraries were constructed, ranging from 450 bp to 10 kb (Supplementary Fig. 35a). Two shotgun libraries were prepared using 450-470bp size selected DNA fragments using the Hyper Kapa library preparation kit (Kapa Biosystems) without PCR amplification. Clustering was done by 'onboard clustering' and samples were sequenced on HiSeq2500 (HiSeq Control Software 2.2.58/RTA 1.18.64) with a 2x250 setup using 'HiSeq Rapid SBS Kit v2' chemistry. This fragment size was designed to produce a paired-end sequencing overlap of the fragments thus enabling 'stitching' of reads to approximately 470bp in length. Two libraries of 700-800bp DNA fragment sizes were made using the Hyper Kapa library preparation kit (Kapa Biosystems) with two cycles of PCR amplification. To increase sequence diversity and genome coverage, three pairs of Mate-Pair (MP) libraries were constructed with 2–4, 5–7, and 8–10 kb size ranges using the Illumina Nextera MP Sample Preparation Kit (Illumina, San Diego, CA). For the 700-800bp shotgun and MP libraries, clustering was done by 'cBot' and samples were sequenced on HiSeqX (HiSeq Control Software HD 3.4.0.38/RTA 2.7.7) with a 2x151 setup using 'HiSeq X SBS' chemistry.

Ultra-high molecular weight (uHMW) DNA was prepared using isolated nuclei from 5 g of etiolated leaves and following the standard CTAB method, except that cut tips were used to manipulate the sample and extreme care was taken to avoid mechanical agitation and freeze-thaw cycles. The size of the isolated DNA fragments was above 300kb as verified by pulsed-field gel electrophoresis (Supplementary Fig. 35b). The uHMW DNA fragments were used to construct two 10X Genomics Chromium genome libraries (10X Genomics, Pleasanton, CA). Clustering was done by 'cBot,' and samples were sequenced on HiSeqX (HiSeq Control Software HD 3.4.0.38/RTA 2.7.7) with a 2x151 setup using 'HiSeq X SBS' chemistry.

As detailed in Supplementary Table 21, the shotgun and MP libraries were used to generate 2,414Gb of sequencing data, equivalent to 226× genome depth (coverage), based on the assembled genome size of ~11Gb. The 10X Chromium library sequencing produced an additional 586Gb of sequencing data and 47× coverage, yielding a total coverage of 273×.

***A. sativa* genome assembly**

The sequencing data have been processed and assembled using the DeNovoMAGIC™ assembler application version 3.0 (NRGene, Nes Ziona, Israel) and the assembly statistics are summarised in Supplementary Table 1. DeNovoMAGIC™ is a DeBruijn-graph-based

assembler designed to extract the underlying information in the raw Illumina reads to solve the complexity of the DeBruijn graph due to genome polyploidy, heterozygosity, and repetitiveness. This is accomplished using accurate-reads-based traversing of the graph that iteratively connects consecutive phased contigs across local repeats to generate long phased scaffolds[58]. The 10X Chromium data were utilised to phase polyploidy/heterozygosity, support scaffold validation and further elongate the phased scaffolds.

In brief, the DeNovoMAGIC™ algorithm is composed of the following steps:

1. **Reads pre-processing.** PCR duplicates, Illumina adaptor AGATCGGAAGAGC and Nextera linkers (for MP libraries) were removed from the data. The 2x250bp overlapping reads from the 450–470 bp shotgun libraries were merged with a minimal required overlap of 10 bp to create the paired-end 'stitched' reads (PE reads).
2. **Error correction.** Following pre-processing, PE reads were scanned to detect and filter reads with a putative sequencing error (containing a subsequence that does not reappear several times in other reads).
3. **Contigs assembly.** The first step of the assembly involves the building of a De Bruijn graph (kmer=127 bp) of contigs from all the PE (including pre-processed 450–700 bp shotgun libraries) and MP reads. Next, PE reads were used to find reliable paths in the graph between contigs for repeat resolving and contigs extension. The 10X Chromium barcoded reads were mapped to contigs to ensure that adjacent contigs were connected only in cases in which those contigs originate from a single stretch of genomic sequence (reads from the same two or more barcodes were mapped to both contigs).
4. **Scaffold assembly.** Contigs were linked into scaffolds with PE and MP information, estimating gaps between the contigs according to the distance of PE and MP links. In addition, 10X Chromium data were used to validate and support correct phasing during scaffolding.
5. **Gap Filling.** A final gap filling step used PE and MP links and De Bruijn graph information to detect a unique path connecting the gap edges.
6. **Scaffold elongation and refinement.** 10X Chromium barcoded reads were mapped to the assembled scaffolds and clusters of reads with the same barcode mapped to adjacent contigs in the scaffolds were identified to be part of a single long molecule. Next, each scaffold was scanned with a 20 kb window to ensure that the number of distinct clusters that cover the entire window (indicating a support for this 20 kb connection by several long molecules) was statistically significant with respect to the number of clusters that span the left and the right edge of the window. In cases where a potential scaffold assembly error was detected the scaffold was broken at the two edges of the suspect 20 kb window. Finally, barcodes that were mapped to scaffold edges were compared (first and last 20kb sequences) to generate a scaffold graph with a link connecting two scaffolds with more than two common barcodes. Linear scaffold paths in the scaffolds graph were composed into the final scaffold output of the assembly.

Chromosome conformation capture sequencing

Chromosome conformation capture sequencing (Hi-C) data was generated using the protocol of Padmarasu et al. (2019)[59].

Pseudomolecule construction for *A. sativa* cv. Sang

Pseudomolecule construction for *A. sativa* cv. Sang was done with the TRITEX pipeline protocol[8]. The NRGene assembly of *A. sativa* cv. Sang was digested *in silico* with DpnII using EMBOSS restrict[60]. Hi-C and 10X linked-read data were aligned to the NRGene assembly with Minimap2[61]. Alignment records were converted to binary Sequence Alignment/Map format using SAMtools[62] and sorted with Novosort (http://www.novocraft.com/products/novosort/). A list of Hi-C links was extracted from Hi-C alignments using BEDTools[63] and TRITEX scripts. Linked-read alignment was aggregated into molecules using BEDTools[63] and TRITEX scripts (https://bitbucket.org/tritexassembly/tritexassembly.bitbucket.io). Hi-C links, 10X molecules, and guide map alignments were imported to the R statistical environment [64] and analyzed further with TRITEX scripts. Breakpoints in chimeric scaffolds joining unlinked sequences were detected as drops in physical coverage with Hi-C links and/or 10X molecules. An initial Hi-C map was generated using the minimum spanning tree algorithm described by Beier et al. (2017)[65]. The assembly and Hi-C map were iteratively corrected by inspecting Hi-C contact matrices, guide map alignments and physical coverage with 10X and Hi-C reads. Sequence files in FASTA format and AGP tables for pseudomolecules were compiled using TRITEX scripts. The pseudomolecules of cv. Sang were aligned against the pseudomolecules constructed from a long-read sequence assembly of cv. OT3098. The OT3098 pseudomolecules were downloaded from GrainGenes[54].

Genome assembly of *Avena insularis* Durieu and *Avena longiglumis* Ladizinsky sp. nov

Plant Material and DNA extraction

For whole-genome assembly, single plants from *A. insularis* (BYU209; Ladizinsky (1998); Sicily, Italy[12]) and *A. longiglumis* (CN58138; Oran, Algeria) were grown hydroponically in an isolated growth chamber under a 12-h photoperiod. Growing temperatures ranged from 18°C (night) to 20°C (day). The hydroponic growth solution was made using MaxiBloom® Hydroponics Plant Food (General Hydroponics, Sevastopol, CA, USA) at a concentration of 1.7 g/L. BYU209 and CN58138 are publicly available and maintained in germplasm collections at Brigham Young University (Provo, Utah, USA) and the National Plant Genebank (PGRC, Saskatoon, Saskatchewan, CA), respectively.

DNA sequencing, primary assembly and polishing

In preparation for PacBio CLR sequencing, high molecular weight DNA was extracted from 72-h dark-treated leaf samples using a CTAB-Qiagen Genomic-tip protocol[66]. For whole-genome sequencing, large-insert SMRTBell libraries (> 20 kb), selected using a SageElf (Sage Science, Inc., Beverly, MA, USA), were prepared according to standard manufacture protocols and sequenced at the BYU DNA Sequencing Center (Provo, UT, USA) using P6-C4 chemistry on a Sequel II instrument (Pacific BioSciences, Menlo Park, CA, USA). For whole genome polishing, DNA for each species was sent to Genome Quebec (Montreal, Quebec, CA) for NovaSeq (2X 150-bp paired-end) sequencing from standard 500-bp insert libraries. Trimmomatic v0.35[67] was used to remove adapter sequences, leading and trailing bases with a quality score below 20 or average per-base quality of 20 over a 4-nucleotide sliding window. After trimming, any reads shorter than 75 nucleotides in length were removed. A primary contig assembly for both *A. insularis* and *A. longiglumis* (Supplementary Table 18) was

constructed with using Canu v1.9[68] with default parameters with the following flags: corOutCoverage = 40, correctedErrorRate=0.035, utgOvlErrorRate=0.065, trimReadsCoverage=2, and trimReadsOverlap=500. The primary assembly was polished twice using Arrow from the GenomicConsensus package in the Pacific BioSciences SMRT portal v5.1.0 followed by a single round of insertion/deletion correction detected by the Illumina reads using PILON v0.22[69].

Chromosome conformation capture sequencing
This sequencing was performed as for cv. Sang.

Pseudomolecule construction for *A. longiglumis* CN58138 and *A. insularis* BYU209
Pseudomolecule construction for *A. longiglumis* CN58138 and *A. insularis* BYU209 was done as described above for *A. sativa* cv. Sang with some modifications. The order of single-copy sequence (≥ 1 kb in length) in the pseudomolecule of *A. sativa* cv. Sang was used as a guide map. Single-copy sequences were extracted using BBDuk (https://jgi.doe.gov/data-and-tools/software-tools/bbtools/bb-tools-user-guide/) and TRITEX scripts (https://bitbucket.org/tritexassembly/tritexassembly.bitbucket.io/src/master/miscellaneous/mask_assembly.zsh), and aligned to the Canu assemblies of both species using Minimap2[61]. Hi-C data were used for chimera detection and pseudomolecule construction. After iterative correction and refinement of the Hi-C map based on inspection of contact matrices, pseudomolecules were compiled using TRITEX scripts.

Whole Genome Alignments (WGAs) between the *Avena* reference assemblies
A whole genome alignment (WGA) between *A. sativa*, *A. insularis*, *A. longiglumis*, *A. eriantha* and *A. atlantica* was generated using the cactus pipeline version 1.0[70]. Prior to the alignment step, all nucleotide sequences were 20-kmer-softmasked to reduce complexity and facilitate construction of the WGA using the tallymer subtools from the genome tools package version 1.6.1[71]. The pipeline was run stepwise with default settings described at https://github.com/ComparativeGenomicsToolkit/cactus#running-step-by-step. Dot plots of pairwise alignments were made using the supplied scripts by the cactus package.

Synteny framework and genome assembly comparison between *A. sativa* cv. Sang and OT3098
Synteny between the two oat genome assemblies was established using an all-against-all blastp matrix restricted to the top ten hits for each query and McScanX with default parameters on the 21 chromosomes. Likewise, the full protein similarity matrix with a minimal expectation value (e <= 10^{-10}) was surveyed to identify reciprocal or bidirectional best blast hits (BBH). Multiple collinear assignments - which are common for a hexaploid, were resolved selecting the highest scoring alignment. Only syntenic segments with a minimum number of 10 gene pairs were reported. Results were visualised and analysed with two different dot plot schemes, one using the actual physical gene position as axis locations (method dotA), and a second enumerating gene order along both axes (method dotB). Computed borders between collinear segments including inversions and low gene density regions were manually evaluated and - if necessary, refined in interactive dot plots. Using the resulting gene-based, aligned coordinate

system of the collinear parallel and anti-parallel segments, inversions between the OT3098 and Sang assemblies were identified from the endpoints of each segment. All reported inversions were additionally inspected and confirmed. To identify regions of unbalanced/reduced gene densities, we computed for each segment with sliding overlapping windows (window size of 20 and window steps of 10 syntenic genes) their slopes using the dotB coordinate system. Elevated slopes approximated very well the boundaries of these regions and were subsequently manually refined to identify inflection points of the curve. We restricted our report to regions with increased slope values containing at least 50 OT3098 genes (syntenic and non-syntenic). The results of these analyses are available as Supplementary Tables 2 and 7. We further associated the results from the unbalanced/reduced gene density and inversions/translocations analyses (in the form of locations with coordinates and unique identifiers per region/inversion) with the orthologous gene framework between the Sang and the OT3098 assemblies (described above). The resulting association table is provided with the Sang gene annotation.

Hexaploid oat transcriptome atlas

Plant Material and Tissue

An overview of the tissue samples and developmental stages used in this work, including the Zadoks decimal stage estimate, is provided in Supplementary Table 20. Oat plants were grown in pots under long-day conditions (18 h day) in a climate chamber with day/night temperature of 20/18°C and light photo flux density of 240 µmol/m^2/s. Tissue was collected from developing seed, leaf, root, crown and stem. Samples from seed development included three time points collected during the day or night: Early (4 dpa = seed green, densely covered with trichomes, not fully elongated, endosperm not filling); Mid (14 dpa = seed green, trichomes dense at top only, fully elongated, milky white endosperm); Late (20 dpa = seed mostly pale yellow, fully elongated, trichomes dense at top only, soft dough endosperm. Samples were collected during the same time of day and were frozen in liquid nitrogen immediately upon collection. Total RNA isolation was performed using the Qiagen RNeasy plant Mini kit following the manufacturer's instructions. A specification of each sample used in annotation, network analysis and single-gene mapping, and the additional tissue samples used in this work from publicly available data are outlined in Supplementary Table 20.

Library preparation and Sequencing

The methods used for developing seed samples are detailed here (for details pertaining to all samples see Supplementary Table 20). RNA concentration was measured with a Qubit 3.0 Fluorometer (Thermo Fisher Scientific) and RNA quality was evaluated using the Caliper LabChip GX Nucleic Acid Analyzer (PerkinElmer). The Illumina protocol (TruSeq Stranded mRNA Reference Guide, 1000000040498 v00) was adapted to be run on an Agilent BRAVO robot. In brief, mRNA was isolated from total RNA using poly dT-coated beads and then sheared to 150-400 bp fragments through chemical fragmentation. The mRNA was converted into cDNA using reverse transcriptase, and a clean-up step using AMPure XP beads was used to remove enzymes and short fragments. Next the fragments were adenylated (a single adenosine is added to the 3' end of the blunt fragments which prevents self-ligation during the adapter ligation step) followed by ligation of adapters and a clean-up step (AMPure XP beads) to remove the enzymes and unligated adapters. This was followed by PCR amplification and a

final PCR clean-up step (AMPure XP beads). Libraries were sequenced on a Illumina HiSeq 2500 (HiSeq Control Software 2.2.58/RTA 1.18.64) with a 2x126 setup using 'HiSeq SBS Kit v4' chemistry. The Bcl to FastQ conversion was performed using bcl2fastq from the CASAVA software suite. The quality scale used was Sanger / phred33 / Illumina 1.8+.

Quality control and read trimming

The RNA-seq reads used for genome annotation (Supplementary Table 19, datasets: a-c,k-r) were trimmed using Trimmomatic[67] (-phred33 LEADING:30 TRAILING:30 SLIDINGWINDOW:4:30 MINLEN:40). Quality control was performed with FASTQC (v0.11.8) before and after trimming.

Annotation of protein coding genes

For the annotation of protein coding genes, evidence from transcriptome data as well as homology information from related species were incorporated in a first step. For the homology-based annotation, protein sequences from Uniprot of *Arabidopsis thaliana* (Tair10)*, Sorghum bicolor v3.2, Hordeum vulgare* (IBSC v2*), Brachypodium dystachion* (v3.0)*,* and *Oryza sativa* (IRGSP-1.0) (downloaded on April 18th 2019) were combined into a database (AnnoDB) and mapped to the *A. sativa* cv. Sang reference genome sequence v1 using the splice-aware mapper GenomeThreader[72] (version 1.7.1, parameters: -startcodon -finalstopcodon -species rice -gcmincoverage 70 -prseedlength 7 -prhdist 4).

In the evidence-based step, multiple RNA-seq datasets (Supplementary Table 19, datasets: a,b,c,k-r) were used for the genome-guided prediction of gene structures. RNA-seq data were mapped to the genome reference with HISAT2[73] (version 2.1.0, parameter –dta) and assembled to transcripts with Stringtie[74] (version 1.2.3, parameters -m 150 -t -f 0.3). Transdecoder (version 3.0.0) (https://github.com/TransDecoder/TransDecoder) was used to identify potential open reading frames and predict protein sequences. The predicted protein sequences were compared against a protein reference database (UniProt Magnoliophyta, reviewed/Swiss-Prot) using BLASTP[75] (-max_target_seqs 1 -evalue 1e-05) and hmmscan version 3.1b2[76] was used to identify conserved protein family domains for all proteins. BLAST and hmmscan results were then processed by Transdecoder-predict and the best translation per transcript sequence was selected. Finally, results from the two gene prediction approaches were combined and redundant protein sequences were removed. An intermediate step of confidence classification was performed next. Protein sequences with an open reading frame were extracted from the annotation using gffread[77] (v0.11.6) and Biopython[78]. Diamond[79] (v0.9.29.130, e-value 1) was used to compare these to AnnoDB, and a database containing validated magnoliophyta proteins (UniMag) downloaded from Uniprot on 2017-02-20, and PTREP (release 16[80]), a database that contains hypothetical proteins with deduced amino acid sequences where in many cases, frameshifts have been omitted, and is useful for the identification of divergent TEs without significant similarity at the DNA sequence level. The two former databases were filtered for sequences containing both start and stop codons. Hits with a subject and query coverage above 80% were selected and protein sequences were further classified into high and low confidence. High confidence (HC) hits were complete and had a subject- and query-coverage above the threshold in the UniMag database or no blast hit in UniMag but in AnnoDB and not in PTREP.

A low confidence (LC) hit on the other hand is not complete and has a hit in the UniMag or AnnoDB database but not in PTREP, or no hit in UniMag and AnnoDB and PTREP but the protein sequence is incomplete. This pipeline is available at https://github.com/PGSB-HMGU/plant.annot.git.

Next, a genome-guided transcriptome assembly was performed using Trinity[81] (v2.8.5, options --genome_guided_max_intron 30000) with the same RNA-seq data as above aligned using STAR[82] (v2.7.6a, options --outSAMtype BAM SortedByCoordinate --alignIntronMax 50000 --outSAMstrandField intronMotif) as input. PASApipeline[83] v2.3.3 was run on the Trinity assembly. GMAP[84] v2017.11.15 was used for transcript alignment as a part of the PASApipeline run. The script process_GMAP_alignments_gff3_chimeras_ok.pl was updated to run gmapl instead of gmap to handle the large genome size.

TransDecoder (https://github.com/TransDecoder/TransDecoder, v5.5.0) was used to identify coding regions in the spliced alignment produced by PASApipeline. To help this prediction, Diamond[79] v0.9.25 (options --more-sensitive --max-target-seqs 1 --evalue 1e-5) and HMMER[85] v3.2.1 (options --domtblout) were used to perform homology search in Uniref90[86], downloaded on 2019-09-03 and Pfam[87], downloaded on 2019-09-03 respectively. The identified coding regions were mapped back to the reference genome using the script provided as a part of TransDecoder.

A first AUGUSTUS[88] (v3.3.1, options --UTR=on --alternatives-from-evidence=true --allow_hinted_splicesites=atac) prediction was done using the wheat parameters. To avoid potential over-prediction, guiding hints were generated using transcriptome data, predicted protein sequences of HC genes, and TE-hints. Retraining AUGUSTUS was done using all 100% supported, non-redundant transcripts that did not match the TEs in the Hypothetical TREP protein sequences database PTREP [80], release 19, using the flanking region computed by the script provided as a part of AUGUSTUS and the standard initialization, with 1000 sequences used for evaluation during training and 500 sequences used for final performance testing, following the steps provided by Hoff and Stanke (2019)[88]. AUGUSTUS (v3.3.3, options --UTR=off --alternatives-from-evidence=true --allow_hinted_splicesites=atac) was used to predict genes using the new oat parameters.

EVidenceModeler[89] (EVM, git commit 73350ce, partition options --segmentSize 900000 --overlapSize 50000) was used to merge predictions and alignments from the non-redundant annotation based on the homology information and the Stringtie transcripts, PASA pipeline, and AUGUSTUS. A constraint on the output path in the EVM script was removed to allow for processing multiple weight settings in parallel. All inputs were provided as gene predictions to EVM, and all inputs except for AUGUSTUS were also provided as protein alignment evidence to EVM. Evaluation of the different weight settings was performed using BUSCO[90], v3.0.2, liliopsida_odb10 created on 2017-12-01, and embryophyta_odb9 created on 2017-02-13, protein mode). BUSCO statistics served as the main guide for choosing which EVM weights to use.

PASApipeline[83] v2.4.1 was used to update the annotation resulting from EVM to add UTRs. This was done by loading the EVM annotation into a PASA pipeline database, and running two rounds of updates.

Confidence classification was done on the final PASA pipeline output as described above. Several coverage thresholds were evaluated based on BUSCO results (v4.0.4, liliopsida_odb10 created on 2019-11-20, protein mode) and blastn[75] (v2.9.0+, options -max_target_seqs 1 -evalue 1e-10) hits against Iso-Seq transcripts, with a threshold of 65% set for both query and subject coverage across all three databases. Predicted proteins were classified as described previously [7].

Representative transcripts for genes with several isoforms were selected by performing a blast all vs. all search with blastp[75] against AnnoDB. The best transcript was chosen based on coverage and identity. If no hit was found against the database the longest transcript was selected.

Additionally, a few genes, including *CslF6* and several prolamins and avenins, were curated manually, with most being integrated into version 1.1 of the gene annotation. Manual curation was based on identifying previously published sequences of the genes of interest, and building multiple sequence alignments (MSAs) of these and our candidate genes. The MSAs were reviewed together with transcript evidence to improve the gene annotations.

Functional annotation of transcripts as well as the assignment of GO terms, and Pfam- and InterPro-domains were performed with the tool AHRD (Automatic assignment of Human Readable Descriptions, https://github.com/groupschoof/AHRD; version 3.3.3). AHRD performs a BLASTP search against the databases Swiss-Prot, The Arabidopsis Information Resource (TAIR), and TrEMBL to assess homology information to other known proteins. Functional annotations are defined based on the homology information and domain search results from InterProScan and Gene Ontology (GO) terms are integrated[91].

Annotation of the *A. insularis* and *A. longiglumis genome sequences*

Structural gene annotations for *A. insularis* and *A. longiglumis* were done using a lift-over approach. The method is described in detail in Hoff and Stanke (2019)[92]. Briefly, genes from each individual *A. sativa* subgenome and the previously published *A. eriantha* and *A. atlantica* genomes[14] were lifted to the respective progenitor genome sequences as follows:

- *A. sativa* D gene models were lifted to *A. insularis* D, *A. sativa* C and *A. eriantha* gene models were lifted to *A. insularis* C and *A. sativa* A and *A. atlantica* gene models were lifted to *A. longiglumis*.
- To minimise annotation artefacts and archive maximum uniformity between all genome annotations, *de novo* gene models from *A. sativa* A and C were additionally lifted to *A. atlantica* and *A. eriantha*, respectively.
- Lifted annotations were merged if necessary and all annotations were subjected to the confidence classification pipeline detailed in the *A. sativa de novo* annotation section.

Repeat annotation

Identification and clustering of tandem repeats

Tandem repeats consist of monomer sequence units arranged directly next to each other. For a simple and pragmatic classification they are commonly split into three main categories by their unit length: 1-9 bp minisatellites, 10-99 bp microsatellites and ≥ 100 bp satellites. In the oat assemblies tandem repeats were annotated with TandemRepeatsFinder v 4.07b under default parameters (Match Mismatch Delta PM PI Minscore MaxPeriod: 2 7 7 80 10 50 500)[93]. Overlapping annotations were removed with a priority based approach assigning higher scoring and longer elements first. Elements which overlapped already assigned elements were either discarded (> 90% overlap) or shortened (≤ 90% overlap) if their rest length exceeded 49 bp.

To obtain a collection of non-redundant tandem repeat sequences the consensus sequences of the tandem repeat units (output of TandemRepeatsFinder) were clustered with vmatch dbcluster under stringent conditions into subfamilies (≥ 98% identity and a mutual overlap ≥98%, command line: vmatch dbcluster 98 98 -v -identity 98 -exdrop 3 -seedlength 20 -d -p) (http://www.vmatch.de). For each of the 300 largest tandem repeat clusters the chromosomal location of their members was plotted along the chromosomes. Only 10% of the tandem repeat subfamilies were more or less evenly distributed across all subgenomes, 52 % showed a strong enrichment for the A and D subgenome , 36% were C, 2% A and 1 % CD enriched. Some of the enrichments showed an almost 100% specificity for the ancestral subgenomes, examples are given in Extended Data Fig. 1c. Centromere located and mostly subgenome specific tandem repeat subfamilies could also be identified (11 subgenome specific centromere clusters for the D subgenome, four for C and one unspecific occurring on A, D and C). A comparison of the tandem repeat composition between the short read Sang assembly and the long read OT3098 assembly is given in Supplementary Table 6.

Transposon annotation by homology to a TE-library

Transposons were detected and classified in the hexaploid oat cv Sang and var. OT3098 assemblies by a homology search against the REdat_9.9_Poaceae section of the PGSB transposon library[94]. The TE-library had beforehand been filled up with 744 de novo detected high quality full length LTR-retrotransposon template sequences which occurred at least two times as complete element in the assembly as determined by the pragmatic "80/80 clustering" (≥ 80% identity, ≥ 80% mutual sequence coverage).

The program vmatch (http://www.vmatch.de) was used for the homology search as a fast and efficient matching tool that is well suited for large and highly repetitive genomes. Vmatch was run with the following parameters: identity ≥ 70%, minimal hit length 75 bp, seedlength 12 bp (exact command-line: -d -p -l 75 -identity 70 -seedlength 12 -exdrop 5). To remove overlapping annotations the vmatch output was filtered for redundant hits via a priority based approach. Higher scoring matches were assigned first and lower scoring hits at overlapping positions were either shortened or removed if they were contained to ≥ 90% in the overlap or if <50 bp of rest length remained. The resulting transposon annotation is overlap free, although disrupted elements from nested insertions have not been de-fragmented into one element. With the

described approach the TE content presently annotated by homology amounts to 63.8% divided into 61.7% retrotransposons and 1.8% (still underrepresented) DNA-transposons in both assemblies.

Genome structure, rearrangements and translocations

Chromosome nomenclature

We assigned a novel nomenclature to the oat chromosomes by inspecting the gene-based collinearity to barley and orientations of core region gene sets.

Aligning the 6k markers

Manifest sequences of the markers[51] were aligned to the pseudomolecules using gmap[84] v2020.06.01. Only markers aligning perfectly were kept. In cases where a marker was aligning in multiple locations, but not to chrUn, and only once to the chromosome corresponding to the merge group given in the consensus map[10], this location was used. Markers not aligning perfectly, or aligning in multiple locations but in a way that could not be resolved by the consensus map, were discarded. This analysis was done using an R script (v4.1.0, tidyverse v1.3.1 , ggplot2[95] v3.3.5).

Recombination frequency (r)

Marker data were analysed from 5 bi-parental populations of recombinant inbred lines (RILs) which were assayed using genotyping by sequencing (GBS) following detailed methods described in a companion paper[21]. Briefly, raw GBS sequence data from FASTQ files were re-analyzed using Haplotag software[96] and FSHap as implemented in TASSEL[97] v5.0 following methods for the Haplotag production pipeline described by Bekele et al. (2018)[10]. 64bp tag-level haplotypes of all filtered GBS loci were matched to the Sang genome, and only those with a perfect 64 bp match of a single haplotype to a single unique genome location were kept for further analysis.

Marker data for each of the five populations were then ordered based on pseudomolecule position of the corresponding GBS tags in Sang, and re-analyzed to impute missing genotypes, remove improbable double-crossovers, and correct miss-called heterozygotes within runs of heterozygosity using an in-house algorithm (presented and evaluated by Tinker et al., In Press[21]).

To compute global crossover frequency on a linear scale, a genome-wide map of crossover locations in each RIL progeny was computed. A single crossover was inferred at a random position between two markers with different homozygous states, and 0.5 crossovers were inferred if one allele state was heterozygous. Crossovers were then counted in windows of 20 Mbp (up to 10 Mbp left or right, where possible) at each 1 Mbp increment of each full pseudomolecule. The crossover rate at each 1Mbp increment was expressed as the crossover frequency per progeny per 100Mb.

Recombination matrices among different parts of the genome were computed and examined separately for two of the five RIL populations. The sorted and imputed genotype matrix was used to compute average pairwise recombination frequencies between all possible 16 Mbp windows at 1 Mbp increments. Within each pair of windows, r was computed as the arithmetic

mean of the recombination rate between all possible marker pairs in the corresponding two windows. If no marker pairs were available within a given window, the nearest pair of markers to the start and end of the window were used to represent the window. Recombination matrices within and between chromosomes were visualised in two dimensions using a heat map of blended colours representing different average recombination frequencies.

Subgenome-specific kmers

To compute kmer sets with an enriched specificity for either the A-, D- or C-subgenome, we employed whole genome sequence information of the A- (*A. longiglumis*) and C-lineage (*A. eriantha*) diploids and the DC-tetraploid *A. insularis*. KMC tools[98] v3 was used to both determine kmer (K=27) counts and occurrences and to perform the set operations union, intersection and difference in the respective kmer sets. For each analysis, we distinguished between the source genomes/species that provide the kmer sequences and the subgenome labels and the target genome/species to which the source kmers are positioned using vmatch[99]; www.vmatch.de). For each source kmer set S, we counted canonical kmers in the source genome and selected unique kmers after applying the set operations to query their positions in the target genome. To reconstruct the subgenomic ancestry in oat by kmers, we conducted a two-step process. First we analysed the genomic distribution of A- and C-specific kmers in *A. insularis* to identify potential translocations that have occurred in the tetraploid ancestor of oat. The subgenome specific kmers were detected as

$$A_{ins} = (S_{ins} - S_{eri}) \cap S_{lon} \wedge C_{ins} = (S_{ins} - S_{lon}) \cap S_{eri},$$

where A_{ins} and C_{ins} are the A- and C-specific kmer sets, and S_{ins}, S_{lon} and S_{eri} are the total source kmers of *A. insularis*, *A. longiglumis* and *A. eriantha*, respectively.

A total of 198,625,685 and 260,894,419 kmers were found for C_{ins} and A_{ins}, respectively. Subgenomic kmers were mapped to the tetraploid genome, binned into adjacent sets of 2,000 kmers by their genomic position, and sliding windows with an overlap of 1,000 kmers were tested for their A- or C-ancestry using a binomial test with an event probability adjusted for the relative frequencies of A- and C-specific kmers. To reduce noise within the data and to target large-scale translocation in *A. insularis* and *A.sativa,* we employed a one-dimensional Gaussian kernel filter to the likelihoods of the genomic kmer bins (Supplementary Fig. 10 and Extended Data Fig. 4a).

Based on the segmentation of the A. insularis genome described above, we identified A-, C- and D-specific kmer sets using the following set operation schema:

$$A_{sat} = (S_{sat} - (C_{ins} \cup D_{ins})) \cap S_{lon},$$

where S_{sat} and S_{lon} are the total kmers in A. sativa and *A. longiglumis*, A_{sat} represents the A-specific query kmers, and C_{ins} and D_{ins} are the previously identified D- and C-specific kmers in A.insularis. Analogous operations were defined for D_{sat} and C_{sat}, the D- and C-specific query

kmers for oat. Due to the close relationship between the A- and D-lineages, the number of A- (~202×10^6) and D-specific kmers (~452×10^6) was considerably lower than the C-specific total (~845×10^6). The assignment of genomic regions to one of the three subgenomes in oat was performed similar to the approach described for *A. insularis,* but using bins of 5,000 adjacent kmers and three tests for each possible kmer set. As expected for their higher similarity, A and D subgenomic regions were less distinguishable than comparisons with the C-subgenome. However, for all translocations reported in this study, subgenomic assignments were unambiguous.

Syntenic Blocks

Syntenic orthologous and homoeologous blocks of spatially correlated genes within all seven *Avena* species and subgenomes were computed from a high quality ortholog set that has been derived from parsing the gene trees of the orthofinder analysis described below. Requiring a monophyletic origin for the *Avena* genes and exactly one *B. distachyon* ortholog as outgroup, we identified 9,253 gene families with exactly one gene copy in each of the selected diploids (*A. longiglumis* and *A. eriantha*), two copies in the tetraploid *A. insularis* and three copies in hexaploid oat. These selected gene families were also the basis of the triad analysis in "Definition of triads" under "Gene expression analysis" in the following text. Importantly, we did not force any multiplicity on the tetra- and hexaploid families but intentionally included {0,1,2}-groups. Collinearity of orthologous genes between species is widely used as strong evidence for common ancestry. However, even high quality assemblies contain a small proportion of erroneously oriented scaffolds, and in multiple species comparisons, already one such scaffold can impair collinearity and synteny analysis. To minimise these effects, and given the expected dynamics of genomic rearrangements in the *Avena* clade, we defined syntenic blocks that did not require a strict collinear order but at least 20 (orthologous respectively homoeologous) genes per block congruently located in close genomic proximity in all seven species or subgenomes. First, we transformed the genomic position of each ortholog/homoeolog to an ordinal position i=1,2,…N (where N is the total number of selected genes on the respective chromosome) and normalised its index by the total N. Next, we generated for each of the 9,253 genegroups a feature vector V obeying a gene order A. *eriantha, A. longiglumis*, two copies of *A. insularis* and three copies of *A. sativa*. The distance between two feature vectors V^i, V^j is calculated by the following rules:

1. Only columns of the same species are considered.
2. $d(V^i,V^j) = 1$ if the paired genes are on distinct contigs.
3. $d(V^i,V^j) = |P^i - P^j|$, where P^i, P^j indicate relative position of gene pair i,j.
4. Out of the four and nine possible comparisons for the tetra- and hexaploid case, the two and three lowest distances are selected
5. The total distance is the sum of all seven pairwise distances obtained from (i) to (iv).

The resulting distance matrix of 9,253×9,253 pairwise distances was transformed into an undirected graph with a genegroup as node and edges between nodes with a maximal distance of 0.2. Lastly, a community clustering using the Louvain algorithm (https://github.com/taynaud/python-louvain) was performed and clusters with less than 20

genes per diploid species were discarded. In total, 357 blocks, 51 per species or subgenome - were detected with a mean size of 65 Mb and a mean gene number of 174 genes (Supplementary Fig. 36). Out of 9,253 gene families, 8,855 (95.7%) were assigned to one of the syntenic blocks.

Gene family analysis

Orthofinder

We identified gene families in oat by a genome-wide phylogenetic comparison of the *A. sativa* proteome to a representative set of 10 *Poaceae* species with an emphasis on the *Triticeae* clade. This set comprised *Z. mays* (B73v5), *O. sativa* (IRGSP-1.0), *B. distachyon* (v3.2), *H. vulgare* (Morex v3), *S. cereale* (IRGSC Lo7 v3), hexaploid bread wheat (*T. aestivum*; IWGSC v1.1), tetraploid *A. insularis*, and three diploid species, *A. longiglumis* and *A. atlantica* as A-lineage and *A.eriantha* as C-lineage representatives. For the *Avena* species, both low- and high-confidence genes were included in the phylogenies. For the others with multiple splice variants either available representative transcripts were used or the longest transcript was selected. Using *Arabidopsis thaliana* (Araport 11) as an additional outgroup, we employed Orthofinder[100] v2.4 to extract orthologs and co-orthologs between the *Poaceae* species and grouped them into gene families.

Definition of gene multiplicity and tandem genes

From the Orthofinder results described above, only families containing at least one *A. sativa* gene were retained. Next, we filtered out all singleton LC genes and groups consisting entirely of LC genes and for which at least one gene had a transposon signature. Lastly, HC genes assigned as singletons in the orthofinder run were added to derive the final reported number of 31,219 oat gene families comprising a total of 107,495 LC and HC genes. We denote that these families might contain a small number of closely related out-paralogs that were not sufficiently resolved by the phylogenetic analysis. However, given the distribution of cluster sizes (Supplementary Figure 11) and the vast majority (>93%) of highly confined families with ≤ 6 genes, putative out-paralogous relationships play only a very minor role.

To analyze subgenomic multiplicity, we confined the above data set to those 28,145 families for which all genes in one family had a location on one of the 21 oat chromosomes, thereby omitting clusters for which at least one gene was located on unanchored scaffolds ('chromosome unknown'). Spatial clustering of a particular class of subgenomic multiplicity was assessed for gene bins of size 100 by a hypergeometric test

$$P(X \geq k) = \sum_{i=k}^{K} f(i; N; K; 100); with f(i; N, K; n) = \frac{\binom{K}{k}\binom{N-K}{100-k}}{\binom{N}{100}}$$

where N is the total number of analysed genes (85,099), K is the total number of genes, and k is the number of genes in the respective bin belonging to the tested class of multiplicity. In practice, we applied the cumulative density function (CDF) of the hypergeometric distribution

as implemented in SciPy[101] v1.6.1. Adjacent sliding gene bins overlapped by 50 genes. Reported probabilities were Bonferroni corrected.

In contrast to gene families, tandem genes were not phylogenetically defined but by their sequence homology and genomic proximity. Sequence similarities were derived from an all vs. all blastn comparisons of oat coding sequences of the 28,145 gene families used for the multiplicity analysis and applying a minimum e-value threshold $\leq 10^{-30}$. To filter for gene neighbourhood, we constructed an undirected graph with homologous genes as nodes (see above) and inserted edges between genes if at most nine unrelated (i.e. non-similar) genes were located between them. Tandem clusters of size ≥ 2 genes were subsequently retrieved as connected components using networkx v2.5 (https://networkx.org). A total of 5,432 tandem clusters comprising 14,776 genes were detected.

Identification of the cellulose and callose synthase gene families

Protein sequences of *A. sativa* were used to identify cellulose, cellulose-like and callose synthase genes using a set of reference genes comprised of genes retrieved by exploiting publicly available reference sequences[102–104] as well as of gene retrieved from UniProtKB and filtered for manually annotated records (Supplementary Table 22). Reference protein sequences were mapped against *A. sativa* cv. Sang protein sequences (representative transcripts) using blastp[75] (-max_hsps 1 -evalue 1e-2 -use_sw_tback).

The domain structure of reference sequences was determined through InterProScan5[105] (-iprlookup -goterms -pa -f TSV -dp -appl Pfam,TIGRFAM,SUPERFAMIL). The domain structure of *A. sativa* genes was used from the AHRD result. A gene was considered a hit when the oat protein domain structure overlapped with the domain structure of the reference sequence and 60% of each of the sequences was included in the alignment.

In addition, missed sequences that had the PF03552 and PF00535 protein domains were added to the oat set of candidate genes.The resulting set of oat candidate genes was compared to the Orthofinder result and potentially missed orthologs were included. In an additional iteration step, the set of oat candidate genes was used to search for candidates in the *A. sativa* protein set again. Moreover, this set was mapped to *A. sativa* cv. Sang and OT3098 genomes with tblastn[75] to search for genes that were not annotated. This revealed no hits.

Investigation of gene family expansion in *CesA*, *Csl* and callose synthase gene families in oat

After identifying the cellulose synthase superfamily genes in *Avena sativa*, these were compared between *Avena sativa* and other species to investigate potential expansions and/or contractions that may be relevant to the elevated beta-glucan content of oats. An Orthofinder analysis was conducted as the basis for in-depth gene family analysis. In order to assign cellulose synthase gene superfamily members to the cellulose synthase (*CesA*) subfamily and the seven cellulose synthase-like subfamilies, *CslA, CslC, CslD, CslE, CslF, CslH,* and *CslJ* homology information from other species (e.g. rice, *Arabidopsis*) was used. A multiple

sequence alignment of all sequences belonging to the cellulose gene superfamily was calculated using MUSCLE[106].

Identification and characterization of storage protein gene models

Prolamin gene models were identified using avenin sequences retrieved from UniProt Knowledgebase appended with avenin transcripts identified by Tanner et al. (2019)[107]. Pfam domain search was performed using profile Hidden Markov models (HMMER3) to identify sequences with HMW glutenin (PF03157), Gliadin (PF13016) and Tryp_alpha_amyl domains (PF00234) as described by Juhász et al. (2018)[37].

Additionally, globulins possessing cupin_1 domain (PF00190) were identified. Sequences were aligned with public avenin and ATI sequences and protein sub-groups were defined. For globulins, the identified gene models were aligned to 11S-, 7S-globulins and globulin-1 sequences from wheat.

To compare protein size and N storage capacity of the storage protein sequences of oats to other crop species, prolamin and globulin protein sequences of wheat (*Triticum aestivum* IWGSC RefSeq v1.1), rice (*Oryza sativa* Japonica Group IRGSP-1.0) and soybean (*Glycine max* v2.1) were retrieved from Ensembl Plants. Protein sequences were aligned using ClustalW and protein subtypes have been identified. Amino acid composition of the identified gene models was determined in CLC Genomics Workbench v21 (Qiagen, Aarhus, Denmark). The protein length normalised sum of asparagine and glutamine residue counts was used to compare N storage capacity. Data was visualised using custom R[64] packages, ggplot2[108] and ggpubr[109].

Epitope mapping and phylogenetic analysis

The predicted avenin and ATI protein sequences from each of the oat sub-genomes were combined with the predicted gliadin, glutenin, hordein and ATI protein sequences from *Hordeum vulgare subsp. vulgare* cv. Morex genome assembly v3 and *Triticum aestivum* assembly v1 annotation v1.1) for phylogenomic analysis. The oat sub-genomes were handled as independent taxa. Sequence alignment and tree constructions were performed as described by Juhasz et al., 2018[37]. Visualisation and annotation of the phylogenetic tree was performed using CLC Genomics Workbench v21. Organism, protein group, and Pfam domain information was used for annotation.

CD-specific T cell epitopes[42] were mapped to the prolamin sequences using 100% sequence identity. Epitope count for each protein was summed and used to annotate the phylogenetic tree. Presence of baker's asthma epitopes was determined using epitopes retrieved from the Immune Epitope Database and Analysis Resource (https://www.iedb.org) and used for annotation.

Promoter analysis

Cis-acting transcription factor binding sites (TFBSs) associated with storage protein gene expression were collected from public promoter motif databases (PLACE[110], PlantCare[111]) and appended with motifs from published results[112–114]. The 1,000-bp 5′-end non-coding promoter

sequences of the identified gene models were extracted from the Sang oat genome, wheat genome (IWGSC assembly v1[7]), soybean (*Glycine max* assembly v2.1) and rice genome (*Oryza sativa* Japonica group assembly IRGSP-1.0). TFBS motifs were mapped to the promoter sequences and the mapped annotations were further analysed in FileMaker Pro (FileMaker Pro Advanced v17). The number of motifs was normalised by the gene size to evaluate motif enrichments. The MEME suite and Simple Enrichment Analysis (SEA[115]) was used to identify the significantly enriched motifs in the oat, wheat, rice and soybean prolamins and globulins. The total of normalised motif counts was calculated for each prolamin and globulin type in oat, wheat, rice and soybean and visualised using the Morpheus R package (Morpheus, https://software.broadinstitute.org/morpheus).

Protein extraction and LC-MS data acquisition

Plant material

Grains from the oat cv. Sang (identical seed batch used for genome sequencing) were visually inspected and milled using a Planetary Micro Mill PULVERISETTE.

Protein extraction

To extract protein from oats, three extraction solvents were used: "Urea" consisting of 8M urea in 0.1M Tris-HCl with 2% dithiothreitol (DTT) and pH adjusted to 8.5; "Tris-HCl" consisting of 50 mM Tris-HCl with 2% DTT; and "IPA-DTT" consisting of 55% (v/v) propan-2-ol (IPA) and 2% (w/v) DTT in water.

Wholemeal flour (20 mg; n=5 biological replicates) was weighed into a 1.5 mL micro-tube and 200 µL (10 µL/mg) of either Urea or Tris-HCl was added with vortex mixing until the flour was thoroughly mixed with the solvent. The tubes were then sonicated for 5 min at room temperature and incubated in a thermomixer (600 rpm, 45 min, 21°C). Next, the tubes were centrifuged for 15 min at 20,800 ×g. For the IPA-DTT extraction protocol, flour samples (20 mg) were resuspended in IPA-DTT with vortex mixing and sonication for 5 min. The sample tubes were incubated at 50°C for 30 min in a thermomixer (600 rpm). Finally, the sample tubes were centrifuged for 15 min at 20,800 ×g.

Protein digestion

Protein extracts (100 µL, n=5) were transferred to 10 kDa MWCO filters (Millipore, Sydney, Australia. Protein digestion protocol was previously described in detail by Bose et al., 2019[116]. In brief, the protein on the filter was washed twice with a buffer consisting of 8 M urea in 0.1 M Tris-HCl (pH 8.5) with centrifugation for 15 min at 20,800 × g. Iodoacetamide (50 mM; 100 µL) prepared in 8 M urea and 100 mM Tris-HCl was added to the filters for cysteine alkylation with incubation in the dark for 20 min prior to centrifugation for 10 min at 20,800 × g. The buffer was exchanged with 100 mM ammonium bicarbonate (pH 8.0) by two consecutive wash/centrifugation steps. The sequencing grade digestion enzyme, trypsin (Promega, Alexandria, Australia) solution (4 µg in 200 µL, 20 µg/mL in 50 mM ammonium bicarbonate and 1 mM $CaCl_2$) was applied to the filter and incubated for 18 h at 37°C in a thermomixer at 600 rpm. The tryptic peptides were collected by centrifugation (20,800 x g, 15 min) followed by an additional wash with 200 µL of 50 mM ammonium bicarbonate, and the

combined filtrates were subsequently lyophilized. Digested peptides were resuspended in 100 µL of 1% formic acid before analysis by LC-MS/MS as previously described by Bose et al. (2019)[116] .

Global proteome profiling

The digested peptides were reconstituted in 100 µL of 1% formic acid (FA) and chromatographic separation (4 µL) on an Ekspert nanoLC415 (Eksigent, Dublin, CA, U.S.A.) directly coupled to a TripleTOF 6600 liquid chromatography tandem mass spectrometry (LC-MS/MS, SCIEX, Redwood City, CA, USA). The peptides were desalted for 5 min on a ChromXP C18 (3 µm, 120 Å, 10 mm × 0.3 mm) trap column at a flow rate of 10 µL/min 0.1% FA, and separated on a ChromXP C18 (3 µm, 120 Å, 150 mm × 0.3 mm) column at a flow rate of 5 µL/min at 30˚C. A linear gradient from 3-25% solvent B over 38 min was employed followed by: 5 min from 25% B to 32% B; 2 min 32% B to 80% B; 3 min at 80% B; 1 min 80% B to 3% B; and 8 min re-equilibration. The solvents were as follows: (A) 5% dimethylsulfoxide (DMSO), 0.1% formic acid (FA), 94.9% water; and (B) 5% DMSO, 0.1% FA, 90% acetonitrile, 4.9% water. The instrument parameters were as follows: ion spray voltage 5,500 V, curtain gas 25 psi, GS1 15 psi, and GS2 15 psi, heated interface 150°C. Data were acquired in information-dependent acquisition mode comprising a time-of-flight (TOF)-MS survey scan followed by 30 MS/MS, each with a 40-ms accumulation time. First stage MS analysis was performed in positive ion mode with 0.25-s accumulation time. Tandem mass spectra were acquired on precursor ions >150 counts/s with charge state 2−5 and dynamic exclusion for 15 s with a 100 ppm mass tolerance. Spectra were acquired over two gas phase separation methods: *m/z* 350-650 and *m/z* 650–1,250 using the manufacturer's rolling collision energy based on the size and charge of the precursor ion. Protein identification was done using ProteinPilot™ 5.0.3 software (SCIEX) with searches conducted against the database of translated Sang gene models. The total number of proteins in the custom database was 159,362. The resulting mass spectrometry proteomics raw and result data have been deposited to the CSIRO public data access portal (*see Materials*). Peptides that were detected at the 95% confidence level were used in the phylogenetic tree to evidence protein level expression.

Synteny analysis of *TaZIP4-B2*

Synteny analysis was performed between *A. sativa* and *T. aestivum*[7] using the tool McScan[117] of the jcvi utility library[118].

Tblastn[75] was used to exploit the *A. sativa Sang* and OT3098 genome sequences for orthologs of *TaZIP4-B2* in order to exclude the possibility that *TaZIP4-B2* is missing in *A. sativa* due to missed annotation in *A. sativa* Sang or that it is missing in the assembly due to an assembly or sequencing error. However, no evidence for *TaZIP4-B2* besides the orthologs of the group 3 chromosomes was found in both the short-read (Sang) and long-read (OT3098) assemblies.
The orthogroup OG0011060, containing protein sequences of *TaZIP4-B2*, its paralogs, and the orthologs in *A. sativa, A. eriantha, A. atlantica, A. insularis, and A. longiglumis* as well as *B. distachyon* and *H. vulgare* as outgroups were extracted from the Orthofinder output (section "Orthofinder"). These, in addition to the orthologs from *A. sativa* cv. OT3098 were used for a

phylogenetic analysis. Protein sequences were aligned using MUSCLE[106] v3.8.1551 and a phylogenetic tree was calculated using fasttree[119] 2.1.11 and visualised with iTol[120] 6.3.

Gene expression analysis

Read trimming and quality control

RNA-seq reads from 62 samples and 6 different tissues (leaf, root, crown, seeds, glumes, and spikelets) were used for gene expression analysis and for network construction (Supplementary Table 19 datasets a,c,d,e,f,k,l). Reads were trimmed using Trimmomatic[67] [-phred33 LEADING:30 TRAILING:30 SLIDINGWINDOW:4:30 MINLEN:40] or fastp[121], v0.20.0, options --correction --adapter_sequence=AGATCGGAAGAGCACACGTCTGAACTCCAGTCA --adapter_sequence_r2=AGATCGGAAGAGCGTCGTGTAGGGAAAGAGTGT). FASTQC (v0.11.8) was used for quality control of the reads.

Mapping of RNA-seq reads to reference

Salmon v1.1.0[122] (index with option --keepDuplicates) was used to build a decoy-aware transcriptome index using the pseudomolecules as decoy sequences. Transcript abundances were quantified using Salmon (quant with options --libType A --gcBias --seqBias --validateMappings) for the trimmed RNA-seq reads mentioned above.

Differential gene expression analysis in a developmental time course of developing seeds

The transcript-level estimates from Salmon were summarised to the gene-level using the tximport package[123] v1.12.3. DESeq2[124] v1.24.0 was used to test for differentially expressed genes (DEGs) between the different stages of seed development (mid vs. early, late vs. early and late vs. mid) while controlling for differences between day and night samples (three biological replicates) (Supplementary Table 20, dataset "c"). A pre-filtering step was included to remove genes with less than 10 counts in total. A two-tailed threshold-based Wald test was used to test the null hypothesis that the absolute value of the logarithmic fold change (LFC) is less than or equal to 1. The Benjamini-Hochberg (BH)[125] procedure was used to adjust the *P* values for multiple testing. Genes with an adjusted *P* value of < 0.01 were considered significantly differentially expressed.

Analyses of expressed genes

Average TPM expression was calculated for each of the six tissues. Samples from different experiments and treatments were kept apart, resulting in 11 combinations of tissue/experiment/treatment hereafter called "condition". For average expression values across conditions, a gene was considered expressed when the TPM was above 0.5 in at least one condition. Using the average expression per condition, the expression of each gene across all conditions in which it was expressed (TPM ≥ 0.5), was determined. Thus, an average value across all conditions could be generated instead of a geometric mean across all samples, to account for the variation in the number of samples per condition. It also excludes conditions in which a gene is not expressed. This average across expressed conditions is referred to as the "global analysis" hereafter in the main text and in the supplementary materials and tables.

Definition of triads

For the identification of triads with a 1:1:1 correspondence across the three homoeologous subgenomes (exactly one member on each of the A-, C-, and D-subgenomes), the orthofinder clusters were pruned to exclude *A. thaliana* and filtered for clusters that include three A. *sativa genes,* 2 *A. insularis genes* and one of each *A. longiglumis* and *A. eriantha* as well as one *B. dystachion* gene as an outgroup. In addition, these clusters were required to be monophyletic for the *Avena* clades. From this, clusters with at least one *A. sativa* gene located on unanchored scaffolds ('chromosome unknown') were excluded. This resulted in 7,726 clusters with 1:1:1 A:C:D clusters. Next, we also identified a remaining set of 1,508 triads that were located in inter-subgenomic translocations and had an unbalanced copy number for the subgenomes. We adjusted subgenomic assignments of their genes using the translocation history in oat. The first set of triads is referred to as 'ancestral triads' while the second set is called 'relocated triads'.

Relative expression levels of the A-, C-, and D-subgenome homoeologs across triads

The analysis focused exclusively on the gene triads that had a 1:1:1 correspondence across the three homoeologous subgenomes, including 7,726 ancestral triads and 1,508 triads with at least one gene in a translocated region (total of 9,234 triads or 27,702 genes). A triad was considered to be expressed when the sum of the expression of the A-, C-, and D-subgenome homoeologs was $\geq$ 0.5 TPM. To standardise the relative expression of each homoeolog across the triad, the TPM of each of them was divided by the sum of all of them resulting in values between 0 and 1 for each of them. This normalised expression was calculated for the average across all samples ("global analysis" as described previously) as well as for seed tissue from early, middle and late seed developmental stages (Supplementary Table 19, dataset "c"). The values of the relative contributions of each subgenome per triad were used to plot the ternary diagrams using the R package ggtern[126].

Definition of homoeolog expression bias categories

Ideal normalised expression bias values were defined for the seven expression categories as described by Ramírez-González et al. (2018)[31]. For every triad, the euclidean distance was calculated between the observed normalised relative expression of the triad and the ideal normalised expression of each of the seven categories. The expression bias category for the triad was then assigned by selecting the expression category with the shortest distance. This was done for the average across all expressed tissues as well as for seed tissue only.

WGCNA network construction

Coexpression networks were built using the RNA-Seq datasets a, c, d, e, f, k, and l (Supplementary Table 19) as described above with the R-package WGCNA[127]. The previously defined transcript-level estimates from Salmon were summarised to the gene-level using the R-package tximport v1.12.3[123]. The expression level of each gene was normalised using the function varianceStabilizingTransformation from DESeq2[124] to eliminate differences in sequencing depth between studies. A threshold of 5 TPM in at least three samples was used an expression threshold and the most variable 20,000 gene (6,887 from the A-subgenome; 5,470 from the C-subgenome; 6,637 from the D-subgenome; 1,006 mapping to the unassigned chromosome) were selected as an input for the network. The plateau of the scale-free topology

fit index curve was used to set the soft thresholding for co-expression similarity. The selected soft powers used was 9. Dissimilarity was calculated as 1-adjacency. To identify co-expression modules, genes were clustered based on the dissimilarity measure. To detect gene co-expression modules the dynamic branch cut method cutreeDynamic was applied with the method "hybrid", a deepSplit of 1, and a minClusterSize of 30.

Orthology-based ontology annotation of oat genes

Inferred orthologous relationships were used to transfer automatic and experimentally validated annotations from orthologous genes to the respective genes in the genomes of *A sativa* cv. Sang. Gene Ontology (GO[128], Plant Ontology (PO[129], and Plant Trait Ontology (TO[130]; term annotations were obtained and pooled from Gene Ontology (http://geneontology.org/-gene-associations), TAIR (https://www.ara-bidopsis.org/), and Gramene (ftp://ftp.gramene.org/pub/-gramene/release52 /data/ontology/) resources. Gene identifiers were mapped to public resources using the UniProtKB mapping table (ftp://ftp.uniprot.org/pub/databases/ uniprot/current_release/knowledgebase/idmapping/). The pfam2GO mapping table available from the Gene Ontology resource (http://geneontology.org/external2go/pfam2go) was employed to transfer GO terms based on the inferred domain architectures. The source evidence classes of the annotated, orthologous genes were translated into target evidence codes of *A. sativa* genes using automatic annotations/ IEA (Inferred by Electronic Annotation), experimental and reviewed computational analyses (http://www.geneontology.org/page/guide-go-evidence-codes), and pfam2GO/ ISM (Inferred from Sequence Model).

Single-gene-mapping of the *glossy.1* epicuticular wax mutant

Plant material, mutants and F_3 segregating pools

The glossy panicle mutant designated *glossy.1* was identified among the Belinda TILLING population[45] during a field amplification experiment in 2017, and a forward genetics investigation of the genetic cause was initiated. This mutant exhibited a glossy panicle and sheath compared to the glaucous phenotype of the parental cv. Belinda, cv. Sang and other members of the TILLING population. In contrast to the glossy panicle and sheath, the leaf blades were similarly glaucous. The *glossy.1* phenotype was stable under greenhouse conditions with an 18-h photoperiod. A cross was made using Belinda as mother and the mutant as pollen donor (Belinda × *glossy.1*). The resulting F_1 seeds were germinated in soil in a greenhouse and allowed to self-fertilise. Upon ocular inspection, the Belinda × *glossy.1* mutant F_1 plants appeared less glaucous than Belinda, indicative of the mutation being semi-dominant. An F_2 population was raised to maturity in the greenhouse and the allelic status of the *glossy.1* locus was deduced from the phenotype of F_3 progeny (40 F_3 siblings per F_2 plant). The number of glossy and glaucous F_2 progeny was 11 and 45 (14 homozygous, 31 heterozygous) respectively, which was compatible with a single *glossy.1* locus being responsible for the glossy phenotype (null hypothesis is that homozygous glossy:heterozygous:homozygous glaucous have distribution 1:2:1, $\chi^2(2, N = 56) = 0.96$, $P = 0.62$). A single seed from each homozygous glossy and homozygous glaucous progeny was sown in vermiculite and the second leaf was collected and pooled (11 and 14 plants, respectively) to form two tissue pools for DNA isolation, referred to hereafter as the glossy and glaucous pools. The basic principle

of bulk segregant analysis was used in an integrative genomics approach to conduct genetic mapping of shared alleles in back-cross progeny pools.

During the early grain filling stage, pairs of whole glumes were collected from four representative F_3 glossy and glaucous sibling plants, and this tissue was processed as outlined in the following *sections* on RNA isolation, SEM, and GC/MS.

Eventual identification of the candidate *glossy.1* locus using the forward genetics approach is outlined hereafter, in combination with an intersection of moderate or high impact mutations in other whole genome sequenced TILLING mutants, allowed the identification of one additional mutant (termed *glossy.2*) with a nonsense mutation in the same candidate gene. A reverse genetics approach on this second mutant included SEM of the glume to investigate the phenotype of *glossy.2* at the cuticle surface.

Scanning electron microscopy (SEM)

For SEM, whole glumes, the apical portion of the sheath and basal portion of the flag leaf from representative F_3 glossy and glaucous sibling plants were air dried in clean petri dishes at 50°C in darkness for 48-hr. Air dried specimens were carefully glued onto SEM stubs with the glume adaxial surface (side facing the seed), flag leaf abaxial surface, and sheath adaxial surface in contact with the stub and sputter-coated with gold (Cesington 108 auto, 45 seconds, 20mA). For the *glossy.1* and *glossy.2* mutants, Belinda and Sang, only the glume was analysed. The preparations were viewed using a scanning electron microscope (Hitachi SU3500) applying an accelerating voltage of 5 kV. Images were collected near the centre of the glume for all specimens at ×1000 and ×4000 magnification.

Wax extraction and Gas chromatography-mass spectrometry (GC/MS)

For representative F_3 glossy and glaucous sibling plants, the glumes from 10 florets from a single panicle and plant at the early grain filling stage served as a single GC/MS sample and biological replicate. The accompanying flag leaf and the apical 15 cm of sheath (with culm removed) served as single biological replicates. Two replicates were used for glume, three replicates were used for sheath and the glaucous flag leaf, and four replicates were used for the glossy flag leaf. For flag leaf and sheath samples, wax was extracted by immersing the tissue for 10 s in a glass tube containing 10 mL dichloromethane. For glumes, wax was extracted in the same way, but with the glumes within a sample immersed consecutively in the dichloromethane. For glume single samples, wax was extracted by consecutively immersing the tissue for 10 s in a glass tube containing 10 mL dichloromethane. The solvent was evaporated under a stream of nitrogen gas at room temperature. The solid residue was redissolved in 30 µL dichloromethane and transferred to glass vials. Then, 30 µL of trimethylsilyl trifluoroacetamide (MSTFA) was added and the vial left at room temperature for 30 min. Analyses were performed on an Agilent 7890 gas chromatograph connected to a 5975 single quadrupole mass analyzer according to a previously published method[131], but with the final isocratic temperature adjusted to 340 C. Data were analysed using a Matlab script [132] and peaks annotated by retention index and mass spectra matching in the NIST MS library. Metabolites were identified based on retention index and mass spectrum in the NIST MS library.

GC/MS statistics

The two genotypes and three tissue types were compared using Welch's t-test for sheath and flag leaf respectively. P-values were adjusted for multiple testing using the Benjamini-Hochberg FDR method. As only two replicates were used for glume, no statistics were computed for this tissue type.

Illumina whole-genome resequencing of cultivars, EMS mutants, and back-crossed pools.

The glossy and glaucous tissue pools were ground to a fine powder using liquid nitrogen in a mortar and pestle. Genomic DNA was isolated using the Qiagen DNeasy Plant Mini Kit according to the manufacturer's protocol. Shotgun libraries were prepared using 350-bp fragments (chemical fragmentation) and the Illumina TruSeq PCR-free Preparation Kit (Illumina, San Diego, CA). Libraries were sequenced on a NovaSeq6000 (NovaSeq Control Software 1.7.0/RTA v3.4.4) with a 151nt(Read1)-10nt(Index1)-10nt(Index2)-151nt(Read2) setup using 'NovaSeqXp' workflow in 'S4' mode flowcell. The Bcl to FastQ conversion was performed using bcl2fastq_v2.20.0.422 from the CASAVA software suite. The quality scale used was Sanger / phred33 / Illumina 1.8+. The cultivars Sang and Belinda were similarly sequenced to identify mutations novel to the TILLING mutants. Additional TILLING mutants with novel phenotypes were also sequenced outside the main aims of this work, and data from one such mutant (*glossy.2*) were used in the forward genetics approach for *glossy.1*. For data availability see Supplementary Table 19.

Glume RNA sequencing

A pair of glumes from a single floret and individual plant served as a single biological replicate. Tissue was collected for four replicates each from representative F_3 glossy and glaucous sibling plants. Each sample was snap frozen in liquid nitrogen in 2mL screw cap tubes containing two 6mm glass beads. While frozen, tissue was pulverised using a Precellys Tissue Homogenizer and this sample tube then served as the starting vessel for total RNA isolation using the Qiagen RNeasy plant Mini kit following the manufacturer's instructions. Sequencing libraries were prepared as outlined above except that libraries were sequenced on NovaSeq6000 (NovaSeq Control Software 1.7.0/RTA v3.4.4) with a 151nt(Read1)-10nt(Index1)-10nt(Index2)-151nt(Read2) setup using 'NovaSeqXp' workflow in 'S4' mode flow cell. For data availability see Supplementary Table 19.

Variant calling and analysis

Read trimming was done using fastp[121] v0.20.1 as described above. FASTQC[133] v0.11.9 was used for quality control of the trimmed reads. BWA-MEM2 v2.2.1 was used to map reads to the reference genome, with SAMtools[134] v1.12 used to produce the required BAM file. MultiQC[135] v1.10.1 was used for synthesis of the quality control report. DeepVariant[136] v1.1.0; GNU Parallel[137] v20210422, and GLNexus[138,139] v1.3.1 were used to call variants. The full workflow is available as nikostr/dna-seq-deepvariant-glnexus-variant-calling[140] (v0.3.1) and was run using Snakemake[141] v6.5.1.

Variants present in unrelated lines from the TILLING and parent population were filtered using bcftools v1.12 [134]. To identify regions conserved in the pool, a sliding mean of the mutant allele

frequency was plotted across each chromosome using custom R[64] scripts (v4.1.0; tidyverse[95] v1.3.1; ggplot2[142] v3.3.5; vcfR[143] v1.12.0; svglite[144] v2.0.0; fs[145] v1.5.0; slider[146] v0.2.2; tidymodels[147] v0.1.4 window size of 100 observations). Bootstrap confidence intervals were computed by recomputing the sliding mean across 1,000 bootstrap samples for each chromosome, and calculating the interval capturing 95% of the observations for each position across the genome.

SnpEff[148] v4.3.1t was used to identify mutated genes, and genes with moderate- or high-impact mutations were considered for further analysis. Only homozygous mutations were considered as a part of this analysis. Genes were additionally filtered based on being located in a region identified to be conserved in the pool, gene expression in the glume, read support, type of the mutation found in the gene, and whether or not the gene was mutated in other lines sharing the phenotype. Genes on chrUn were included in the analysis, and interesting candidates were localised using the Hi-C data previously used to construct the pseudomolecules.

Glume gene expression analysis

The RNA-seq reads from the glume tissue samples were trimmed using fastp[121] v0.20.0 and gene-level estimates were obtained using Salmon[122] v1.1.0 and tximport[123] v1.12.3 as described above. DESeq2[124] v1.24.0 was used to detect DEGs between the glossy and glaucous genotypes (4 biological replicates) (Supplementary Table 20, dataset "f"). We used a two-tailed Wald test to test the null hypothesis of zero LFC (BH adjusted P value < 0.01).

Beta-diketone biosynthetic gene cluster phylogenetic analysis

Cluster genes were selected based either on proximity to our candidate gene, or on being a part of the same orthogroup as either of the candidate, the barley *cer-q*, *-u*, and *-c* genes or of a gene close to our candidate. Genes in OT3098 were selected based on the source of the projection annotation gene model, and coordinates were updated to match OT3098 v2 using gmap[84] v2020.06.01.

The genes were aligned using MUSCLE[106] v3.8.155, and phylogenetic trees were built using fasttree[119] v2.1.10. Alignments were visualised using a custom R script (v4.1.0, ggplot2 v3.3.5, tidyverse v1.3.1).

Trees were also visualised using R (v4.1.0; treeio[149] v1.16.1; ggtree[150] v3.0.1, as were the clusters (R v4.1.0; patchwork[151] v1.1.1; gggenes[108] v0.4.1; ggplot2 v3.3.5; tidyverse v1.3.1).

References

55. Avni, R. *et al.* Wild emmer genome architecture and diversity elucidate wheat evolution and domestication. *Science* **357**, 93–97 (2017).
56. Jiao, Y. *et al.* Improved maize reference genome with single-molecule technologies. *Nature* **546**, 524–527 (2017).
57. Paterson, A. H. *et al.* The Sorghum bicolor genome and the diversification of grasses. *Nature* **457**, 551–556 (2009).

58. Luo, M.-C. *et al.* Genome sequence of the progenitor of the wheat D genome Aegilops tauschii. *Nature* **551**, 498–502 (2017).
59. Padmarasu, S., Himmelbach, A., Mascher, M. & Stein, N. In Situ Hi-C for Plants: An Improved Method to Detect Long-Range Chromatin Interactions. *Methods Mol. Biol.* **1933**, 441–472 (2019).
60. Rice, P., Longden, I. & Bleasby, A. EMBOSS: the European Molecular Biology Open Software Suite. *Trends Genet.* **16**, 276–277 (2000).
61. Li, H. Minimap2: pairwise alignment for nucleotide sequences. *Bioinformatics* **34**, 3094–3100 (2018).
62. Li, H. *et al.* The Sequence Alignment/Map format and SAMtools. *Bioinformatics* **25**, 2078–2079 (2009).
63. Quinlan, A. R. & Hall, I. M. BEDTools: a flexible suite of utilities for comparing genomic features. *Bioinformatics* **26**, 841–842 (2010).
64. R Core Team. R: A Language and Environment for Statistical Computing. (2019).
65. Beier, S. *et al.* Construction of a map-based reference genome sequence for barley, Hordeum vulgare L. *Sci Data* **4**, 170044 (2017).
66. Vaillancourt, B. & Robin Buell, C. High molecular weight DNA isolation method from diverse plant species for use with Oxford Nanopore sequencing. *bioRxiv* 783159 (2019) doi:10.1101/783159.
67. Bolger, A. M., Lohse, M. & Usadel, B. Trimmomatic: a flexible trimmer for Illumina sequence data. *Bioinformatics* **30**, 2114–2120 (2014).
68. Koren, S. *et al.* Canu: scalable and accurate long-read assembly via adaptive k-mer weighting and repeat separation. *Genome Res.* **27**, 722–736 (2017).
69. Walker, B. J. *et al.* Pilon: an integrated tool for comprehensive microbial variant detection and genome assembly improvement. *PLoS One* **9**, e112963 (2014).
70. Paten, B. *et al.* Cactus: Algorithms for genome multiple sequence alignment. *Genome Res.* **21**, 1512–1528 (2011).
71. Kurtz, S., Narechania, A., Stein, J. C. & Ware, D. A new method to compute K-mer frequencies and its application to annotate large repetitive plant genomes. *BMC Genomics* **9**, 517 (2008).
72. Gremme, G., Brendel, V., Sparks, M. E. & Kurtz, S. Engineering a software tool for gene structure prediction in higher organisms. *Information and Software Technology* **47**, 965–978 (2005).
73. Kim, D., Paggi, J. M., Park, C., Bennett, C. & Salzberg, S. L. Graph-based genome alignment and genotyping with HISAT2 and HISAT-genotype. *Nat. Biotechnol.* **37**, 907–915 (2019).
74. Pertea, M. *et al.* StringTie enables improved reconstruction of a transcriptome from RNA-seq reads. *Nat. Biotechnol.* **33**, 290–295 (2015).
75. Camacho, C. *et al.* BLAST+: architecture and applications. *BMC Bioinformatics* **10**, 421 (2009).
76. Finn, R. D., Clements, J. & Eddy, S. R. HMMER web server: interactive sequence similarity searching. *Nucleic Acids Res.* **39**, W29–37 (2011).
77. Pertea, G. & Pertea, M. GFF Utilities: GffRead and GffCompare. *F1000Res.* **9**, 304 (2020).
78. Cock, P. J. A. *et al.* Biopython: freely available Python tools for computational molecular biology and bioinformatics. *Bioinformatics* **25**, 1422–1423 (2009).
79. Buchfink, B., Xie, C. & Huson, D. H. Fast and sensitive protein alignment using DIAMOND. *Nat. Methods* **12**, 59–60 (2015).
80. Wicker, T., Matthews, D. E. & Keller, B. TREP: a database for Triticeae repetitive elements. *Trends Plant*

Sci. **7**, 561–562 (2002).

81. Grabherr, M. G. *et al.* Full-length transcriptome assembly from RNA-Seq data without a reference genome. *Nat. Biotechnol.* **29**, 644–652 (2011).
82. Dobin, A. *et al.* STAR: ultrafast universal RNA-seq aligner. *Bioinformatics* **29**, 15–21 (2013).
83. Haas, B. J. *et al.* Improving the Arabidopsis genome annotation using maximal transcript alignment assemblies. *Nucleic Acids Res.* **31**, 5654–5666 (2003).
84. Wu, T. D., Reeder, J., Lawrence, M., Becker, G. & Brauer, M. J. GMAP and GSNAP for Genomic Sequence Alignment: Enhancements to Speed, Accuracy, and Functionality. *Methods Mol. Biol.* **1418**, 283–334 (2016).
85. Mistry, J., Finn, R. D., Eddy, S. R., Bateman, A. & Punta, M. Challenges in homology search: HMMER3 and convergent evolution of coiled-coil regions. *Nucleic Acids Res.* **41**, e121 (2013).
86. Suzek, B. E. *et al.* UniRef clusters: a comprehensive and scalable alternative for improving sequence similarity searches. *Bioinformatics* **31**, 926–932 (2015).
87. Mistry, J. *et al.* Pfam: The protein families database in 2021. *Nucleic Acids Res.* **49**, D412–D419 (2021).
88. Hoff, K. J. & Stanke, M. Predicting Genes in Single Genomes with AUGUSTUS. *Curr. Protoc. Bioinformatics* **65**, e57 (2019).
89. Haas, B. J. *et al.* Automated eukaryotic gene structure annotation using EVidenceModeler and the Program to Assemble Spliced Alignments. *Genome Biol.* **9**, R7 (2008).
90. Seppey, M., Manni, M. & Zdobnov, E. M. BUSCO: Assessing Genome Assembly and Annotation Completeness. *Methods Mol. Biol.* **1962**, 227–245 (2019).
91. Tomato Genome Consortium. The tomato genome sequence provides insights into fleshy fruit evolution. *Nature* **485**, 635–641 (2012).
92. Shumate, A. & Salzberg, S. L. Liftoff: an accurate gene annotation mapping tool. *bioRxiv* 2020.06.24.169680 (2020) doi:10.1101/2020.06.24.169680.
93. Benson, G. Tandem repeats finder: a program to analyze DNA sequences. *Nucleic Acids Res.* **27**, 573–580 (1999).
94. Spannagl, M. *et al.* PGSB PlantsDB: updates to the database framework for comparative plant genome research. *Nucleic Acids Res.* **44**, D1141–7 (2016).
95. Wickham, H. *et al.* Welcome to the tidyverse. *J. Open Source Softw.* **4**, 1686 (2019).
96. Tinker, N. A., Bekele, W. A. & Hattori, J. Haplotag: Software for Haplotype-Based Genotyping-by-Sequencing Analysis. *G3* **6**, 857–863 (2016).
97. Bradbury, P. J. *et al.* TASSEL: software for association mapping of complex traits in diverse samples. *Bioinformatics* **23**, 2633–2635 (2007).
98. Kokot, M., Dlugosz, M. & Deorowicz, S. KMC 3: counting and manipulating k-mer statistics. *Bioinformatics* **33**, 2759–2761 (2017).
99. Kurtz, S. The Vmatch large scale sequence analysis software-A Manual. (2011).
100. Emms, D. M. & Kelly, S. OrthoFinder: phylogenetic orthology inference for comparative genomics. *Genome Biol.* **20**, 238 (2019).
101. Virtanen, P. *et al.* SciPy 1.0: fundamental algorithms for scientific computing in Python. *Nat. Methods* **17**, 261–272 (2020).

102. Yin, Y., Huang, J. & Xu, Y. The cellulose synthase superfamily in fully sequenced plants and algae. *BMC Plant Biol.* **9**, 99 (2009).
103. Little, A. *et al.* Revised Phylogeny of the Cellulose Synthase Gene Superfamily: Insights into Cell Wall Evolution. *Plant Physiol.* **177**, 1124–1141 (2018).
104. Kaur, S., Dhugga, K. S., Beech, R. & Singh, J. Genome-wide analysis of the cellulose synthase-like (Csl) gene family in bread wheat (Triticum aestivum L.). *BMC Plant Biol.* **17**, 193 (2017).
105. Jones, P. *et al.* InterProScan 5: genome-scale protein function classification. *Bioinformatics* **30**, 1236–1240 (2014).
106. Edgar, R. C. MUSCLE: multiple sequence alignment with high accuracy and high throughput. *Nucleic Acids Res.* **32**, 1792–1797 (2004).
107. Tanner, G. *et al.* Preparation and Characterization of Avenin-Enriched Oat Protein by Chill Precipitation for Feeding Trials in Celiac Disease. *Front Nutr* **6**, 162 (2019).
108. Wilkins, D. gggenes: Draw Gene Arrow Maps in 'ggplot2'. (2020).
109. Kassambara, A. ggpubr: 'ggplot2' Based Publication Ready Plots. (2020).
110. Higo, K., Ugawa, Y., Iwamoto, M. & Korenaga, T. Plant cis-acting regulatory DNA elements (PLACE) database: 1999. *Nucleic Acids Res.* **27**, 297–300 (1999).
111. Lescot, M. *et al.* PlantCARE, a database of plant cis-acting regulatory elements and a portal to tools for in silico analysis of promoter sequences. *Nucleic Acids Res.* **30**, 325–327 (2002).
112. Juhász, A., Makai, S., Sebestyén, E., Tamás, L. & Balázs, E. Role of conserved non-coding regulatory elements in LMW glutenin gene expression. *PLoS One* **6**, e29501 (2011).
113. Liu, X. *et al.* Two short sequences in OsNAR2.1 promoter are necessary for fully activating the nitrate induced gene expression in rice roots. *Sci. Rep.* **5**, 11950 (2015).
114. Chardin, C., Girin, T., Roudier, F., Meyer, C. & Krapp, A. The plant RWP-RK transcription factors: key regulators of nitrogen responses and of gametophyte development. *J. Exp. Bot.* **65**, 5577–5587 (2014).
115. Bailey, T. L. & Grant, C. E. SEA: Simple Enrichment Analysis of motifs. *bioRxiv* 2021.08.23.457422 (2021) doi:10.1101/2021.08.23.457422.
116. Bose, U. *et al.* Optimisation of protein extraction for in-depth profiling of the cereal grain proteome. *J. Proteomics* **197**, 23–33 (2019).
117. Tang, H. *et al.* Synteny and collinearity in plant genomes. *Science* **320**, 486–488 (2008).
118. Tang, H., Krishnakumar, V. & Li, J. *jcvi: JCVI utility libraries*. (2015). doi:10.5281/zenodo.31631.
119. Price, M. N., Dehal, P. S. & Arkin, A. P. FastTree 2--approximately maximum-likelihood trees for large alignments. *PLoS One* **5**, e9490 (2010).
120. Letunic, I. & Bork, P. Interactive Tree Of Life (iTOL) v5: an online tool for phylogenetic tree display and annotation. *Nucleic Acids Res.* **49**, W293–W296 (2021).
121. Chen, S., Zhou, Y., Chen, Y. & Gu, J. fastp: an ultra-fast all-in-one FASTQ preprocessor. *Bioinformatics* **34**, i884–i890 (2018).
122. Patro, R., Duggal, G., Love, M. I., Irizarry, R. A. & Kingsford, C. Salmon provides fast and bias-aware quantification of transcript expression. *Nat. Methods* **14**, 417–419 (2017).
123. Soneson, C., Love, M. I. & Robinson, M. D. Differential analyses for RNA-seq: transcript-level estimates improve gene-level inferences. *F1000Res.* **4**, 1521 (2015).

124. Love, M. I., Huber, W. & Anders, S. Moderated estimation of fold change and dispersion for RNA-seq data with DESeq2. *Genome Biol.* **15**, 550 (2014).
125. Benjamini, Y. & Hochberg, Y. Controlling the False Discovery Rate: A Practical and Powerful Approach to Multiple Testing. *Journal of the Royal Statistical Society: Series B (Methodological)* vol. 57 289–300 (1995).
126. Hamilton, N. E. & Ferry, M. ggtern: Ternary Diagrams Using ggplot2. *Journal of Statistical Software, Code Snippets* **87**, 1–17 (2018).
127. Langfelder, P. & Horvath, S. WGCNA: an R package for weighted correlation network analysis. *BMC Bioinformatics* **9**, 559 (2008).
128. The Gene Ontology Consortium. Expansion of the Gene Ontology knowledgebase and resources. *Nucleic Acids Res.* **45**, D331–D338 (2017).
129. Cooper, L. *et al.* The plant ontology as a tool for comparative plant anatomy and genomic analyses. *Plant Cell Physiol.* **54**, e1 (2013).
130. Arnaud, E. *et al.* Towards a reference plant trait ontology for modeling knowledge of plant traits and phenotypes. in (Unpublished, 2012). doi:10.13140/2.1.2550.3525.
131. Danielsson, A. P. H., Moritz, T., Mulder, H. & Spégel, P. Development and optimization of a metabolomic method for analysis of adherent cell cultures. *Anal. Biochem.* **404**, 30–39 (2010).
132. Jonsson, P. *et al.* High-throughput data analysis for detecting and identifying differences between samples in GC/MS-based metabolomic analyses. *Anal. Chem.* **77**, 5635–5642 (2005).
133. Andrews, S. Babraham bioinformatics-FastQC a quality control tool for high throughput sequence data. *URL: https://www. bioinformatics. babraham. ac. uk/projects/fastqc* (2010).
134. Danecek, P. *et al.* Twelve years of SAMtools and BCFtools. *Gigascience* **10**, (2021).
135. Ewels, P., Magnusson, M., Lundin, S. & Käller, M. MultiQC: summarize analysis results for multiple tools and samples in a single report. *Bioinformatics* **32**, 3047–3048 (2016).
136. Poplin, R. *et al.* A universal SNP and small-indel variant caller using deep neural networks. *Nat. Biotechnol.* **36**, 983–987 (2018).
137. Tange, O. *GNU Parallel 20210422 ('Ever Given')*. (2021). doi:10.5281/zenodo.4710607.
138. Lin, M. F. *et al.* GLnexus: joint variant calling for large cohort sequencing. *bioRxiv* 343970 (2018) doi:10.1101/343970.
139. Yun, T. *et al.* Accurate, scalable cohort variant calls using DeepVariant and GLnexus. *Bioinformatics* (2021) doi:10.1093/bioinformatics/btaa1081.
140. nikostr. *nikostr/dna-seq-deepvariant-glnexus-variant-calling: v0.3.1*. (2021). doi:10.5281/zenodo.5045703.
141. Mölder, F. *et al.* Sustainable data analysis with Snakemake. *F1000Res.* **10**, 33 (2021).
142. Wickham, H. ggplot2: Elegant Graphics for Data Analysis. (2016).
143. Knaus, B. J. & Grünwald, N. J. vcfr: a package to manipulate and visualize variant call format data in R. *Mol. Ecol. Resour.* **17**, 44–53 (2017).
144. Wickham, H. *et al.* svglite: An 'SVG' Graphics Device. (2021).
145. Hester, J. & Wickham, H. fs: Cross-Platform File System Operations Based on 'libuv'. (2020).
146. Vaughan, D. slider: Sliding Window Functions. (2021).
147. Kuhn, M. & Wickham, H. Tidymodels: a collection of packages for modeling and machine learning using

tidyverse principles. (2020).
148. Cingolani, P. *et al.* A program for annotating and predicting the effects of single nucleotide polymorphisms, SnpEff: SNPs in the genome of Drosophila melanogaster strain w1118; iso-2; iso-3. *Fly* **6**, 80–92 (2012).
149. Wang, L.-G. *et al.* Treeio: An R Package for Phylogenetic Tree Input and Output with Richly Annotated and Associated Data. *Mol. Biol. Evol.* **37**, 599–603 (2020).
150. Yu, G. Using ggtree to Visualize Data on Tree-Like Structures. *Curr. Protoc. Bioinformatics* **69**, e96 (2020).
151. Pedersen, T. L. patchwork: The Composer of Plots. (2020).
