# Supplementary material for: The mosaic oat genome gives insights into a uniquely healthy cereal crop
Source: Nature. 2022 May 18;606(7912):113–9. doi: 10.1038/s41586-022-04732-y (PMC9159951; doi:10.1038/s41586-022-04732-y)
Supplement: Supplementary file 2 — Reporting Summary [file 41586_2022_4732_MOESM2_ESM.pdf]

nature portfolio

Corresponding author(s): Nick Sirijovski and Manuel Spannagl
2021-07-11934E

Last updated by author(s): Mar 11, 2022

Reporting Summary

Nature Portfolio wishes to improve the reproducibility of the work that we publish. This form provides structure for consistency and transparency in reporting. For further information on Nature Portfolio policies, see our Editorial Policies and the Editorial Policy Checklist.

Statistics

For all statistical analyses, confirm that the following items are present in the figure legend, table legend, main text, or Methods section.

n/a	Confirmed	
☐	☒	The exact sample size (*n*) for each experimental group/condition, given as a discrete number and unit of measurement
☐	☒	A statement on whether measurements were taken from distinct samples or whether the same sample was measured repeatedly
☐	☒	The statistical test(s) used AND whether they are one- or two-sided<br>*Only common tests should be described solely by name; describe more complex techniques in the Methods section.*
☐	☒	A description of all covariates tested
☐	☒	A description of any assumptions or corrections, such as tests of normality and adjustment for multiple comparisons
☐	☒	A full description of the statistical parameters including central tendency (e.g. means) or other basic estimates (e.g. regression coefficient) AND variation (e.g. standard deviation) or associated estimates of uncertainty (e.g. confidence intervals)
☐	☒	For null hypothesis testing, the test statistic (e.g. *F*, *t*, *r*) with confidence intervals, effect sizes, degrees of freedom and *P* value noted<br>*Give P values as exact values whenever suitable.*
☒	☐	For Bayesian analysis, information on the choice of priors and Markov chain Monte Carlo settings
☒	☐	For hierarchical and complex designs, identification of the appropriate level for tests and full reporting of outcomes
☒	☐	Estimates of effect sizes (e.g. Cohen's *d*, Pearson's *r*), indicating how they were calculated

Our web collection on statistics for biologists contains articles on many of the points above.

Software and code

Policy information about availability of computer code

Data collection	Custom code developed in this study can be found in the GitHub node at https://github.com/PGSB-HMGU/oatkmers.<br>Code developed in other studies (but used here) can be found at https://github.com/PGSB-HMGU/plant.annot.<br>Software for data collection included HiSeq Control Software HD 3.4.0.38/RTA 2.7.7, NovaSeq Control Software 1.7.0/RTA v3.4.4, and bcl2fastq_v2.20.0.422.
Data analysis	A myriad software was used in this study, all of which have been listed and cited. These include DeNovoMAGIC v3.0, TRITEX pipeline, EMBOSS restrict, Minimap2, SAMtools, Novosort (http://www.novocraft.com/products/novosort/), BEDTools, FASTQC v0.11.8, Canu v1.9, Arrow from the GenomicConsensus package in the Pacific BioSciences SMRT portal v5.1.0, PILON v0.22, BBDuk (https://jgi.doe.gov/data-and-tools/bbtools/bb-tools-user-guide/), cactus pipeline v1.0, tallymer subtools from the genome tools package v1.6.1, GenomeThreader, v1.7.1, HISAT2 v2.1.0, Stringtie v1.2.3, Transdecoder v3.0.0, BLAST+, gffread v0.11.6, Biopython, Diamond v0.9.25 and v0.9.29.130, STAR, v2.7.6a, Trinity v2.8.5, PASApipeline v2.3.3 and v2.4.1, GMAP v2017.11.15 and v2020.06.01, TransDecoder v5.5.0, HMMER3 v3.1b2 and v3.2.1, Augustus v3.3.1 and v3.3.3, EvidenceModeler v73350ce, BUSCO v3.0.2 liliopsida_odb10 created on 2017-12-01 and embryophyta_odb9 created on 2017-02-13 (protein mode), BUSCO v4.0.4 liliopsida_odb10 created on 2019-11-20 (protein mode), AHRD v3.3.3, TandemRepeatsFinder v4.07b, vmatch dbcluster, R, Haplotag, FSHap as implemented in TASSEL v5.0, KMC tools v3, Louvain algorithm (https://github.com/taynaud/python-louvain), Orthofinder v2.4, SciPy v1.6.1, networkx v2.5, InterProScan5, CLC Genomics Workbench v21, ggpubr, FileMaker Pro Advanced v17, MEME suit, Morpheus R package, ProteinPilot™ 5.0.3 software (SCIEX), McScan of the jcvi utility library (https://github.com/tanghaibao/jcvi), MUSCLE v3.8.155, fasttree v2.1.10 and v2.1.11, iTol v6.3, Trimmomatic, fastp v0.20.0, Salmon v1.1.0, tximport package v1.12.3, DESeq2 v1.24.0, ggtern, WGCNA, tidyverse v1.3.1, ggplot2 v3.3.5, vcfR v1.12.0, svglite v2.0.0, fs v1.5.0, slider v0.2.2, tidymodels v0.1.4, treeio v1.16.1, ggtree v3.0.1, patchwork v1.1.1, gggenes v0.4.1, BWA-MEM2 v2.2.1, MultiQC v1.10.1, DeepVariant v1.1.0, GNU Parallel v20210422, GLNexus v1.3.1, Snakemake v6.5.1, bcftools v1.12, McScanX, Matlab, and SnpEff v4.3.1t.

For manuscripts utilizing custom algorithms or software that are central to the research but not yet described in published literature, software must be made available to editors and reviewers. We strongly encourage code deposition in a community repository (e.g. GitHub). See the Nature Portfolio guidelines for submitting code & software for further information.

Data

Policy information about availability of data

All manuscripts must include a data availability statement. This statement should provide the following information, where applicable:

- Accession codes, unique identifiers, or web links for publicly available datasets
- A description of any restrictions on data availability
- For clinical datasets or third party data, please ensure that the statement adheres to our policy

The raw sequence data used for de novo whole genome assembly are available from the European Nucleotide Archive (ENA) under accession number PRJEB44810 (A. sativa cv. Sang) and from the Sequence Read Archive (SRA) under accession number PRJNA727490 (A. insularis BYU209) and PRJNA726919 (A. longiglumis CN58138). Chromosome conformation capture (Hi-C) sequencing data are available from ENA under accession PRJEB43668 (A. sativa cv. Sang), PRJEB43670 (A. insularis BYU209) and PRJEB43669 (A. longiglumis CN58138). Chromosome-scale sequence assemblies (pseudomolecules) are available from ENA under accession PRJEB44810 (A. sativa cv. Sang), PRJEB45088 (A. insularis BYU209) and PRJEB45087 (A. longiglumis CN58138). The raw RNA-seq and WGS data generated in this study are available under ENA accession number PRJEB46365. Pseudomolecules, annotation data and analysis results are available in the Plant Genomics & Phenomics (PGP) Research Data Repository at http://dx.doi.org/10.5447/ipk/2022/2. The DOI was registered using e!DAL (https://edal.ipk-gatersleben.de/). Pseudomolecules, annotation data and associated analyses for A. sativa cv. Sang, A. longiglumis, and A. insularis are also available from GrainGenes60: Sang genome browser: https://wheat.pw.usda.gov/jb/?data=/ggds/oat-sang; Sang data download: https://wheat.pw.usda.gov/GG3/content/avena-sang-download; A. longiglumis genome browser: https://wheat.pw.usda.gov/jb/?data=/ggds/oat-longiglumis; A. longiglumis data download: https://wheat.pw.usda.gov/GG3/content/avena-longiglumis-download; A. insularis genome browser: https://wheat.pw.usda.gov/jb/?data=/ggds/oat-insularis; A. insularis data download: https://wheat.pw.usda.gov/GG3/content/avena-insularis-download. The mass spectrometry proteomics data and ProteinPilot search result files have been deposited to MassIVE (UCSD, San Diego, CA, USA; https://massive.ucsd.edu) under accession number MSV000088727. The publicly available OT3098 oat genome data was generated by PepsiCo and Corteva Agriscience. This dataset (annotation version 2) has been obtained and is available from GrainGenes: https://wheat.pw.usda.gov/GG3/content/pepsico-ot3098-hexaploid-oat-version-2-genome-assembly-release-collaboration-graingenes.
Databases used in this study include PTREP release 19, Uniref download 2019-09-03, Pfam download 2019-09-03, Swiss-Prot, TAIR, TrEMBL, REdat_9.9_Poaceae section of the PGSB transposon library, Immune Epitope Database and Analysis Resource (https://www.iedb.org), PLACE and PlantCare promoter motif databases, and pfam2GO.

Field-specific reporting

Please select the one below that is the best fit for your research. If you are not sure, read the appropriate sections before making your selection.

☒ Life sciences ☐ Behavioural & social sciences ☐ Ecological, evolutionary & environmental sciences

For a reference copy of the document with all sections, see nature.com/documents/nr-reporting-summary-flat.pdf

Life sciences study design

All studies must disclose on these points even when the disclosure is negative.

Sample size	No statistical methods were used to establish sample size for genome sequencing and assembly. The two progenitor Avena accessions were chosen as the likely descendant of the hexaploid A, C and D subgenomes based on previous marker data analysis. The Sang cultivar was chosen as a representative Spring oat cultivar and to facilitate single gene mapping in a closely related TILLING population.
Data exclusions	All sequencing data generated and reported as raw data was used in the genome assembly and analyses.
Replication	In all analyses that support the genome assemblies, gene expression, proteomics, GC/MS and SEM, the number of replicates or iterations are indicated in materials and methods or supplemental tables. In each case, replications were successful and used.<br>The genome assemblies themselves were validated using multiple methods i.e. BUSCO, genetic maps, HiC, and for A. sativa multiple comparisons to oat long-read assembly OT3098 were performed. This helped validate the other approaches.
Randomization	Randomization does not directly apply to the genome sequencing and assembly. However it does apply to some of the analyses conducted. In these cases, the group design and data seeding for computational analysis are described in the materials and methods and adhere to widely accepted standards. For example, bootstraping was applied to all phylogenies computed (e.g. Fig. 3a).
Blinding	Blinding does not apply to this study, as the study focuses on genome sequencing. This study focuses on plants genomics and the results of the study are not impacted by the concealment of treatment, data, or groups.

Reporting for specific materials, systems and methods

We require information from authors about some types of materials, experimental systems and methods used in many studies. Here, indicate whether each material, system or method listed is relevant to your study. If you are not sure if a list item applies to your research, read the appropriate section before selecting a response.

Materials & experimental systems

n/a	Involved in the study
☒	☐ Antibodies
☒	☐ Eukaryotic cell lines
☒	☐ Palaeontology and archaeology
☒	☐ Animals and other organisms
☒	☐ Human research participants
☒	☐ Clinical data
☒	☐ Dual use research of concern

Methods

n/a	Involved in the study
☒	☐ ChIP-seq
☒	☐ Flow cytometry
☒	☐ MRI-based neuroimaging
